# Atezolizumab plus anthracycline-based chemotherapy in metastatic triple-negative breast cancer: the randomized, double-blind phase 2b ALICE trial

**DOI:** 10.1038/s41591-022-02126-1

**Published:** 2022-12-08

**Authors:** Andreas Hagen Røssevold, Nikolai Kragøe Andresen, Christina Annette Bjerre, Bjørnar Gilje, Erik Hugger Jakobsen, Sunil Xavier Raj, Ragnhild Sørum Falk, Hege Giercksky Russnes, Thea Jahr, Randi Ruud Mathiesen, Jon Lømo, Øystein Garred, Sudhir Kumar Chauhan, Ragnhild Reehorst Lereim, Claire Dunn, Bjørn Naume, Jon Amund Kyte

**Affiliations:** 1 Department of Clinical Cancer Research, Oslo University Hospital, Oslo, Norway; 2 Department of Cancer Immunology, Institute for Cancer Research, Oslo University Hospital, Oslo, Norway; 3 Department of Oncology, Rigshospitalet, Copenhagen, Denmark; 4 Department of Hematology and Oncology, Stavanger University Hospital, Stavanger, Norway; 5 Department of Oncology, Sygehuset Lillebælt, Vejle, Denmark; 6 Department of Oncology, St. Olav University Hospital, Trondheim, Norway; 7 Oslo Centre for Biostatistics and Epidemiology, Oslo University Hospital, Oslo, Norway; 8 Department of Pathology, Oslo University Hospital, Oslo, Norway; 9 Department of Radiology and Nuclear medicine, Oslo University Hospital, Oslo, Norway; 10 Department of Oncology, Oslo University Hospital, Oslo, Norway; 11 Institute of Clinical Medicine, University of Oslo, Oslo, Norway

**Keywords:** Drug development, Breast cancer, Cancer immunotherapy

## Abstract

Immune checkpoint inhibitors have shown efficacy against metastatic triple-negative breast cancer (mTNBC) but only for PD-L1^positive^ disease. The randomized, placebo-controlled ALICE trial (NCT03164993) evaluated the addition of atezolizumab (anti-PD-L1) to immune-stimulating chemotherapy in mTNBC. Patients received pegylated liposomal doxorubicin (PLD) and low-dose cyclophosphamide in combination with atezolizumab (atezo-chemo; *n* = 40) or placebo (placebo-chemo; *n* = 28). Primary endpoints were descriptive assessment of progression-free survival in the per-protocol population (>3 atezolizumab and >2 PLD doses; *n* = 59) and safety in the full analysis set (FAS; all patients starting therapy; *n* = 68). Adverse events leading to drug discontinuation occurred in 18% of patients in the atezo-chemo arm (7/40) and in 7% of patients in the placebo-chemo arm (2/28). Improvement in progression-free survival was indicated in the atezo-chemo arm in the per-protocol population (median 4.3 months versus 3.5 months; hazard ratio (HR) = 0.57; 95% confidence interval (CI) 0.33–0.99; log-rank *P* = 0.047) and in the FAS (HR = 0.56; 95% CI 0.33–0.95; *P* = 0.033). A numerical advantage was observed for both the PD-L1^positive^ (*n* = 27; HR = 0.65; 95% CI 0.27–1.54) and PD-L1^negative^ subgroups (*n* = 31; HR = 0.57, 95% CI 0.27–1.21). The progression-free proportion after 15 months was 14.7% (5/34; 95% CI 6.4–30.1%) in the atezo-chemo arm versus 0% in the placebo-chemo arm. The addition of atezolizumab to PLD/cyclophosphamide was tolerable with an indication of clinical benefit, and the findings warrant further investigation of PD1/PD-L1 blockers in combination with immunomodulatory chemotherapy.

## Abstract (web summary)

In patients with metastatic triple-negative breast cancer, atezolizumab combined with immunogenic, anthracycline-based metronomic chemotherapy improves progression-free survival, including in patients with PD-L1^negative^ disease.

## Main

The prognosis for patients with metastatic triple-negative breast cancer (mTNBC) is poor, with a median survival of approximately 1 year, and the therapeutic options are limited^1^. Immune checkpoint inhibitors (ICIs) targeting PD1/PD-L1 are effective against metastatic disease in many cancer forms but have limited efficacy against mTNBC as monotherapy^2^. In combination with chemotherapy, atezolizumab (anti PD-L1) and pembrolizumab (anti-PD1) have shown activity against PD-L1^positive^ mTNBC^3,4^. Atezolizumab in combination with nab-paclitaxel and pembrolizumab in combination with nab-paclitaxel, paclitaxel or gemcitabine/carboplatin are currently approved for this indication by the European Medicines Agency, whereas only the pembrolizumab combinations are approved by the US Food & Drug Administration. The approval for atezolizumab in the United States was withdrawn in August 2021 after the publication of negative data from IMpassion131 (ref. ^5^) and positive data from KEYNOTE-355 (ref. ^4^). IMpassion130 was the first trial to demonstrate an effect of adding immunotherapy to chemotherapy in mTNBC. This trial indicated that atezolizumab produced a survival benefit when combined with nab-paclitaxel and that the effect applied only to PD-L1^positive^ disease^3^. Intriguingly, atezolizumab did not show any effect against mTNBC in IMpassion131 (ref. ^5^), where the chemotherapy backbone was paclitaxel. These contrasting findings have highlighted questions about how ICI activity is influenced by the chemotherapy backbone and by the use of steroids. Importantly, the addition of PD1/PD-L1 inhibitors to chemotherapy has not shown any efficacy against PD-L1^negative^ mTNBC, which represents a large proportion of patients^3,5,6^.

Doxorubicin and cyclophosphamide are described as potent inducers of immunogenic cell death^7–9^, and there is evidence suggesting that their pro-survival effect in patients depends on the immune response^7,10^. There is yet no robust evidence showing that these or other chemotherapies perceived to be immunogenic yield clinically relevant synergies with ICIs, as has been hypothesized^11^. A few interesting observations have, however, been reported. In the TONIC trial, induction therapy with doxorubicin yielded the highest response rate to nivolumab, and there was some evidence of immune activation in the tumor^12^. In the neoadjuvant setting, the KEYNOTE-522, GeparNuevo and IMpassion031 trials all demonstrated significantly increased response rates from adding anti-PD1/PD-L1 to chemotherapy^13–15^, whereas the NEOTRIP trial did not^16^. GeparNuevo indicated a benefit only for those starting anti-PD-L1 therapy 2 weeks before chemotherapy. Intriguingly, the chemotherapy backbone in KEYNOTE-522, GeparNuevo and IMpassion031 contained anthracyclines and cyclophosphamide, whereas NeoTRIP employed only taxanes and carboplatin before surgery. However, it is not known if these observations are causally related to the chemotherapy. The pathological complete response rate was relatively high (60%) in the one-armed NeoPACT trial, which combined pembrolizumab with an anthracycline-free regimen^17^.

Here we report the results of the randomized, double-blind, placebo-controlled phase 2b study ALICE (NCT03164993), which evaluated the safety and efficacy of adding atezolizumab to pegylated liposomal doxorubicin (PLD) combined with low-dose metronomic cyclophosphamide in patients with mTNBC^18^. This chemotherapy was selected based on the hypothesis that it would trigger ICI sensitivity in patients who are otherwise non-responsive. Low-dose cyclophosphamide is reported to decrease the levels of regulatory T cells (Tregs)^19^. This has led to an interest in using low-dose cyclophosphamide in cancer vaccine trials, yielding partially contradictory findings^20–23^. The pegylated liposomal form of doxorubicin was selected to obviate the need for steroids, minimize the adverse effects (AEs) on the heart and allow for continued treatment beyond otherwise mandatory anthracycline limits. To improve toxicity control and limit deep lymphopenia, which may preclude ICI activity, PLD was administered every 2nd week instead of every 4th week, with an option for dose reduction.

Inclusion of patients both with and without PD-L1 expression in tumors was allowed in this trial, to investigate both populationsʼ response to PD-L1 blockade when added to the selected chemotherapy. Stratification for PD-L1 was not performed at inclusion, because no PD-L1 test was in use for TNBC at the study sites at the time the study was initiated. The primary study objectives were the descriptive assessment of safety and efficacy, as measured by progression-free survival (PFS). The primary hypotheses were that the atezolizumab-chemotherapy (atezo-chemo) arm would have prolonged PFS compared to the control arm and that the atezo-chemo combination had acceptable safety and tolerability. PFS was measured in the per-protocol (PP) population and the PD-L1^positive^ PP population as primary analyses and in the full analysis set (FAS) and the PD-L1^negative^ PP population as secondary analyses. The secondary study objectives included assessment of objective response rate (ORR), duration of response (DoR), durable response rate (DRR), clinical benefit rate (CBR), overall survival (OS), quality of life, candidate biomarkers and changes in immune cell populations. The protocol power estimates focused on durable clinical benefit, as measured by 15-month PFS. This milestone was chosen because chemotherapy responses rarely last that long in mTNBC, whereas durable responses are a hallmark of ICI activity^1,24^.

## Results

### Patient characteristics

The study recruited patients with mTNBC who were 18 years of age or older, with an Eastern Cooperative Oncology Group (ECOG) performance status of 0 or 1, who had received no more than one prior line of chemotherapy in the metastatic setting and had measurable disease according to Immunotherapy Response Evaluation Criteria in Solid Tumors (iRECIST)^25^. For patients who had received (neo)adjuvant treatment with anthracyclines or cyclophosphamide, a disease-free interval of at least 12 months was required. Key exclusion criteria were central nervous system (CNS) disease (except asymptomatic lesions) and autoimmune disease requiring systemic treatment. A full list of inclusion and exclusion criteria is presented in the Methods section.

**Fig. 1:**
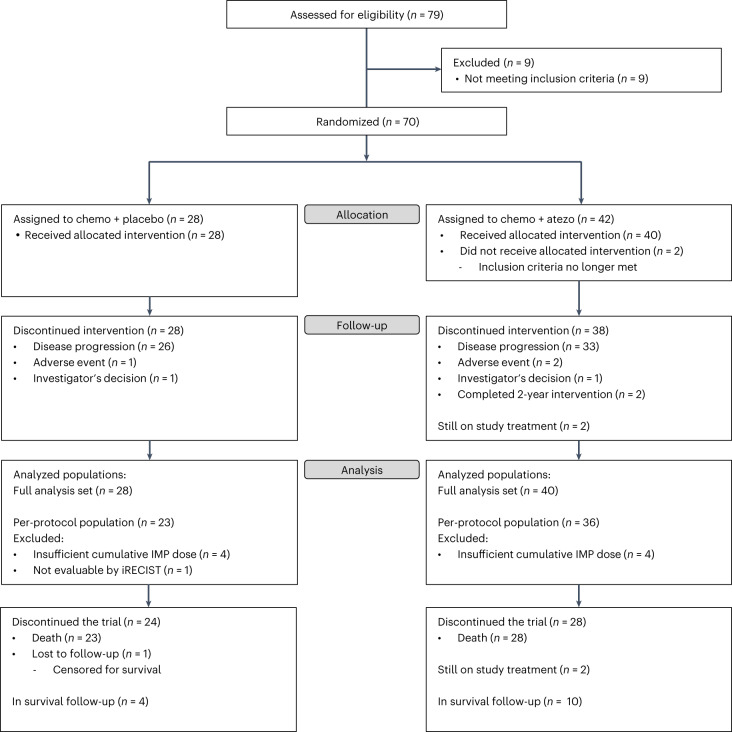
Patient flow diagram. The FAS included all patients who had received any IMP. The PP population comprised all patients who had received >3 doses of atezolizumab/placebo and >2 doses of PLD and could be evaluated for tumor response.

Patients were enrolled at five centers in Norway and Denmark. Between 24 August 2017 and 21 December 2021, 70 patients were randomly assigned to receive atezolizumab and chemotherapy (atezo-chemo; *n* = 42 (60%)) or placebo and chemotherapy (placebo-chemo; *n* = 28 (40%)). A CONSORT flow diagram is shown in Fig. 1. The 68 patients who received at least one dose of the allocated treatment were included in the FAS (40 in the atezo-chemo group and 28 in the placebo-chemo group), and 59 patients were included in the PP population receiving >3 atezolizumab/placebo doses and >2 PLD doses (36 in the atezo-chemo group and 23 in the placebo-chemo group).

Patient characteristics at baseline were mostly well-balanced between the treatment arms (Table 1). The atezo-chemo group had a lower proportion of patients with ECOG performance status 0, a higher median age, a lower proportion with liver metastases, a lower proportion with more than two metastatic sites and a higher proportion with PD-L1^positive^ disease.

**Table 1:** Demographics and baseline characteristics of the study cohort

	FAS			PP population		
	Placebo-chemo	Atezo-chemo	*P* value	Placebo-chemo	Atezo-chemo	*P* value
	(*n* = 28)	(*n* = 40)		(*n* = 23)	(*n* = 36)	
Age			0.16			0.17
Median (range)	52.5 (28.0–74.0)	58.5 (31.0–77.0)		52.0 (28.0–74.0)	59.0 (31.0–77.0)	
Gender			0.40			0.42
Female	28 (100%)	39 (97.5%)		23 (100%)	35 (97.2%)	
Male	0 (0%)	1 (2.5%)		0 (0%)	1 (2.8%)	
ECOG performance status			0.50			0.99
0	21 (75.0%)	27 (67.5%)		16 (69.6%)	25 (69.4%)	
1	7 (25.0%)	13 (32.5%)		7 (30.4%)	11 (30.6%)	
De novo metastatic disease			0.74			0.93
Yes	8 (28.6%)	10 (25.0%)		6 (26.1%)	9 (25.0%)	
No	20 (71.4%)	30 (75.0%)		17 (73.9%)	27 (75.0%)	
Bone metastases			0.23			0.18
Yes	16 (57.1%)	17 (42.5%)		13 (56.5%)	14 (38.9%)	
No	12 (42.9%)	23 (57.5%)		10 (43.5%)	22 (61.1%)	
Liver metastases			0.17			0.36
Yes	13 (46.4%)	12 (30.0%)		9 (39.1%)	10 (27.8%)	
No	15 (53.6%)	28 (70.0%)		14 (60.9%)	26 (72.2%)	
Lung metastases			0.44			0.69
Yes	10 (35.7%)	18 (45.0%)		9 (39.1%)	16 (44.4%)	
No	18 (64.3%)	22 (55.0%)		14 (60.9%)	20 (55.6%)	
Lymph node metastases			0.49			0.94
Yes	13 (46.4%)	22 (55.0%)		13 (56.5%)	20 (55.6%)	
No	15 (53.6%)	18 (45.0%)		10 (43.5%)	16 (44.4%)	
CNS metastases			0.80			0.75
Yes	1 (3.6%)	1 (2.5%)		1 (4.3%)	1 (2.8%)	
No	27 (96.4%)	39 (97.5%)		22 (95.7%)	35 (97.2%)	
Number of metastatic sites			0.24			0.12
≤2	15 (53.6%)	27 (67.5%)		12 (52.2%)	26 (72.2%)	
>2	13 (46.4%)	13 (32.5%)		11 (47.8%)	10 (27.8%)	
>3	3 (10.7%)	7 (17.5%)		3 (13.0%)	5 (13.9%)	
Line of chemotherapy			0.81			0.37
1st	16 (57.1%)	24 (60.0%)		12 (52.2%)	23 (63.9%)	
2nd	12 (42.9%)	16 (40.0%)		11 (47.8%)	13 (36.1%)	
Previous anthracycline treatment			0.90			0.71
Yes	20 (71.4%)	28 (70.0%)		17 (73.9%)	25 (69.4%)	
No	8 (28.6%)	12 (30.0%)		6 (26.1%)	11 (30.6%)	
PD-L1 status			0.22			0.22
Negative	17 (60.7%)	19 (47.5%)		14 (60.9%)	17 (47.2%)	
Positive	10 (35.7%)	21 (52.5%)		8 (34.8%)	19 (52.8%)	
Missing	1 (3.6%)	0 (0%)		1 (4.3%)	0 (0%)	
Intrinsic breast cancer subtype			0.44			0.48
Luminal A	2 (7.1%)	0 (0%)		2 (8.7%)	0 (0%)	
Luminal B	1 (3.6%)	1 (2.5%)		1 (4.3%)	1 (2.8%)	
HER2-enriched	2 (7.1%)	4 (10.0%)		2 (8.7%)	4 (11.1%)	
Basal	12 (42.9%)	22 (55.0%)		11 (47.8%)	20 (55.6%)	
Missing	11 (39.3%)	13 (32.5%)		7 (30.4%)	11 (30.6%)	
***BRCA*** **mutation status**			0.54			0.54
*BRCA1* mutation	1 (3.6%)	2 (5.0%)		1 (4.3%)	2 (5.6%)	
Normal variant	12 (42.9%)	22 (55.0%)		11 (47.8%)	22 (61.1%)	
Missing	15 (53.6%)	16 (40.0%)		11 (47.8%)	12 (33.3%)	

Two-sided *P* values were calculated using the Wilcoxon rank-sum test to compare age distributions and the chi-square test for all other variables. In comparison of the number of metastatic sites, only the first two groups were included in the chi-square test owing to the overlap between the two latter groups.

### Primary efficacy assessment

**Fig. 2:**
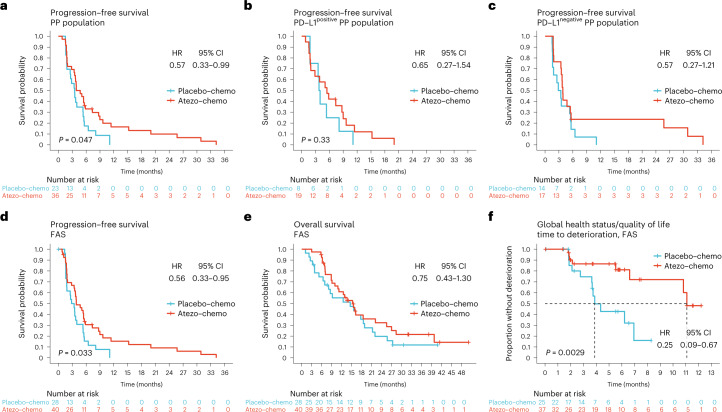
Kaplan–Meier plots of survival outcomes and quality of life. **a**–**d**, PFS assessed according to iRECIST in the PP population (**a**), the PD-L1^positive^ (**b**) and PD-L1^negative^ (**c**) PP population subsets and the FAS (**d**). **e**, OS in the FAS. **f**, Time to deterioration (reduction ≥20 points) of the global health status/quality of life score in the EORTC QLQ-C15-PAL questionnaire. The analysis includes the 62 patients (91%) in the FAS for whom a baseline value was available. The proportions of patients who completed this item at each of the subsequent timepoints are available in Extended Data Table 4. HRs with CIs were estimated using the Cox proportional hazards method. *P* values were calculated by the log-rank method.

At the data cutoff on 5 July 2022, the median follow-up time was 32.2 months (interquartile range (IQR) 27.4–40.9 months). A PFS event had occurred in 57 patients (96.6%) in the PP population (23/23 in the placebo-chemo group and 34/36 in the atezo-chemo group). All PFS events were caused by disease progression. PFS in the PP population was improved in the atezo-chemo arm compared to the placebo-chemo arm (hazard ratio (HR) = 0.57; 95% confidence interval (CI) 0.33–0.99; *P* = 0.047; Fig. 2a), as hypothesized for the descriptive primary efficacy endpoint. Median PFS was 4.3 months in the atezo-chemo arm versus 3.5 months in the placebo-chemo arm. The proportion without progression or death 15 months after randomization was 14.7% (95% CI 6.4–30.1%) in the atezo-chemo group and 0% in the placebo-chemo group. PFS was numerically improved in the PD-L1^positive^ population (co-primary endpoint; HR = 0.65, 95% CI 0.27–1.54; Fig. 2b).

### Safety assessment and treatment exposure

Table 2 gives a summary of the safety data. A list of all AEs is available in Supplementary Table 1. The median treatment period was 5.2 months (IQR 2.3–7.2) in the atezo-chemo arm and 3.2 months (IQR 2.0–5.7) in the placebo-chemo arm. The median number of PLD doses in the atezo-chemo arm was 11 (IQR 6–16) compared to seven (IQR 5–13) in the placebo-chemo arm. An AE of any grade was recorded in 100% of patients in both arms. Grade 3–4 AEs occurred in 62% of patients in the atezo-chemo group and in 43% of patients in the placebo-chemo group. The most common grade 3–4 AEs were decreased lymphocyte count (15% in atezo-chemo and 18% in placebo-chemo) and rash (18% in atezo-chemo and 0% in placebo-chemo). Serious adverse events (SAEs) occurred in 48% of patients in the atezo-chemo group and in 29% of patients in the placebo-chemo group. There were no deaths related to AEs. AEs leading to permanent discontinuation of any study drug occurred in 18% of patients in the atezo-chemo group and in 7% of patients in the placebo-chemo group, whereas atezo/placebo was discontinued due to an AE in 12% and 4%, respectively. Immune-related adverse events (irAEs) of any grade were recorded in 28% and 18% of patients in the atezo-chemo and placebo-chemo arms, respectively. Grade 3–4 irAEs occurred in 10% of patients in the atezo-chemo arm compared to 4% of patients in the placebo-chemo arm. The most common irAEs in the atezo-chemo arm were hypothyroidism (12%), pneumonitis (10%) and rash (8%) (Table 2). The treatment was considered to be tolerable.

**Table 2:** Summary of AEs (FAS)

	Placebo-chemo		Atezo-chemo	
	*n* = 28		*n* = 40	
	Any grade	Grade 3–4	Any grade	Grade 3–4
Any AE	100% (28)	43% (12)	100% (40)	62% (25)
Any SAE	29% (8)	18% (5)	48% (19)	38% (15)
Any IMP discontinued due to AE	7% (2)	4% (1)	18% (7)	10% (4)
Atezo/placebo discontinued due to AE	4% (1)	0	12% (5)	10% (4)
irAE				
Any	18% (5)	4% (1)	28% (11)	10% (4)
Hypothyroidism	7% (2)	0	12% (5)	0
Pneumonitis	4% (1)	0	10% (4)	5% (2)
Rash	4% (1)	0	8% (3)	5% (2)
Hyperthyroidism	7% (2)	0	2% (1)	0
Pancreatic enzymes increased	4% (1)	4% (1)	0	0
Pancreatitis	0	0	2% (1)	2% (1)
Pyrexia	0	0	2% (1)	2% (1)
AEs occurring in ≥25% in either group				
Rash	39% (11)	0	65% (26)	18% (7)
Nausea	54% (15)	0	57% (23)	5% (2)
Palmar-plantar erythrodysaesthesia syndrome	11% (3)	4% (1)	52% (21)	8% (3)
Fatigue	43% (12)	0	50% (20)	5% (2)
Mucosal inflammation	21% (6)	0	48% (19)	0
Constipation	43% (12)	0	45% (18)	0
Lymphocyte count decreased	36% (10)	18% (5)	45% (18)	15% (6)
Musculoskeletal pain	21% (6)	0	30% (12)	5% (2)

AEs were graded according to CTCAE version 4.0.

### Secondary efficacy and biomarker endpoints

**Extended Data Fig. 1:**
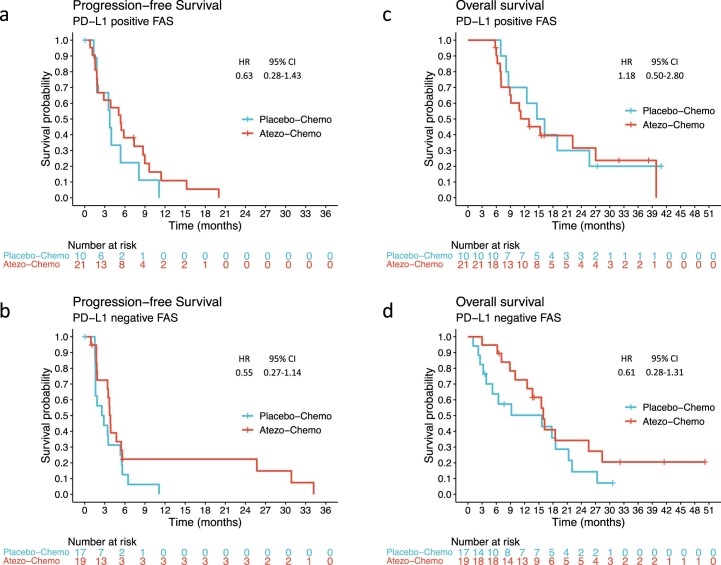
Kaplan-Meier plots of progression-free and overall survival by PD-L1 status (FAS). Progression-free survival by iRECIST in the PD-L1 positive **(a)** and negative **(b)** subgroups of the full analysis set. Overall survival in the PD-L1 positive **(c)** and negative **(d)** full analysis set. Hazard ratios with confidence intervals were calculated using the Cox proportional hazards method. PD-L1, Programmed death-ligand 1; FAS, full analysis set; HR, hazard ratio; CI, confidence interval.

**Extended Data Fig. 2:**
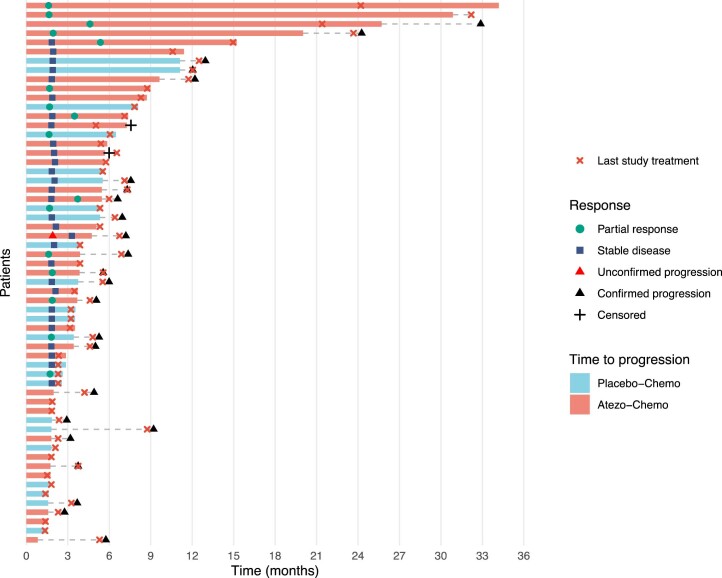
Swimmer plot. Duration of treatment and treatment responses in the PP population. The length of the bars represent the time from randomization to disease progression (iRECIST) or censoring. Symbols denote the first time a treatment response (other than progression) is documented, patients censored for progression, and the last date of study treatment.

PFS in the FAS (*n* = 68) was improved in the atezo-chemo arm compared to the placebo-chemo arm (HR = 0.56; 95% CI 0.33–0.95, *P* = 0.033; Fig. 2d). OS and PFS for the PD-L1^positive^ and PD-L1^negative^ FAS are shown in Extended Data Fig. 1. In the PD-L1^negative^ subgroup, a numerical PFS improvement was observed in the PP population (HR = 0.57, 95% CI 0.27–1.21; Fig. 2c) and in the FAS (HR = 0.55, 95% CI 0.27–1.14; Extended Data Fig. 1b). Three patients, all in the atezo-chemo arm, had PFS beyond the scheduled treatment period of 2 years (Extended Data Fig. 2). These three long-term responders had PD-L1^negative^ disease. One patient in the atezo-chemo arm had a PFS of 4.7 months by iRECIST^25^ and 1.8 months by RECIST version 1.1 (ref. ^26^), whereas the estimates were the same by both criteria for all other patients (Extended Data Fig. 2). There was no significant difference in OS between the treatment groups (FAS: HR = 0.75, 95% CI 0.43–1.30; Fig. 2e). The time to deterioration of global health status/quality of life was improved in the atezo-chemo group (FAS: HR = 0.25; 95% CI 0.09–0.67; Fig. 2f).

**Fig. 3:**
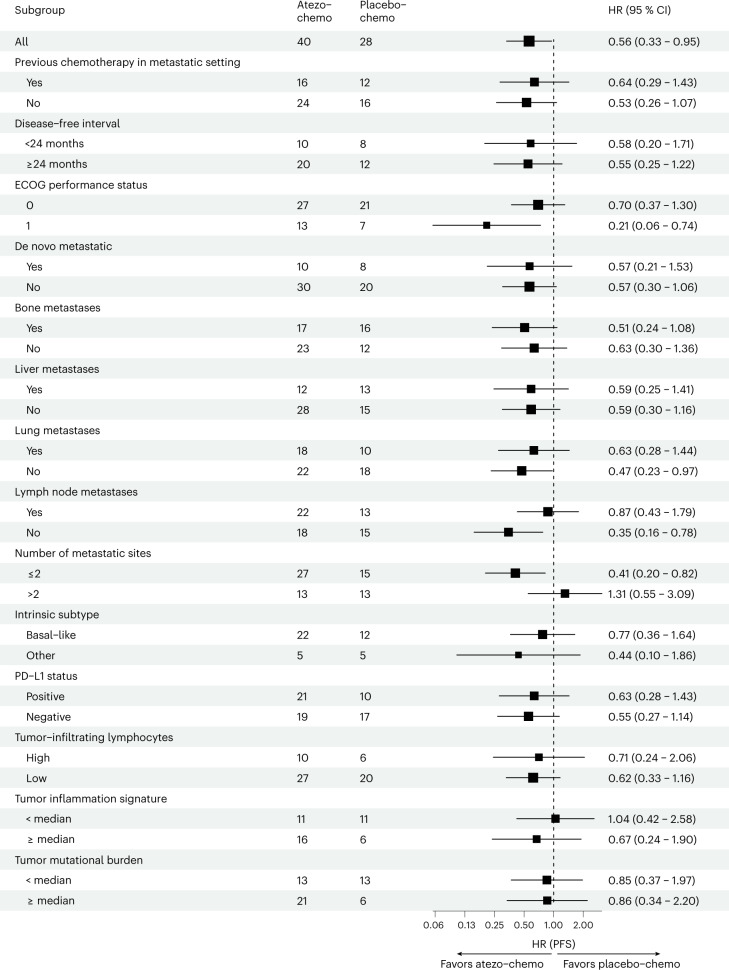
Subgroup analyses of PFS (FAS). HRs (center square) with 95% CIs (error bars) for PFS in the atezo-chemo group versus the placebo-chemo group in the FAS. HRs with CIs were calculated using the Cox proportional hazards method. The number of patients in each subgroup/arm is indicated. Intrinsic subtype, PD-L1 status, TIS, tumor lymphocyte infiltration and TMB were not available for all patients.

The PFS advantage for the atezo-chemo arm seemed consistent across the protocol-specified subgroups defined by clinical parameters (treatment line, disease-free interval, de novo metastatic disease and metastatic site) (Fig. 3). The only subgroup with an unfavorable HR in the stratified analyses was patients with more than two metastatic sites, which was not a pre-defined subgroup.

The ORR, CBR, DRR and DoR were numerically higher in the atezo-chemo arm, in both the PP population and the FAS (Extended Data Table 1). In the FAS, the ORR was 27.5% (6.1–42.8%) versus 17.9% (7.9–35.6), and the CBR was 50% (35.2–64.8) versus 35.7% (20.7–54.2). The DRR was 15.0% (7.1–29.1) in the atezo-chemo arm compared to 3.6% (0.2–17.7) in the placebo-chemo arm, whereas the median DoR was 7.3 months (IQR 2.1–19.6) versus 3.7 months (IQR 1.6–4.8). Among patients with PD-L1^negative^ disease, none recorded a durable response (>6 months) after placebo-chemo compared to 15.8% (5.5–37.6) in the atezo-chemo group (Extended Data Table 1).

We further investigated the potential impact of imbalances between the treatment arms by exploratory multivariable analyses performed in the FAS. For this purpose, we selected factors expected to be of particular clinical relevance and adjusted for one factor in each analysis (Extended Data Table 2). The unadjusted HR for PFS in the atezo-chemo-arm was 0.56, with a *P* value of 0.033. The adjusted HRs were 0.50, 0.60, 0.57 and 0.58 after inclusion of ECOG status, age, treatment line and PD-L1 status, respectively.

The pre-specified biomarker analyses comprised PD-L1 expression (SP142 immunohistochemistry (IHC) assay), tumor mutational burden (TMB) and the NanoString tumor inflammation signature (TIS)^27^ (immune gene expression). The median score was used as a cutoff for TIS and TMB. Whereas the PFS benefit from atezolizumab appeared not to depend on PD-L1 status or TMB, the HR was numerically better for patients with a high TIS score (Fig. 3). The TMB was generally low, with a median value of 2.14 mutations per megabase (mut/Mb; IQR 1.4–2.8). Only one patient, in the placebo-chemo group, had a TMB >10 mut/Mb.

### Exploratory assessment of effects on lymphocyte subsets

**Fig. 4:**
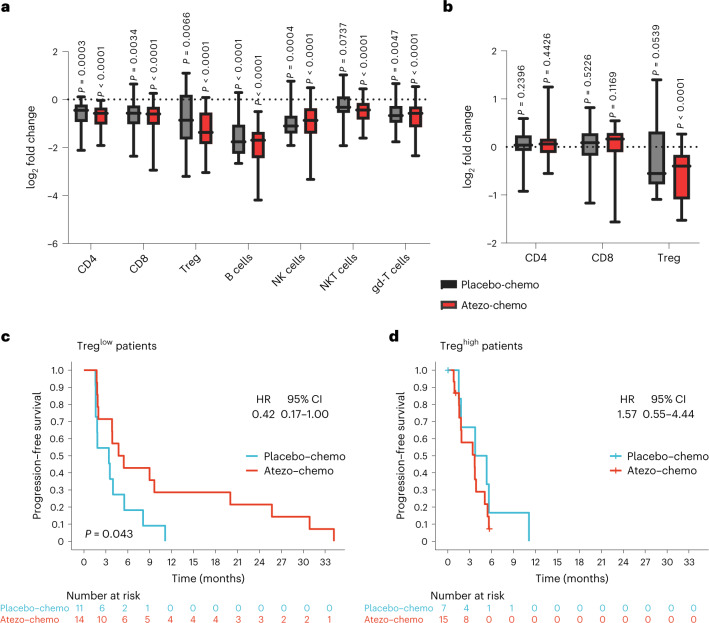
Effect of therapy on immune cell subsets and association of Tregs with PFS. PBMCs collected at pre-trial screening and after 8 weeks of trial treatment were assessed for different immune cell subsets by flow cytometry. All patients in the FAS for whom paired samples were available were included in the analysis (placebo-chemo *n* = 18, atezo-chemo *n* = 29). The gating strategy is shown in Supplementary Fig. 1. Cell counts were estimated by multiplying the percentage of each subset within the lymphocyte gate by the clinical lymphocyte differential cell count obtained for each patient at each timepoint. Fold changes from screening were calculated and log_2_-transformed. The baseline level is indicated with a dotted line. Data are presented as box and whisker plots, with the center line showing the median and the hinges showing the IQR. Whiskers show minimum and maximum values. **a**, Immune cell absolute cell counts. Immune cell subsets are defined as follows: CD4 T cells (CD3^+^CD4^+^CD8^−^), CD8 T cells (CD3^+^CD4^−^CD8^+^), Tregs (CD3^+^CD4^+^Foxp3^+^CD25^Hi^), B cells (CD3^−^CD19^+^), NK cells (CD3^−^CD56^+^), NKT cells (CD3^+^CD56^+^) and gd-T cells (CD3^+^gd-TCR^+^). **b**, Percentage of T cell subsets. CD4 and CD8 subsets are shown as a percentage of total lymphocytes, and Tregs are shown as a percentage of total CD4^+^ T cells. Two-tailed *P* values were calculated using the Wilcoxon matched-pairs signed-rank test. **c**,**d**, Kaplan–Meier analysis of PFS in the placebo-chemo group compared to the atezo-chemo group in patients with low (≤ median; **c**) and high (> median; **d**) levels of Tregs as percentage of total CD4^+^ T cells. Two-tailed *P* values were calculated using the log-rank test and HRs with CIs using the Cox proportional hazards method.

We hypothesized that the applied chemotherapy would cause a reduction in Treg levels, as reported for low-dose cyclophosphamide^19^. To evaluate how the chemotherapy with or without atezolizumab affected circulating immune cell subsets, paired samples of peripheral blood mononuclear cells (PBMCs) from two timepoints (pre-treatment and week 8) from 47 patients (all patients in the FAS with paired samples available) were assessed by an exploratory flow cytometry analysis. As expected, the absolute cell counts for all measured immune cell subsets decreased after treatment, with B cells and Tregs being most affected (Fig. 4a). Furthermore, we found that the percentage of Tregs as a fraction of CD4^+^ T cells decreased in both arms after therapy (Fig. 4b), with a larger decrease in mean value in the atezo-chemo arm (from 3.28% to 2.33%; *P* < 0.0001) than in the placebo-chemo arm (from 2.82% to 2.20%; *P* = 0.054).

### Exploratory biomarker analyses

**Extended Data Fig. 3:**
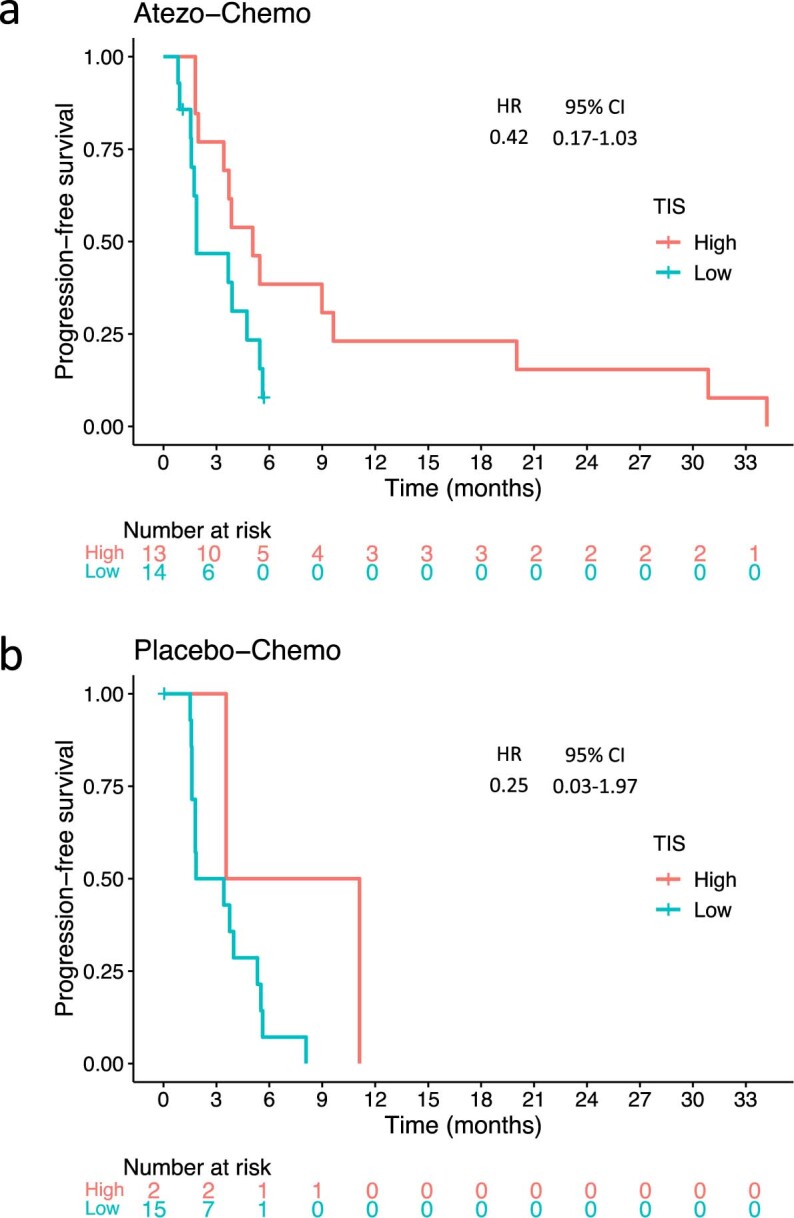
Kaplan-Meier plots of progression-free survival with high and low Tumor Inflammation Signature. Progression-free survival by iRECIST in the subpopulation with high (> 66^th^ percentile) and low (≤ 66^th^ percentile) tumor inflammation signature (TIS) in the atezo-chemo **(a)** and placebo-chemo **(b)** group. Tumor Inflammation Signature was assessed by analysis of pre-treatment biopsies on the NanoString BC360 assay. All patients in the full analysis set for whom samples were available were included in the analysis (placebo-chemo n = 17, atezo-chemo n = 27). Hazard ratios (HR) with confidence intervals (CI) were calculated using the Cox proportional hazards method.

In the analyses of pre-defined biomarkers, we found that only patients with above-median TIS appeared to benefit from the addition of atezolizumab (Fig. 3). Previous studies indicated that patients with values within the highest tertile (above the 66th percentile; TIS^high^) have improved responses to PD-1 blockers^28,29^. Applying the same cutoff (66th percentile) for an exploratory analysis in our dataset, we found that the TIS^high^ group appeared to have improved PFS in the atezo-chemo arm, with an HR of 0.42 (95% CI 0.17–1.03), and comprised all atezo-chemo patients with a PFS >12 months (Extended Data Fig. 3).

Exploratory analyses suggested that patients with low Treg levels at baseline had a substantial PFS benefit in the atezo-chemo arm (HR = 42, 95% CI 0.17–1.00, *P* = 0.043; Fig. 4c), whereas those with high Treg levels had no benefit (Fig. 4d). The level of tumor-infiltrating lymphocytes (TILs) was also evaluated in an explorative biomarker analysis, by assessment of biopsies obtained at study entry, and scored as TIL^low^ (*n* = 47) or TIL^high^ (*n* = 16). As shown in Fig. 3, the observed PFS benefit for the atezo-chemo arm was relatively similar for the TIL^low^ (HR = 0.62) and TIL^high^ (HR = 0.71) subgroups.

**Extended Data Fig. 4:**
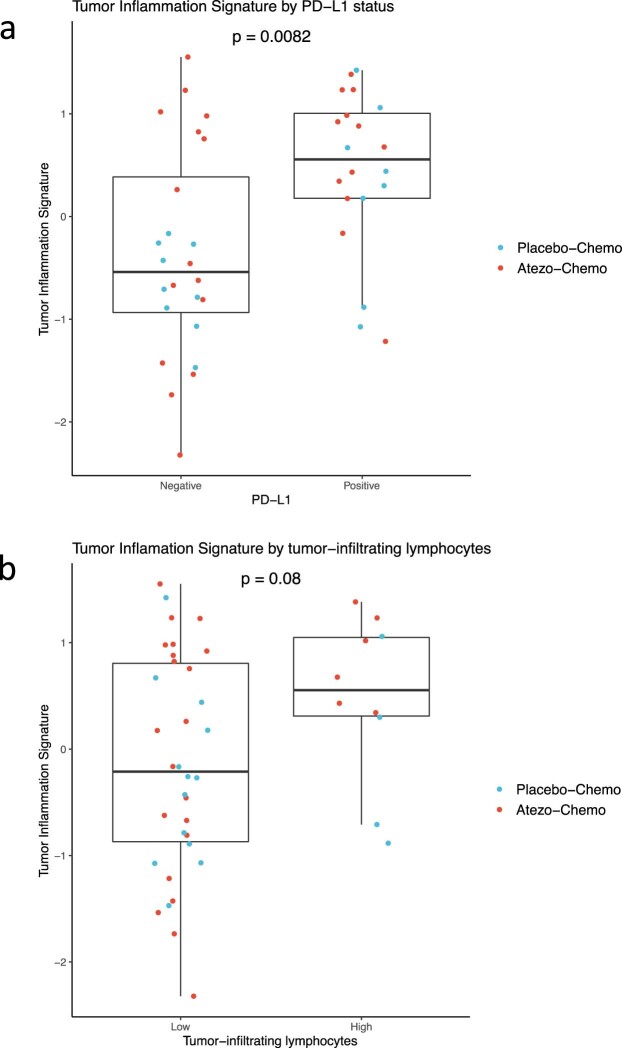
Correlation between Tumor Inflammation Signature and other biomarkers. **a:** Tumor Inflammation Signature (TIS) in patients with PD-L1 negative and positive tumors. Both analyses were done on archival pre-study biopsies. For 28 of the 44 patients, the analyses were done in the same tumor sample, while 16 of the tumors analyzed for PD-L1 did not contain enough material to be analyzed for TIS. For these biopsies, the Nanostring assay was performed on a different archival biopsy. **b:** TIS in patients with low and high infiltration of lymphocytes in tumor. Tumor-infiltrating lymphocyte (TIL) abundance was assessed in study-specific pre-treatment biopsies, whereas TIS was analyzed in archival biopsies, due to the limited volume of the screening biopsies. Tumor Inflammation Signature was assessed by a single analysis of pre-treatment biopsies on the NanoString BC360 assay. All patients for whom a suitable archival biopsy was available were included in the analysis (n = 44). PD-L1 expression (SP142 ASSAY) and TIL were assessed once for each biopsy, by two experienced breast cancer pathologists that were blinded for treatment arm and clinical outcome. Two-sided p-values were calculated using the Wilcoxon rank sum test. In the box plots, the center line represents the median value and the box limits represent the upper and lower quartiles. The whiskers extends to the extreme values, omitting outliers extending > 1.5 x IQR from the box limits, these are shown in the dot plot. TIS, Tumor Inflammation Signature; PD-L1, programmed death-ligand 1; TIL, tumor-infiltrating lymphocytes.

We further explored how the TIS correlated with the PD-L1 status and TIL score. The TIS and PD-L1 analyses were performed on the same biopsy in all cases where enough material was available (28/44 biopsies), whereas the TIL scoring was performed on a different biopsy. An overview of the biopsy sites is given in Extended Data Table 3. The results showed that the TIS correlated with PD-L1 status (*P* = 0.0082), with a median scaled TIS value of 0.56 (IQR 0.18–1.00) for PD-L1^positive^ versus −0.54 for PD-L1^negative^ (IQR −0.93 to 0.39) (Extended Data Fig. 4). A proportion of 25% in the PD-L1^negative^ group had a TIS above the median value in the PD-L1^positive^ group. The median scaled TIS was 0.55 and −0.21 in the TIL^high^ and TIL^low^ groups, respectively. A proportion of 32% in the TIL^low^ group had a TIS above the median value in the TIL^high^ group.

## Discussion

There is no consensus on the optimal chemotherapy regimen for combination with ICI, and there is a need to investigate whether immunologically cold tumors can be made susceptible to ICI therapy. This is a clinically important knowledge gap for mTNBC, which has a poor prognosis and where ICI benefits have been observed only for PD-L1^positive^ disease. The ALICE trial is, to our knowledge, the first randomized study in mTNBC investigating the addition of any ICI to anthracyclines, even though anthracyclines are widely used for this disease. There was a plausible rationale for exploring the selected combination, based on the perceived immunogenic properties of anthracyclines, the avoidance of steroids with PLD and the reported effects of low-dose cyclophosphamide on Tregs. The results indicate improved therapeutic efficacy for the atezo-chemo arm, as measured by the primary endpoint (descriptive comparison of PFS). The PFS HR benefit was numerically large and reached statistical significance both in the PP population and the FAS, even though the patient number was relatively small and lower than originally planned. The same applied to the proportion of patients without disease progression after 15 months. The trial was not powered to test the superiority hypothesis of the addition of atezolizumab. Accordingly, the statistical analyses were descriptive, and the findings need to be confirmed in an independent study. There was no difference in the median OS, whereas the secondary efficacy endpoints related to tumor response (ORR, CBR, DRR and DoR) were in favor of the atezo-chemo arm, mostly with overlapping CIs. The development in the global health status score indicated a favorable effect of atezolizumab on the quality of life.

The nature, frequency and severity of AEs were as expected based on the properties of the study drugs and the advanced disease stage. We recorded a higher frequency of SAEs and high-grade AEs in the atezo-chemo arm. This may be due to toxicity from atezolizumab but may also reflect longer duration of treatment in the atezo-chemo group. A role of higher treatment exposure is supported by the increased frequency of non-immune-related AEs as well as the immune-related events. High-grade irAEs were observed in only 10% of the atezo-chemo group and were generally manageable. The proportion of patients who discontinued therapy due to AEs was in line with IMpassion130 (ref. ^3^) and IMpassion131 (ref. ^5^).

Whereas the improvement in median PFS was only modest, the indicated atezolizumab benefit was evident for the tail of the PFS curve, as reflected by the DRRs. This is in line with the experience from melanoma and other cancer forms where ICI has a proven benefit^24^. At the timepoint of 15 months, all patients without disease progression belonged to the atezolizumab arm. The number of patients remaining for these analyses was small, and the data should be interpreted with caution. The PFS signal of long-term efficacy is, however, worth noting as there is an unmet medical need in mTNBC for agents providing sustained benefit. On the other hand, even though the survival data are not mature, it appears evident that the median survival will remain similar between the arms. Further follow-up is ongoing to assess if a proportion of patients experience long-term survival.

ALICE was a small randomized study, which means that known and unknown imbalances between the arms represent a major limitation. We performed multivariable analyses for selected factors of perceived clinical significance. The results suggested that imbalances in these factors could not explain the indicated atezolizumab PFS benefit. The HR adjusted for ECOG indicated a larger PFS benefit for the atezo-chemo-arm than the parent analysis, whereas the other adjusted HRs were similar to the unadjusted value. In the subgroup analyses, the numerical HR advantage for the atezo-chemo group was conserved in all groups defined by clinical parameters, apart from those with more than two metastatic sites, which had a sample size of only 21 patients. The CIs were generally wide, due to the small sample size, but still seemed to suggest that ECOG >0, fewer than three metastatic sites and lack of lymph node involvement were associated with increased benefit from atezolizumab. There is no clear explanation for these observations, and we cannot exclude that they are incidental due to multiple testing.

Interestingly, the PFS advantage appears to apply even to the PD-L1^negative^ population, and the three patients with >24-month PFS in the PD-L1^negative^ group had all been randomized to the atezo-chemo arm. Atezolizumab was administered in the same dose as in IMpassion130 (ref. ^3^) and IMpassion131 (ref. ^5^). Furthermore, the enrollment criteria and patient characteristics in ALICE, IMpassion130 and IMpassion131 were mostly similar, apart from the allowance in the ALICE trial for one previous line of chemotherapy in the metastatic setting. The subgroup analyses from ALICE did not suggest that the treatment line affected the PFS advantage for the atezo-chemo arm. The ALICE data are, thus, consistent with the hypothesis that the applied chemotherapy may trigger ICI sensitivity in PD-L1^negative^ patients with mTNBC who are otherwise non-responsive. We have, as yet, no mechanistic evidence for this from ALICE patients and plan to investigate if the therapy modified the tumor microenvironment by analyzing study biopsies.

It should be emphasized that cross-trial comparisons of PD-L1^positive/negative^ populations are associated with uncertainty. In the ALICE trial, the PD-L1 assay, interpretation method and choice of biopsy were defined in the protocol and statistical analysis plan. We employed the same assay (SP142) and cutoff (≥1%) as used in IMpassion130 (ref. ^3^) and IMpassion131 (ref. ^5^). If the patient had positive and negative PD-L1 status in different biopsies, the most recent biopsy obtained before study therapy was used. The SP142 assay was chosen because it was recommended by Roche and reported to be more predictive for the effect of atezolizumab in mTNBC than other assays, which identify more patients as PD-L1^positive^ (ref. ^6^). The decision to use the last biopsy was based on the assumption that this is most relevant for the metastatic disease and was in accordance with the national guidelines for PD-L1 testing for mTNBC in Norway. However, the choice of biopsy varies between trials. In IMpassion130, it was not defined whether the biopsy should be from a primary tumor or metastasis^3^. In the SAFIR-02 trial, the SP142 assay was employed on a metastatic biopsy obtained <1 year before enrollment^30^. Data from the IMpassion130 cohort suggest that PD-L1 assessment on both primary and metastatic tumor is informative for atezolizumab benefit^31^. It has previously been reported that the PD-L1^positive^ proportion is higher in primary tumors, compared to metastatic biopsies^32^. Accordingly, it is possible that some of the patients classified as PD-L1^negative^ in ALICE and other trials could have had a PD-L1^positive^ primary tumor. The proportion of patients with PD-L1^positive^ disease in ALICE (47%) is in line with other mTNBC studies employing the SP142 assay, including IMpassion130 (41% PD-L1^positive^) and IMpassion131 (45% PD-L1^positive^)^3,5,30–32^. Accordingly, the chosen assay and observed PD-L1^positive^ percentage appear to suggest that the PD-L1^negative^ ALICE population is comparable to the PD-L1^negative^ groups in other mTNBC trials. The PD-L1 classification issue is, however, complicated, and further investigation of PD-L1 status across clinical trial cohorts would be of interest.

The TIS is based on the expression of 18 immune-related genes and was developed to predict response to anti-PD-1 therapy^27^. In our dataset, a high TIS score appeared to be associated with clinical benefit from atezolizumab, whereas a high TMB or PD-L1 positivity did not. Interestingly, all patients with a PFS of 12 months belonged to the top tertile of TIS, which is a cutoff reported by others^29^. Furthermore, the lack of association with TMB may reflect that the TMB was generally low. However, the data support the notion that gene expression signatures may identify a responsive subgroup that is not detected by regulatory approved diagnostic biomarker assays. This observation would be worth investigating in independent and larger cohorts, using TIS and other gene expression signatures. Whether or not the concept of immunogenic cell death applies, it is possible that some level of pre-existing immune activation in the tumor is required for the benefit of PD1/PD-L1 blockers. This form of immune activation may not necessarily lead to expression of PD-L1 but may be captured by gene expression assays. It will be important to investigate whether gene expression signatures vary between primary tumors and different metastatic lesions and how this compares to variations in PD-L1 status. A considerable discrepancy has been reported between biopsies obtained at different timepoints^31,32^. For clinical practice, there is a need to determine whether more than one biopsy is required for biomarker assessment and if more reproducible and informative assays can be established.

In our dataset, the benefit from atezolizumab was restricted to those with a below-median level of Tregs in blood. This was an exploratory biomarker test and needs further investigation and validation. A possible explanation may be that different suppressive mechanisms may be involved and that high Treg levels compensate for PD1/PD-L1-mediated suppression. There has been a large interest in countering Tregs in cancer therapy. We investigated the use of metronomic cyclophosphamide for this purpose. The previously reported data on cyclophosphamide and Tregs are sparse and partially contradictory^19–23^. It is, therefore, interesting that we observed a decrease in Tregs. Low-dose cyclophosphamide has only mild side effects and may be widely applicable across cancer forms. We do not know if the changes in peripheral blood are reflected in the tumor microenvironment and if the therapy affects Treg function. Further exploration of this is needed. It may also be noted that a decrease in Tregs is not necessarily beneficial as it may enhance autoimmunity.

The radiological response was assessed locally, without a central blinded review. This setup may have affected the consistency in response measurement across study sites but should not lead to a systematic bias in favor of any arm, as the study was placebo-controlled. Only one patient had a different timepoint for progression between iRECIST and RECIST version 1.1. The data, therefore, suggest that pseudo-progression^33^ was not a common feature.

Several chemotherapeutic agents have been hypothesized to induce immunogenic cell death. However, anthracyclines trigger release of four major damage‐associated molecular patterns^9^ and are considered to be particularly immunogenic^8,9,11^. As anthracyclines are also potent drugs against TNBC, it is important to establish whether synergy between anthracyclines and ICIs can be achieved and if this would make a larger proportion of patients with TNBC responsive to ICIs. Moreover, some patients are resistant to taxanes and, therefore, not candidates for therapy with the atezolizumab/nab-paclitaxel regimen. The discrepancy between the results from IMpassion130 and IMpassion131 have highlighted the need to document the efficacy of each ICI/chemotherapy combination. Pembrolizumab has been tested with other agents but not with anthracyclines, which is employed in many countries as first-line therapy for mTNBC.

PLD is more expensive than several other anthracyclines, and this is a hurdle for the implementation of the findings from the ALICE trial. On the other hand, continued PLD administration is feasible in long-term ICI responders, whereas other anthracyclines have mandatory restrictions on accumulative dose. Steroids are needed to control side effects of many agents, and their use is difficult to limit in a real-world setting, but they are rarely required for PLD therapy. The PLD dosing schedule used in ALICE (every 2nd week) is used for Kaposi sarcoma and has been explored in some breast cancer studies, with or without low-dose cyclophosphamide^34,35^, but is not regularly used for TNBC. This may represent a challenge for implementing the ALICE regimen, even if the cumulative dose over 4 weeks corresponds to common practice.

In conclusion, this study indicates that the addition of atezolizumab to PLD and low-dose cyclophosphamide improved PFS in patients with mTNBC. A benefit was also indicated in patients with PD-L1^negative^ disease. The combination therapy was tolerable, and no new safety signals were identified. Subgroup analyses suggest that a high immune gene expression in the tumor may predict benefit from atezolizumab. There was no difference in median survival, and it will be important to establish if there is a survival benefit for a proportion of the patients. The flow cytometry data support the hypothesis that low-dose metronomic cyclophosphamide leads to decreased Treg levels. The findings in this small randomized trial are consistent with the concept that ICI may synergize with selected immune-stimulating chemotherapy and provide a basis for further investigations in larger mTNBC cohorts. The results also suggest that similar immunomodulatory chemotherapies may be considered in combination with ICI in other cancer forms.

## Methods

### Study design and approvals

The ALICE trial was an investigator-initiated, multi-center, randomized, double-blind, parallel-group, placebo-controlled phase 2b trial evaluating the safety and efficacy of the combination of atezolizumab and chemotherapy in mTNBC. Oslo University Hospital was the sponsor. Protocol approval was obtained from the Regional Committee for Medical Research Ethics South-East Norway (EC ID: 14195), the Research Ethics Committee in Denmark (EC ID: H-18018750), the Norwegian Medical Agency (ID: 16/11993), the Danish Medicines Agency (ID: 2018051636) and institutional review boards. The trial was conducted according to the guidelines of Good Clinical Practice, the principles of the World Medical Association’s Declaration of Helsinki and the CONSORT 2010 guidelines. All patients provided written informed consent. Patients did not receive any financial compensation. The main contents of the protocol were published previously^18^. The last version of the protocol is available as Supplementary Material. Trial registration: NCT03164993; EudraCT: 2016-003570-40.

According to the initial protocol, the plan was to enroll 75 patients in the intention-to-treat (ITT) population and use this for all efficacy analyses as well as for the safety assessment. Owing to the small sample size and concerns that the scientific output of the study would be affected by patients leaving the trial before any therapeutic effect could be expected, or without response evaluation, the protocol was amended to allow the enrollment of 75 patients in a PP population. This decision was based on the consideration that the sample size of 75 was marginal, compared to the statistical estimates and the aim of comparing between the arms the proportion of long-term responders (>15-month PFS), which were expected to represent only a small number of patients (see ‘Statistical analysis’ below). Furthermore, we expected that patients leaving the trial very early would be less informative for the efficacy and toxicity of atezolizumab, and we wanted to decide up-front (in this double-blind trial) a definition of a more informative population. The amendment was implemented in March 2018, after enrollment of nine patients. The PP population comprised all patients who had received >3 doses of atezolizumab/placebo and >2 doses of PLD and could be evaluated for tumor response. PFS in the PD-L1^positive^ PP population was added as a co-primary outcome in a later amendment (protocol version 3.0, implemented in April 2021). Enrollment was stopped on 31 December 2021 owing to slow patient accrual, after the introduction of atezolizumab/nab-paclitaxel as standard therapy for PD-L1^positive^ mTNBC. Data lock and unblinding were performed 6 months after enrollment of the last patients to allow for a reasonable observation time. All protocol amendments were implemented before data lock and unblinding. No interim analyses were performed. Data lock was performed on 5 July 2022.

### Patients

Patients were enrolled in five hospitals in Norway and Denmark (Oslo University Hospital, Stavanger University Hospital, St. Olavs University Hospital (Trondheim), Vejle Hospital and Rigshospitalet (Copenhagen)).

The full inclusion and exclusion criteria are listed below.

Inclusion criteria

1. Metastatic or incurable locally advanced, histologically documented TNBC (negative for HER2, ER and PR). HER2 negativity is defined as either of the following by local laboratory assessment: in situ hybridization (ISH) non-amplified (ratio of *ERBB2* to CEP17 <2.0 or single probe average *ERBB2* gene copy number <4 signals per cell) or IHC 0 or IHC 1+ (if more than one test result is available and not all results meet the inclusion criterion definition, all results should be discussed with the principal investigator to establish eligibility of the patient). ER and PR negativity are defined as <1% and <10%, respectively, of cells expressing hormonal receptors via IHC analysis.
2. Adequate newly obtained core or excisional biopsy of a tumor lesion not previously irradiated. No anti-tumor treatment is allowed between the timepoint for biopsy and study entry. If a patient has undergone chemotherapy in the metastatic setting, a new biopsy must be obtained after this therapy.
3. Measurable disease according to iRECIST.
4. Signed informed consent form.
5. Women or men aged ≥18 years.
6. ECOG performance status of 0 or 1.
7. In patients who have received (neo)adjuvant treatment with anthracyclines or cyclophosphamide, a minimum of 12 months from treatment with anthracyclines or cyclophosphamide until relapse of disease is required.
8. A maximum of one previous line with chemotherapy in the metastatic setting.
9. Female patients of childbearing potential should have a negative urine or serum pregnancy test within 7 days before receiving the first dose of study medication. If the urine test is positive or cannot be confirmed as negative, a serum pregnancy test will be required.
10. Female patients of childbearing potential should agree to remain abstinent (refrain from heterosexual intercourse) or use contraceptive methods that result in a failure rate of <1% per year, during the treatment period and for at least 5 months after the last dose of study therapy. A woman is considered to be of childbearing potential if she is postmenarcheal, has not reached a postmenopausal state (≥12 continuous months of amenorrhea with no identified cause other than menopause) and has not undergone surgical sterilization (removal of ovaries and/or uterus). Examples of contraceptive methods with a failure rate of <1% per year include bilateral tubal ligation, male sterilization, proper use of hormonal contraceptives that inhibit ovulation and hormone-releasing intrauterine devices. Periodic abstinence (for example, calendar, ovulation, symptothermal or postovulation methods) and withdrawal are not acceptable methods of contraception.
11. Male patients should agree to use an adequate method of contraception starting with the first dose of study therapy through 3 months after the last dose of study therapy.
12. Able to swallow orally administrated medication.
13. Adequate organ function, defined as absolute neutrophil count ≥1.20 × 10^9^/L, lymphocyte count ≥0.50 × 10^9^/L, thrombocytes ≥80 × 10^9^/L, hemoglobin ≥9 g/dl (≥ 5.6 mmol/L), creatinine ≤1.5× the upper limit of normal (ULN) or glomerular filtration rate/creatinine clearance ≥40 ml/min, total bilirubin ≤1.5× ULN, AST and ALT ≤2.5× ULN (≤5× ULN for patients with liver metastases), albumin ≥2.5 g/dl and International Normalized Ratio (INR) or prothrombin time ≤1.5× ULN for patients not receiving anticoagulant therapy.

Exclusion criteria

1. Malignancies other than breast cancer within 5 years before randomization, with the exception of those with a negligible risk of metastasis or death and treated with expected curative outcome (such as adequately treated carcinoma in situ of the cervix or basal or squamous cell skin cancer).
2. Patients with known PD-L1^positive^ TNBC, as assessed by the Ventana SP142 assay (IC ≥1%) and no previous chemotherapy in the metastatic setting, should be offered standard therapy with nab-paclitaxel/atezolizumab outside of the trial, if they had a disease-free interval of >12 months after previous (neo)adjuvant chemotherapy, unless the patient for other reasons should not receive nab-paclitaxel, according to own preferences, drug availability or recommendations by the treating physician. A history of progression on taxanes in the neoadjuvant setting, or severe side effects from taxane therapy, may represent sufficient reason to offer the patient inclusion into the ALICE trial, if the physician considers that the patient should receive anthracyclines rather than taxanes as first-line therapy for metastatic disease. If more than one TNBC biopsy has been evaluated for PD-L1 by the SP142 assay, and the results differ, the patient’s PD-L1 status determination will be based on best clinical judgment.
3. Spinal cord compression not definitively treated with surgery and/or radiation or previously diagnosed and treated spinal cord compression without evidence that disease has been clinically stable for >2 weeks before randomization.
4. Known CNS disease, except for asymptomatic CNS metastases, provided that all of the following criteria are met:
  - Measurable disease outside the CNS.
  - No metastases to mesencephalon, pons, medulla oblongata or spinal cord.
  - No ongoing requirement for dexamethasone as therapy for CNS disease.
  - No radiation of brain lesions within 7 days before randomization.
  - No leptomeningeal disease.
  - Patients with symptomatic CNS metastases must receive radiation therapy and/or surgery for CNS metastases. After treatment, these patients may be eligible, if all other criteria are met.
5. Uncontrolled pleural effusion, pericardial effusion or ascites. Patients with indwelling catheters (for example, PleurX) are allowed.
6. Uncontrolled tumor-related pain. Patients requiring narcotic pain medication must be on a stable regimen at study entry. Symptomatic lesions (for example, bone metastases or metastases causing nerve impingement) amenable to palliative radiotherapy should be treated before randomization. Asymptomatic metastatic lesions whose further growth would likely cause functional deficits or intractable pain (for example, epidural metastasis that is not presently associated with spinal cord compression) should be considered for locoregional therapy if appropriate before randomization.
7. Ionized calcium >1.2× ULN. The use of bisphosphonates is allowed.
8. Pregnant or breastfeeding.
9. Evidence of significant uncontrolled concomitant disease that could affect compliance with the protocol or interpretation of results, including significant liver disease (such as cirrhosis, uncontrolled major seizure disorder or superior vena cava syndrome).
10. Significant cardiovascular disease, such as New York Heart Association (NYHA) cardiac disease (class II or higher), myocardial infarction within 3 months before randomization, unstable arrhythmias or unstable angina. Patients with a known left ventricular ejection fraction (LVEF) <40% will be excluded. Patients with known coronary artery disease, congestive heart failure not meeting the above criteria or LVEF <50% must be on a stable medical regimen that is optimized in the opinion of the treating physician, in consultation with a cardiologist if appropriate.
11. Severe infection within 14 days before randomization, requiring hospitalization.
12. Received oral or intravenous (i.v.) antibiotics within 1 week before cycle 1, day 1. Patients receiving routine antibiotic prophylaxis (for example, to prevent chronic obstructive pulmonary disease exacerbation or for dental extraction) are eligible.
13. Major surgical procedure within 14 days before randomization or anticipation of the need for a major surgical procedure during the course of the study other than for diagnosis. Placement of central venous access catheter(s) is not considered a major surgical procedure and is, therefore, permitted.
14. A history of severe allergic, anaphylactic or other hypersensitivity reactions to chimeric or humanized antibodies or fusion proteins.
15. Known hypersensitivity or allergy to biopharmaceuticals produced in Chinese hamster ovary cells or any component of the atezolizumab formulation.
16. Known hypersensitivity to doxorubicin or cyclophosphamide or any of their excipients.
17. A history of autoimmune disease that has required systemic treatment in the past 2 years (that is, with use of disease-modifying agents, corticosteroids or immunosuppressive drugs). Replacement therapy (for example, thyroxin, insulin or physiologic corticosteroid replacement therapy for adrenal or pituitary insufficiency) is not considered a form of systemic treatment. Patients with eczema, psoriasis, lichen simplex chronicus or vitiligo with dermatologic manifestations only (for example, no psoriatic arthritis) are permitted provided that they meet all of the following conditions:
  - Rash must cover less than 10% of body surface area.
  - Disease is well-controlled at baseline and only requiring low-potency topical steroids.
  - No acute exacerbations of underlying condition within the last 12 months (not requiring psoralen plus ultraviolet A radiation (PUVA), methotrexate, retinoids, biologic agents, oral calcineurin inhibitors or high-potency or oral steroids).

14. Undergone allogeneic stem cell or solid organ transplantation.
15. A history of idiopathic pulmonary fibrosis (including pneumonitis) drug-induced pneumonitis, organizing pneumonia (that is, bronchiolitis obliterans, cryptogenic organizing pneumonia) or evidence of active pneumonitis on screening chest CT scan. History of radiation pneumonitis in the radiation field (fibrosis) is permitted.
16. A positive test for HIV.
17. Active hepatitis B (defined as having a positive hepatitis B surface antigen (HBsAg) test at screening) or hepatitis C. Patients with past hepatitis B virus (HBV) infection or resolved HBV infection (defined as having a negative HBsAg test and a positive antibody to hepatitis B core antigen (anti-HBc) antibody test) are eligible. Patients positive for hepatitis C virus (HCV) antibody are eligible only if polymerase chain reaction (PCR) is negative for HCV RNA.
18. Active tuberculosis.
19. Currently receiving study therapy or has participated in a study of an investigational agent and received study therapy or used an investigational device within 4 weeks of the first dose of treatment.
20. Received treatment with immune checkpoint modulators, including anti-CTLA-4, anti-PD-1 or anti-PD-L1 therapeutic antibodies.
21. Received treatment with systemic immunostimulatory agents (including, but not limited, to interferons or IL-2) within 4 weeks or five half-lives of the drug (whichever is shorter) before randomization.
22. Received treatment with systemic corticosteroids or other systemic immunosuppressive medications (including, but not limited to, prednisone, dexamethasone, cyclophosphamide, azathioprine, methotrexate, thalidomide and anti-tumor necrosis factor (TNF) agents) within 2 weeks before randomization or anticipated requirement for systemic immunosuppressive medications during the trial.
  - Patients who have received acute, low-dose, systemic immunosuppressant medications (for example, a one-time dose of dexamethasone for nausea) may be enrolled in the study.
  - Patients with a history of allergic reaction to i.v. contrast requiring steroid pre-treatment should have baseline and subsequent tumor assessments performed using MRI.
  - The use of inhaled corticosteroids for chronic obstructive pulmonary disease, mineralocorticoids (for example, fludrocortisone) for patients with orthostatic hypotension and low-dose supplemental corticosteroids for adrenocortical insufficiency are allowed.
23. Received anti-cancer therapy (medical agents or radiation) within 1 week before study cycle 1, day 1.
24. A history or current evidence of any condition, therapy or laboratory abnormality that might confound the results of the trial, interfere with the patient’s participation for the full duration of the trial or is not in the best interest of the patient to participate, in the opinion of the treating investigator.
25. Known psychiatric or substance abuse disorders that would interfere with cooperation and the requirements of the trial.
26. Any reason why, in the opinion of the investigator, the patient should not participate. This includes a careful evaluation of whether standard therapy is preferable to the study therapy, for the individual patient.

### Randomization and masking

Participants were randomly assigned (2:3) to receive either placebo + chemotherapy (placebo-chemo) or atezolizumab + chemotherapy (atezo-chemo). Randomization was done by the investigators using an unstratified permuted block design through an interactive web response system implemented in the electronic case report form Viedoc v4 (Viedoc Technologies). Randomization listings were generated by an independent statistician at Clinical Trial Unit Research Support Services, Oslo University Hospital, using Stata 14 software (StataCorp). Atezolizumab and matching placebo had identical packaging, labeling, appearance and administration schedules. Participants, investigators and study site personnel (other than pharmacy personnel involved in placebo/drug preparation) were blinded to the treatment assignment until database lock.

### Procedures

The chemotherapy regimen was the same for both treatment groups and consisted of PLD (20 mg/m^2^ i.v. on day 1 of each 14-day cycle) and cyclophosphamide (50 mg by mouth (p.o.) daily in every other 14-day cycle). Atezolizumab (840 mg) or placebo was given i.v. on day 1 of each cycle. Treatment continued until disease progression, unacceptable toxicity, withdrawal of consent, investigator’s decision or up to 24 months. Treatment beyond 24 months was allowed for selected patients based on a risk/benefit analysis performed by the study management.

The baseline assessment included a medical history, a full clinical examination, a complete blood count (CBC), a comprehensive blood chemistry panel and a cardiac assessment by electrocardiogram and echocardiography. CBC and blood chemistry were repeated on day 1 of each treatment cycle, and a full clinical examination, electrocardiogram and echocardiography were repeated every 4th cycle. Patient-reported outcomes (PROs) were recorded on day 1 of cycles 1, 5, 9, 13 and 25 and at the time of treatment discontinuation, using the Chalder fatigue questionnaire (FQ), the European Organization for Research and Treatment of Cancer Quality of Life Questionnaire Core 15 Palliative Care (EORTC QLQ-C15-PAL) and the NRS pain scale. After consent, tumor tissue, blood, urine and feces samples were collected for a research biobank at several timepoints before, during and after trial treatment. Extraction of PBMCs was only done at the three Norwegian study sites.

Tumor evaluation by CT of the chest, abdomen and pelvis and a bone scan (MRI, PET or scintigraphy) was performed within 21 days of randomization and repeated every 8 weeks from the first day of treatment for the first 12 months and every 12 weeks thereafter. Response evaluation was done using iRECIST, a version of the RECIST modified to capture the response patterns of immunotherapies^25,26,36^. In patients with unconfirmed disease progression by iRECIST (iUPD), continued treatment was allowed until progression was confirmed (iCPD) in patients considered clinically stable according to the criteria in the iRECIST guideline.

Safety was monitored continuously throughout the study. All AEs were recorded and classified according to the Common Terminology Criteria for Adverse Events (CTCAE) version 4.0, regardless of relation to the study drugs. An independent safety monitoring committee periodically reviewed the study safety data.

### Endpoints

The co-primary endpoints were safety and a descriptive comparison of the PFS in the two arms. Safety was evaluated as the incidence, nature and severity of AEs. Efficacy was evaluated as time-to-event and by the proportion of patients without a PFS event 15 months after randomization. The secondary efficacy outcomes included OS, ORR, DoR, DRR and CBR. Owing to the limited sample size of this phase 2 trial, all comparisons of survival and response rates were of a descriptive nature. The exploratory outcomes included analysis of PFS in predefined subgroups defined by PD-L1 status, disease-free interval, line of treatment, metastatic sites, TMB, intrinsic breast cancer subtype and TIS^27^, as well as the assessment of immunological response, evaluation of potential biomarkers for clinical response, toxicity and immune response and developments in PROs (FQ, NRS pain intensity and EORTC QLQ-C15-PAL^37^). PD-L1 expression, mutation load and immune gene expression were predefined potential biomarkers. PFS was defined as the time from randomization to disease progression according to iRECIST, as assessed by the investigator, or death from any cause. OS was defined as the time from randomization to death from any cause. ORR was defined as the proportion of patients with a partial or complete response by iRECIST. DoR was defined as the time from the first documentation of an objective response to the time of progression or death, and DRR was defined as the proportion of patients with a DoR of ≥6 months. CBR was defined as the proportion of patients who had either an objective response or stable disease lasting at least until the radiological evaluation at 24 weeks ± 7 days.

### Statistical analysis

The primary efficacy hypothesis was that atezo-chemo would lead to prolonged PFS compared to placebo-chemo in mTNBC. The initial sample size was calculated based on a two-sided test with an alpha level of 10% and a power of 80% to detect an absolute reduction of 15% in the proportion without progression or death at 15 months in the atezo-chemo arm compared to the placebo-chemo arm. The estimated sample size was 75 patients (45 in atezo-chemo and 30 in placebo-chemo).

Safety was evaluated in the FAS, which included all patients who received one dose of any of the investigational medical products (IMPs). The primary efficacy analysis was done in the PP population and in the PD-L1^positive^ PP subpopulation. Secondary efficacy analyses were performed in the FAS, PP, PD-L1^positive^ PP and PD-L1^negative^ PP populations. For patients who discontinued the trial without progression or death, PFS was censored at the date of the last tumor assessment. Patients who could not be evaluated for tumor response were censored 1 day after randomization. Patients who were alive at the time of data lock were censored at the last timepoint at which they were confirmed to be alive.

Changes in the EORTC QLQ-C15 global health status/quality of life score were analyzed by time-to-deterioration and mean change from baseline with 95% CI. Deterioration was defined as a score reduction of ≥20 points from baseline. Patients with missing baseline values were not included in these analyses, and patients with no follow-up values were censored for deterioration on day 1. Patients who missed two consecutive assessments were censored for deterioration at the last assessment before this.

Comparisons of time-to-event outcomes between groups were made using the Kaplan–Meier method, with *P* values calculated using the log-rank method. The median follow-up time was calculated using the reverse Kaplan–Meier method, with censoring for OS as the event. HRs with 95% CIs were estimated using the Cox proportional hazards model. The Efron method was used for handling of tied event times. CIs for proportions were estimated by the Wilson score method. Circulating immune cell populations across different timepoints were compared by fold change, with *P* values calculated using Wilcoxon matched-pairs signed-rank test. All reported *P* values are two-sided.

Statistical analyses were done in R version 4.1.3 (ref. ^38^). Time-to-event analyses were performed using R packages survival (version 3.4) and survminer (version 0.4.9).

### PD-L1 scoring of tumors

The PD-L1 status of tumor samples was assessed by experienced pathologists who were blinded for treatment group and clinical outcome, according to *VENTANA PD-L1 (SP142) Assay Interpretation Guide for Triple-Negative Breast Carcinoma*^39^. Results were reported as the percentage of the tumor area made up of antibody-stained immune cells, and tumors were considered PD-L1^positive^ if this value was ≥1%.

### Assessment of TILs

Assessment of TILs was done by examination of hematoxylin and eosin (H&E)-stained sections of tumor biopsies collected during the study screening period by two experienced breast cancer pathologists, who were blinded for treatment group and clinical outcome. Scoring was performed based on the presence and abundance of lymphocytes within the borders of the invasive tumor. The scoring system included two categories for low infiltration (score 0–1) and two categories for high infiltration (score 2–3).

### Assessment of TMB

#### DNA extraction from tumor biopsies and blood genomic DNA

Fresh frozen tumor biopsies were quickly disrupted and homogenized using TissueLyzer (Qiagen). Subsequently, DNA/RNA/proteins were isolated from the tumor lysate using the AllPrep DNA/RNA/Protein Mini Kit (80004, Qiagen). Genomic DNA from blood was isolated using FlexiGene DNA Kit (51206, Qiagen). Each step was carried out as per the manufacturer’s instructions. DNA quality was measured by a NanoDrop spectrophotometer (Thermo Fisher Scientific), and the DNA concentration was determined by a Qubit fluorometer (Thermo Fisher Scientific) before further processing and analysis.

#### Whole-exome sequencing

The samples were further processed and sequenced by the Genomics Core Facility, Institute for Cancer Research, Oslo University Hospital. In brief, 50 ng of DNA was processed following the manufacturer’s recommendations for library preparation and target enrichment using Twist Comprehensive Human Exome and Twist Library Preparation EF Kit (Twist Bioscience). Subsequently, the samples were sequenced by paired-end sequencing 2×151 bp on the Illumina NovaSeq 6000 system.

#### Variant calling

Germline and somatic variant calling was performed by the Bioinformatics Core Facility, Institute for Cancer Research, Oslo University Hospital, and analyzed by the nf-core/Sarek 2.7 pipeline^40^. In brief, fastq files containing the sequencing raw data were aligned to the human GRCh38 reference genome by BWA-mem^41^. After sequence alignment, the duplicate reads were marked by GATK MarkDuplicatesSpark, followed by calibration of base qualities by GATK BaseRecalibrator and GATK ApplyBQSR. The depth of sequencing coverage was estimated on the recalibrated .bam files by GATK DepthOfCoverage 4.2.5.0 (ref. ^42^). Structural variants were detected by Manta^43^. Somatic variant calling was performed by Mutect2 (ref. ^42^) and Strelka 2 (ref. ^44^) to detect single-nucleotide variations and small insertions and deletions. Somatic variants detected in both variant callers were selected by BCFTools 1.9 (ref. ^45^) and used in further analysis.

#### TMB

TMB was calculated following the Uniform TMB estimation method proposed by Merino et al.^46^. In brief, the median sequencing depth of the tumor samples was >300×. Mutations were filtered before TMB calculation to include only coding, non-synonymous single-nucleotide polymorphisms and insertions and deletions. In addition, mutations with a tumor allele frequency ≥5%, tumor read depth ≥25 and a minimum of three supporting reads were included in the calculation. Samples were excluded from further analysis if more than 50% of variants were removed by the quality filters. Data from 53/68 patients in the FAS were available after quality control. The approximate size of the human exome (35.01 Mb) was used as a denominator in the TMB calculation. For patients with more than one sample, the highest TMB was considered representative.

### Gene expression analysis (NanoString)

The Research-use-only Human nCounter Breast Cancer 360 (BC360) panel identifies 776 genes of interest using optical barcodes consisting of capture and reporter probes that hybridize with mRNA targets. This enables digital counting of individual mRNA in each sample. The BC360 panel and kit were provided by NanoString Technologies.

Tumor mRNA isolation from 45 patients was performed using 10-µm FFPE tissue sections, mounted on histology slides and deparaffinized using (R)-(+)-Limonene (Merck, 183164) and rehydrated absolute ethanol and air-dried for a minimum of 15 minutes. Macrodissection was done with an H&E guide slide, collecting only tumor tissue from the sections. RNA isolation of the collected tumor tissue was done using the High Pure FFPET RNA Isolation Kit (Roche, 06650775001) and protocol, with the 55 °C incubation time increased to 2 hours. Hybridization of mRNA and capture and reporter probes was done using the nCounter XT Assay protocol (NanoString Technologies) and incubated at 65 °C for 20 hours. Post-hybridization processing was performed on the nCounter Prep Station (NanoString Technologies) at the ‘high sensitivity’ setting. The nCounter Digital Analyzer (NanoString Technologies) was used for digital counting and data collection with field of view (FOV) set to 555. Best practice for the BC360 assays was followed. Preliminary quality control was performed using the RCCCollector (NanoString Technologies) tool before further data processing. The determination of intrinsic breast cancer subtypes and calculation of TIS were done by NanoString Technologies. As the distribution and clinically relevant cutoff values are not established for TIS, the values were scaled to a mean of 0 and a standard deviation of 1 before presentation, to achieve numerical values that reflect the distribution of TIS in the study population.

### Flow cytometry analysis of peripheral blood immune cell subsets

PBMCs collected at screening and day 1 of the fifth treatment cycle (8 weeks after start of therapy) were isolated from whole blood using LymphoPrep Cell Separation Media (Abbott Rapid Diagnostics), frozen and stored in liquid nitrogen until assessed for immune cell populations by flow cytometry. Paired samples from each patient were stained and analyzed on the same day. The experiments were not replicated. PBMCs were initially incubated with antibodies for the surface markers CD3-BUV395, CD8-FITC, CD4-BV510, γδ-TCR-BUV737, CD19-BUV563, CD56-Alexa Fluor 647 (BD Biosciences), CD25-BV605 (BioLegend) and Fixable Viability Dye eFluor780 (Thermo Fisher Scientific). After fixation and permeabilization using eBioscience Foxp3/Transcription Factor Staining Buffer Set (Thermo Fisher Scientific), PBMCs were incubated with an antibody for the intracellular antigen Foxp3-PE (Thermo Fisher Scientific). Samples were acquired using BD FACSymphony A5 flow cytometer (BD Biosciences), and the data were analyzed with FlowJo (Tree Star) and GraphPad Prism (GraphPad Software).

### Role of the funding source

This study was supported by grants from the Norwegian Health Region South-East and the Norwegian Cancer Society/Norwegian Breast Cancer Society. Roche supported the study with free drug (atezolizumab), free SP142 kits and a funding contribution. NanoString supported the study with free assays and analyses. Roche provided critical reviews of the protocol. None of the funders had any role in the study design, data collection, data analysis, data interpretation or writing of the report. Roche and NanoString reviewed the first version of this manuscript.

### Reporting summary

Further information on research design is available in the Nature Portfolio Reporting Summary linked to this article.

## Online content

Any methods, additional references, Nature Portfolio reporting summaries, source data, extended data, supplementary information, acknowledgements, peer review information; details of author contributions and competing interests; and statements of data and code availability are available at 10.1038/s41591-022-02126-1.

### Supplementary information

Supplementary Information Supplementary Table 1, Supplementary Fig. 1 and Supplementary Protocol

Reporting Summary

## Figures and Tables

**Extended Data Table 1:**
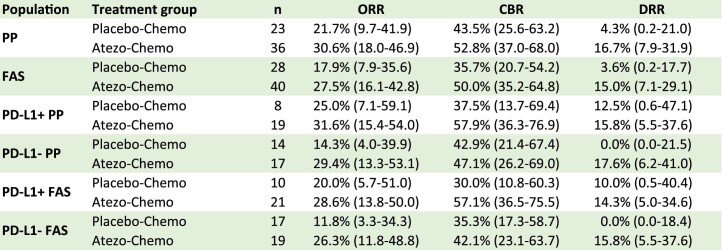
Summary of response variables. Proportions of patients with objective response, clinical benefit, and durable response (> 6 months) as assessed by iRECIST. Confidence intervals for proportions were calculated using the Wilson score method. ORR, objective response rate; CBR, clinical benefit rate; DRR, durable response rate; PP, per-protocol population; FAS, full analysis set; PD-L1, programmed death-ligand 1

**Extended Data Table 2:**
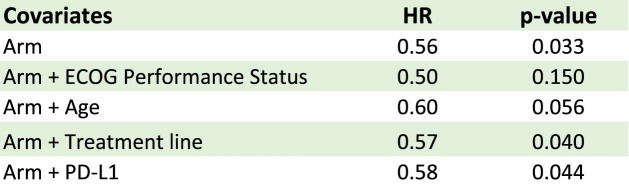
Multivariable Cox regression. The hazard ratio (HR) and associated p-value for progression-free survival in the atezo-chemo arm versus the placebo-chemo arm is shown unadjusted and after inclusion of selected baseline characteristics. Age is treated as a continuous variable and all other covariates as categorical. Hazard ratios and two-sided p-values were calculated using the Cox proportional hazards method. ECOG, Eastern Cooperative Oncology Group; PD-L1, programmed death-ligand 1

**Extended Data Table 3:**
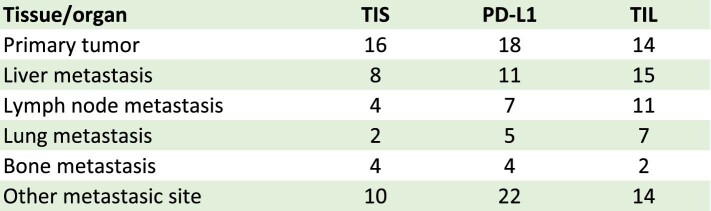
Biopsy sites. Biopsies used in the analysis of Tumor Inflammation Signature (TIS), programmed death-ligand 1 (PD-L1) expression and tumor-infiltrating lymphocytes (TIL), sorted by organ / tissue biopsied

**Extended Data Table 4:**
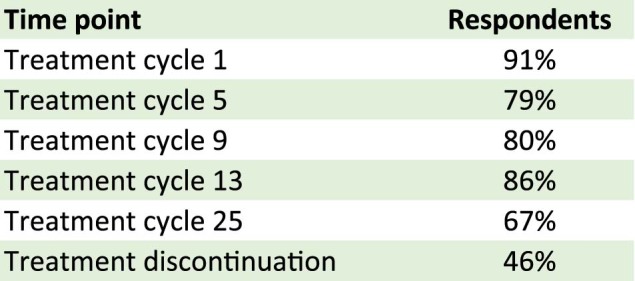
Proportion of patients responding to EORTC QLQ-C15-PAL The table shows the proportion of patients that responded to the quality of life item in the EORTC QLQ-C15-PAL questionnaire at each of the protocol-defined time points, as a percentage of all patients who remained in the study until that time point

## Data Availability

Any request for raw or analyzed data will be reviewed by the study team, and a response can be expected within 14 days. The data generated in this study are subject to patient confidentiality, and the transfer of data or materials will require approval from the Data Privacy Officer and the institutional review board at Oslo University Hospital and from the Regional Committee for Medical and Health Research Ethics South-East Norway and the Research Ethics Committee in Denmark. Any shared data will be de-identified. Requests should be made to the corresponding author (jonky@ous-hf.no).
