# Supplementary material for: Atezolizumab plus anthracycline-based chemotherapy in metastatic triple-negative breast cancer: the randomized, double-blind phase 2b ALICE trial
Source: Nat Med. 2022 Dec 8;28(12):2573–83. doi: 10.1038/s41591-022-02126-1 (PMC9800277; doi:10.1038/s41591-022-02126-1)
Supplement: Supplementary file 1 — Supplementary Table 1, Supplementary Fig. 1 and Supplementary Protocol [file 41591_2022_2126_MOESM1_ESM.pdf]

nature medicine

Article https://doi.org/10.1038/s41591-022-02126-1

# Atezolizumab plus anthracycline-based chemotherapy in metastatic triple-negative breast cancer: the randomized, double-blind phase 2b ALICE trial

In the format provided by the authors and unedited

## Supplementary Table 1 | Adverse events.

Complete list of adverse events, graded according to the Common Terminology Criteria for Adverse Events (CTCAE) version 4.0.

| | Placebo-Chemo (n = 28) | | | | Atezo-Chemo (n = 40) | | | |
|---|---|---|---|---|---|---|---|---|
| | All AE | | Treatment-related AE | | All AE | | Treatment-related AE | |
| AE term | Any grade | Grade 3-4 | Any grade | Grade 3-4 | Any grade | Grade 3-4 | Any grade | Grade 3-4 |
| Nausea | 15 | 0 | 13 | 0 | 23 | 2 | 21 | 1 |
| Rash | 11 | 0 | 9 | 0 | 26 | 7 | 26 | 6 |
| Fatigue | 12 | 0 | 12 | 0 | 20 | 2 | 20 | 2 |
| Constipation | 12 | 0 | 7 | 0 | 18 | 0 | 10 | 0 |
| Lymphocyte count decreased | 10 | 5 | 10 | 5 | 18 | 6 | 18 | 6 |
| Mucosal inflammation | 6 | 0 | 4 | 0 | 19 | 0 | 19 | 0 |
| Palmar-plantar erythrodysaesthesia syndrome | 3 | 1 | 3 | 1 | 21 | 3 | 21 | 3 |
| Musculoskeletal pain | 6 | 0 | 1 | 0 | 12 | 2 | 6 | 1 |
| Dyspepsia | 3 | 0 | 1 | 0 | 8 | 0 | 5 | 0 |
| Decreased appetite | 3 | 0 | 3 | 0 | 7 | 1 | 7 | 1 |
| Dyspnoea | 4 | 0 | 2 | 0 | 6 | 2 | 1 | 1 |
| Diarrhoea | 6 | 0 | 6 | 0 | 4 | 0 | 2 | 0 |
| Urinary tract infection | 1 | 0 | 0 | 0 | 8 | 2 | 6 | 2 |
| Pruritus | 2 | 0 | 1 | 0 | 7 | 0 | 6 | 0 |
| Skin infection | 2 | 0 | 1 | 0 | 7 | 0 | 5 | 0 |
| Cough | 4 | 0 | 3 | 0 | 5 | 0 | 2 | 0 |
| Headache | 5 | 1 | 3 | 0 | 4 | 0 | 4 | 0 |
| Upper respiratory tract infection | 0 | 0 | 0 | 0 | 8 | 2 | 7 | 1 |
| Dry mouth | 2 | 0 | 1 | 0 | 6 | 0 | 6 | 0 |
| Abdominal pain | 3 | 0 | 1 | 0 | 5 | 0 | 3 | 0 |
| Neutrophil count decreased | 4 | 2 | 4 | 2 | 4 | 1 | 4 | 1 |
| Hypothyroidism | 2 | 0 | 1 | 0 | 5 | 0 | 5 | 0 |
| Pleural effusion | 1 | 0 | 0 | 0 | 5 | 3 | 0 | 0 |
| Pyrexia | 2 | 0 | 1 | 0 | 4 | 1 | 2 | 1 |
| Pneumonitis | 1 | 0 | 1 | 0 | 4 | 2 | 4 | 2 |
| Dizziness | 1 | 0 | 0 | 0 | 4 | 0 | 3 | 0 |
| Infusion related reaction | 1 | 0 | 1 | 0 | 4 | 0 | 4 | 0 |
| Neuropathy peripheral | 3 | 0 | 1 | 0 | 2 | 0 | 2 | 0 |
| Vulvovaginal dryness | 0 | 0 | 0 | 0 | 4 | 1 | 4 | 1 |
| Oral fungal infection | 1 | 0 | 1 | 0 | 3 | 0 | 1 | 0 |
| Oedema | 2 | 1 | 2 | 1 | 2 | 0 | 1 | 0 |
| Lung infection | 0 | 0 | 0 | 0 | 3 | 1 | 3 | 1 |
| Hypersensitivity | 0 | 0 | 0 | 0 | 3 | 0 | 2 | 0 |
| Weight decreased | 0 | 0 | 0 | 0 | 3 | 0 | 3 | 0 |
| Infection | 1 | 1 | 1 | 1 | 2 | 1 | 2 | 1 |
| Thrombosis | 1 | 0 | 0 | 0 | 2 | 1 | 0 | 0 |
| Hypokalaemia | 2 | 1 | 1 | 1 | 1 | 0 | 1 | 0 |
| Hyperthyroidism | 2 | 0 | 2 | 0 | 1 | 0 | 1 | 0 |
| Corona virus infection | 0 | 0 | 0 | 0 | 2 | 0 | 0 | 0 |
| Dry eye | 0 | 0 | 0 | 0 | 2 | 0 | 2 | 0 |
| Dysgeusia | 0 | 0 | 0 | 0 | 2 | 0 | 2 | 0 |
| Dysphagia | 0 | 0 | 0 | 0 | 2 | 0 | 2 | 0 |
| Fracture | 0 | 0 | 0 | 0 | 2 | 1 | 0 | 0 |
| Gastroenteritis | 0 | 0 | 0 | 0 | 2 | 0 | 2 | 0 |
| Hyperglycaemia | 0 | 0 | 0 | 0 | 2 | 0 | 1 | 0 |
| Malaise | 0 | 0 | 0 | 0 | 2 | 0 | 1 | 0 |
| Pain | 0 | 0 | 0 | 0 | 2 | 0 | 2 | 0 |
| Parosmia | 0 | 0 | 0 | 0 | 2 | 0 | 1 | 0 |
| Pneumothorax | 0 | 0 | 0 | 0 | 2 | 1 | 0 | 0 |
| Vomiting | 0 | 0 | 0 | 0 | 2 | 0 | 1 | 0 |
| Pancreatic enzymes increased | 1 | 1 | 1 | 1 | 1 | 1 | 1 | 1 |
| Pulmonary embolism | 1 | 1 | 0 | 0 | 1 | 1 | 1 | 1 |
| Syncope | 1 | 1 | 1 | 1 | 1 | 1 | 0 | 0 |
| Anaemia | 1 | 0 | 1 | 0 | 1 | 0 | 1 | 0 |
| Cystitis noninfective | 1 | 0 | 1 | 0 | 1 | 0 | 1 | 0 |
| Hepatic enzyme increased | 1 | 0 | 1 | 0 | 1 | 0 | 1 | 0 |
| Mastitis | 1 | 0 | 1 | 0 | 1 | 0 | 0 | 0 |
| Skin ulcer | 1 | 0 | 1 | 0 | 1 | 0 | 1 | 0 |
| Cholecystitis | 0 | 0 | 0 | 0 | 1 | 1 | 1 | 1 |
| Hypophosphataemia | 0 | 0 | 0 | 0 | 1 | 1 | 1 | 1 |
| Pancreatitis | 0 | 0 | 0 | 0 | 1 | 1 | 1 | 1 |
| Tenosynovitis | 0 | 0 | 0 | 0 | 1 | 1 | 1 | 1 |
| Acute kidney injury | 0 | 0 | 0 | 0 | 1 | 0 | 0 | 0 |
| Alopecia | 0 | 0 | 0 | 0 | 1 | 0 | 1 | 0 |
| Breast pain | 0 | 0 | 0 | 0 | 1 | 0 | 0 | 0 |
| Chills | 0 | 0 | 0 | 0 | 1 | 0 | 1 | 0 |
| Dermal cyst | 0 | 0 | 0 | 0 | 1 | 0 | 1 | 0 |
| Dysphonia | 0 | 0 | 0 | 0 | 1 | 0 | 0 | 0 |
| Ejection fraction decreased | 0 | 0 | 0 | 0 | 1 | 0 | 1 | 0 |

| | | | | | | | | |
|---|---|---|---|---|---|---|---|---|
| Emphysema | 0 | 0 | 0 | 0 | 1 | 0 | 1 | 0 |
| Facet joint syndrome | 0 | 0 | 0 | 0 | 1 | 0 | 0 | 0 |
| Flatulence | 0 | 0 | 0 | 0 | 1 | 0 | 0 | 0 |
| Haemorrhoids | 0 | 0 | 0 | 0 | 1 | 0 | 0 | 0 |
| Hepatotoxicity | 0 | 0 | 0 | 0 | 1 | 0 | 0 | 0 |
| Hot flush | 0 | 0 | 0 | 0 | 1 | 0 | 0 | 0 |
| Hyperkalaemia | 0 | 0 | 0 | 0 | 1 | 0 | 0 | 0 |
| Menstruation irregular | 0 | 0 | 0 | 0 | 1 | 0 | 1 | 0 |
| Onychomadesis | 0 | 0 | 0 | 0 | 1 | 0 | 1 | 0 |
| Oral dysaesthesia | 0 | 0 | 0 | 0 | 1 | 0 | 1 | 0 |
| Paronychia | 0 | 0 | 0 | 0 | 1 | 0 | 0 | 0 |
| Periodontitis | 0 | 0 | 0 | 0 | 1 | 0 | 1 | 0 |
| Plantar fasciitis | 0 | 0 | 0 | 0 | 1 | 0 | 0 | 0 |
| Skin reaction | 0 | 0 | 0 | 0 | 1 | 0 | 0 | 0 |
| Skin wound | 0 | 0 | 0 | 0 | 1 | 0 | 1 | 0 |
| Throat tightness | 0 | 0 | 0 | 0 | 1 | 0 | 1 | 0 |
| Toothache | 0 | 0 | 0 | 0 | 1 | 0 | 1 | 0 |
| Urinary tract obstruction | 0 | 0 | 0 | 0 | 1 | 1 | 0 | 0 |
| Vertigo | 0 | 0 | 0 | 0 | 1 | 0 | 0 | 0 |
| Vulvitis | 0 | 0 | 0 | 0 | 1 | 0 | 1 | 0 |
| Atrioventricular block complete | 1 | 1 | 1 | 1 | 0 | 0 | 0 | 0 |
| Amnesia | 1 | 0 | 0 | 0 | 0 | 0 | 0 | 0 |
| Conjunctivitis | 1 | 0 | 1 | 0 | 0 | 0 | 0 | 0 |
| Cushingoid | 1 | 0 | 0 | 0 | 0 | 0 | 0 | 0 |
| Dysarthria | 1 | 0 | 0 | 0 | 0 | 0 | 0 | 0 |
| Hair growth abnormal | 1 | 0 | 0 | 0 | 0 | 0 | 0 | 0 |
| Hepatic haemorrhage | 1 | 0 | 0 | 0 | 0 | 0 | 0 | 0 |
| Hepatic pain | 1 | 1 | 0 | 0 | 0 | 0 | 0 | 0 |
| Hypotension | 1 | 0 | 0 | 0 | 0 | 0 | 0 | 0 |
| Insomnia | 1 | 0 | 0 | 0 | 0 | 0 | 0 | 0 |
| Leukopenia | 1 | 0 | 0 | 0 | 0 | 0 | 0 | 0 |
| Nail ridging | 1 | 0 | 1 | 0 | 0 | 0 | 0 | 0 |
| Painful respiration | 1 | 0 | 0 | 0 | 0 | 0 | 0 | 0 |
| Pigmentation disorder | 1 | 0 | 1 | 0 | 0 | 0 | 0 | 0 |
| Skin hyperpigmentation | 1 | 0 | 1 | 0 | 0 | 0 | 0 | 0 |
| Thirst | 1 | 0 | 0 | 0 | 0 | 0 | 0 | 0 |
| Vaginal haemorrhage | 1 | 0 | 0 | 0 | 0 | 0 | 0 | 0 |
| Vulvovaginal pain | 1 | 0 | 1 | 0 | 0 | 0 | 0 | 0 |

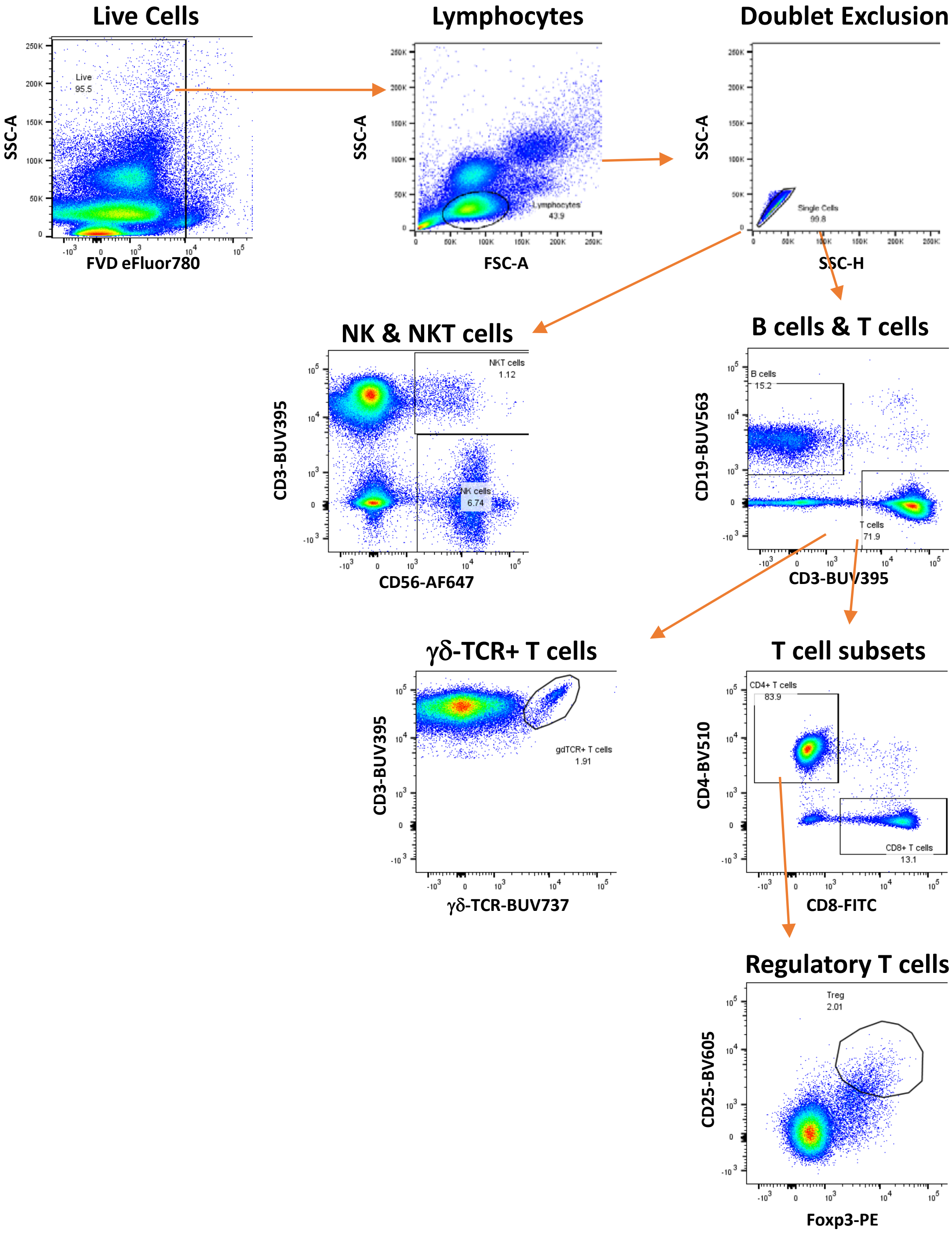


**Supplementary Figure 1 | Flow cytometry gating strategy for *ex vivo* PBMC phenotyping**

A. Pseudocolor dot plots of PBMC from a representative patient showing the gating strategy used to identify the different immune cell populations by flow cytometry. Immune cell subsets are defined as follows: CD4+ T cells ($CD3^+CD4^+CD8^-$), CD8+ T cells ($CD3^+CD4^-CD8^+$), Regulatory T cells ($CD3^+CD4^+Foxp3^+CD25^{Hi}$), B cells ($CD3^-CD19^+$), NK cells ($CD3^-CD56^+$), NKT cells ($CD3^+CD56^+$) and γδ-T cells ($CD3^+$γδ-$TCR^+$). CD25 and Foxp3 positive populations were defined using Fluorescence minus one (FMO) to identify the negative populations.

**TITLE:**

# ALICE: A randomized placebo-controlled phase II study evaluating atezolizumab combined with immunogenic chemotherapy in patients with metastatic triple-negative breast cancer

**SPONSOR:**

**Oslo University Hospital**
**The Norwegian Radium Hospital**
**Department of Oncology**
**Ullernchausseen 70**
**0379 Oslo**
**Norway**

**PROTOCOL ML39079_ALICE**

| | |
|---|---|
| **EudraCT number:** | **2016-003570-40** |
| **Protocol version no.:** | **4.0** |
| **Protocol date:** | **17 December 2021** |

## CONTACT DETAILS


| | |
|---|---|
| **Sponsor:** | **Stein Kaasa, MD PhD**<br>Department of Oncology<br>Oslo University Hospital<br>Ph: +47 2293 4000 |
| **Principal Investigator:** | **Jon Amund Kyte, MD PhD**<br>Department of Oncology<br>Oslo University Hospital<br>Ph: +47 2293 4000/ +47 975 696 19 |
| **Co-Investigator:** | **Bjørn Naume, MD PhD, professor**<br>Department of Oncology<br>Oslo University Hospital<br>Ph: +47 2293 4000 |
| **Statisticians:** | **Ole Christian Lingjærde, PhD, professor**<br>Department of Computer Science<br>Faculty of Mathematics and Natural Sciences<br>University of Oslo<br>Ph: +47 466 36 659<br><br>**Ragnhild Sørum Falk, PhD**<br>Oslo Center for Biostatistics and Epidemiology<br>Oslo University Hospital<br>Ph: +47 986 88 111 |

# SIGNATURE PAGE

| | |
|---|---|
| Title | ALICE: A randomized placebo-controlled phase II study evaluating atezolizumab combined with immunogenic chemotherapy in patients with metastatic triple-negative breast cancer |
| Protocol ID no: | ALICE |
| EudraCT no: | 2016-003570-40 |

*I hereby declare that I will conduct the study in compliance with the Protocol, ICH GCP and the applicable regulatory requirements:*

| Name | Title | Role | Signature | Date |
|---|---|---|---|---|
| Stein Kaasa | Professor, MD PhD, Head of Department | Sponsors representative | | |
| Jon Amund Kyte | MD PhD, Senior consultant | Principal and coordinating investigator | | |
| | | | | |
| | | | | |

# TABLE OF CONTENTS

**Contact details** .......... **2**

**1.0 BACKGROUND & RATIONALE** .......... **8**

**2.0 OBJECTIVES AND HYPOTHESES** .......... **9**

**3.0 STUDY DESIGN** .......... **10**

3.1 Description of study .......... 10

3.2 Safety Monitoring Committee .......... 11

**4.0 INCLUSION CRITERIA** .......... **11**

**5.0 EXCLUSION CRITERIA** .......... **13**

**6.0 STUDY TREATMENT** .......... **16**

6.1 Overview of Study Treatment .......... 16

6.2 Rationale for chemotherapy regimen .......... 16

6.3 Rationale for Atezolizumab Dosage .......... 17

6.4 Risk benefit assessment .......... 18

6.5 Administration of Atezolizumab/Placebo .......... 18

6.6 Administration of pegylated liposomal doxorubicin .......... 20

6.7 Administration and compliance registration for cyclophosphamide .......... 21

**7.0 OUTCOME MEASURES** .......... **21**

7.1 Safety Outcome Measures .......... 21

7.2 Efficacy Outcome Measures .......... 21

**8.0 TRIAL FLOW CHART** .......... **22**

8.1 Study Flow Chart .......... 22

**9.0 TRIAL PROCEDURES** .......... **26**

9.1 Trial Procedures .......... 26

9.1.1 Administrative Procedures .......... 26

9.1.2 Clinical Procedures/Assessments ........ 28

9.1.3 Withdrawal/Discontinuation ........ 31

9.1.4 Post-Treatment Visits ........ 31

9.1.5 Trial handling during the SARS-CoV-2 situation ........ 32

**10.0 SAFETY ASSESSMENT ........ 33**

10.1 Safety plan ........ 33

10.1.1 Risks Associated with Atezolizumab ........ 33

10.1.2 Eligibility Criteria ........ 34

10.1.3 Monitoring ........ 34

10.1.4 Dose Modification ........ 35

10.1.5 Atezolizumab/Placebo Dose Modification ........ 36

10.1.6 Management of Atezolizumab/Placebo-Specific Adverse Events ........ 36

10.1.7 Dose-modification of chemotherapy ........ 37

10.2 Safety parameters and definitions ........ 38

10.2.1 Adverse Events ........ 38

10.2.2 Serious Adverse Events (Immediately Reportable to the Sponsor) ........ 38

10.2.3 Suspected Unexpected Serious Adverse Reactions (Immediately Reportable to the Sponsor) ........ 39

10.2.4 Adverse Events of Special Interest (Immediately Reportable to the Sponsor)... 39

10.2.5 Procedures for recording of adverse events ........ 40

10.3 Immediate reporting requirements from investigator to sponsor ........ 42

10.3.1 Reporting Requirements for Serious Adverse Events and Non-Serious Adverse Events of Special Interest ........ 42

10.3.2 Reporting Requirements for Pregnancies ........ 43

10.4 Follow-up of patients after adverse events ........ 44

10.4.1 Investigator Follow-Up ........ 44

10.4.2 Sponsor Follow-Up ........ 44
10.5 Post-study adverse events ........ 44
10.6 Sponsor Responsibility for Reporting Adverse Events ........ 44
**11.0 COLLATERAL RESEARCH ........ 45**
11.1 Biobanking ........ 45
11.2 Plans for translational research addressing exploratory/secondary endpoints ........ 46
**12.0 STATISTICS ........ 46**
12.1 Statistical analyses ........ 46
12.2 Statistical considerations regarding sample size and randomization ratio ........ 48
**13.0 Ehical considerations ........ 49**
13.1 Compliance with Laws and Regulations ........ 49
13.2 Informed Consent ........ 49
13.3 Ethics Committee ........ 50
13.4 Confidentiality ........ 50
**14.0 ADMINISTRATIVE AND REGULATORY DETAILS ........ 51**
14.1 Study Documentation ........ 51
14.2 Protocol Adherence ........ 51
14.3 Monitoring ........ 51
14.4 Audit and Inspections ........ 51
14.5 Data Management and Transfer ........ 51
14.6 Publication Policy ........ 52
**15.0 LIST OF REFERENCES ........ 53**
**16.0 Abbreviations ........ 55**
**17.0 APPENDICES ........ 57**
17.1 ECOG Performance Status ........ 57

17.2 Common Terminology Criteria for Adverse Events v 4.0 .................................... 57

17.3 Immune-modified Response Evaluation Criteria in Solid Tumors (iRECIST)...... 58

17.4 Response Evaluation Criteria in Solid Tumors (RECIST) .................................... 60

17.4.1 Measurability of tumor at baseline .................................................................... 60

17.4.2 Target lesions: specifications by methods of measurements .............................. 61

17.4.3 Tumor response evaluation .................................................................................. 62

17.5 EORTC QLQ-C15-PAL (version 1)........................................................................ 70

17.6 Chalder Fatigue Questionnaire (FQ) and pain score ............................................. 71

## 1.0 BACKGROUND & RATIONALE

Breast cancer is rarely curable after metastasis, and the therapeutic options for metastatic TNBC are limited. Interestingly, the host immune response is strongly predictive for the effect of chemotherapy in patients with TNBC(1). In the present proposal, we aim at releasing the brake on the immune response by the use of atezolizumab, an inhibitory antibody against Programmed Death Ligand-1 (PD-L1). Patients with a high number of tumor mutations are considered more likely to respond to PD1/PD-L1-blockade(2). Patients with TNBC have high mutation frequencies, compared to other forms of breast cancer

We hypothesize that atezolizumab may

i) Potentiate the patient´s spontaneous anti-tumor immune response
ii) Synergize with chemotherapeutic agents countering tumor tolerance and inducing immunological cell death.

There is compelling evidence from animal studies, supported by data from humans, that some chemotherapeutic agents are immunogenic. Doxorubicin and cyclophosphamide have been shown to be particularly powerful inducers of immunogenic cell death. Both agents fulfill 5/5 criteria established for assessing the immunogenicity of different chemotherapeutic drugs (Table 1 in (3)). There is also strong evidence from humans, particularly in breast cancer, indicating that the clinical effect of doxorubicin and cyclophosphamide depends on the host immune response (4). Further, these agents have been shown to induce a Type I interferon immune response in breast cancer (5, 6). Taken together, there is a strong rationale for synergy between doxorubicin/cyclophosphamide and PD-L1 blockade (7).

We will combine atezolizumab with established chemotherapy in patients with metastatic TNBC. The prospect of clinical benefit from PD-L1-blockade is probably best in patients that have not received multiple previous lines of chemotherapy, and we thus aim to bring atezolizumab into current early line regimens. Our chemotherapeutic regimen is a combination of anthracycline and cyclophosphamide, which is an acknowledged and widely used option. To facilitate rapid recruitment into the study, we will allow for up to one previous line of chemotherapy, with a requirement of good performance status (ECOG 0 or 1) and adequate organ function. Further, we suggest using the drugs in a semi-metronomic fashion (pegylated liposomal doxorubicin every 2$^{nd}$ week and daily cyclophosphamide), rather than as high dose regimens administered every third/fourth week. We hypothesize that the semi-metronomic regimen will induce immunological cell death and counter T regulatory cells (8), while maintaining the leukocyte counts and the ability of the effector immune cells to respond. Indeed, a daily metronomic cyclophosphamide regimen has been used in several cancer vaccine studies, in order to counter regulatory T cells and myeloid suppressor cells. Finally, we will use pegylated liposomal doxorubicin (PLD; Caelyx), which minimizes the adverse effects of anthracyclines on the heart and allows for continued treatment beyond the otherwise mandatory anthracycline limits. This is of particular importance for immunotherapy, where the aim is to induce long term disease remission. It is important to identify a chemo regimen that can be continued for an extended period of time, in combination with atezolizumab.

In 2019, immunotherapy was for the first time approved by the FDA and EMA for BC, based on the IMpassion130 trial combining atezolizumab with chemotherapy against metastatic TNBC(9). This was the first randomized study demonstrating efficacy of immunotherapy against TNBC. An effect was only found in patients with evidence of immune activation up front, in the form of PD-L1 expression in ≥1% immune cells, using the Ventana SP142 assay. In the ALICE trial, we aim at triggering sensitivity to checkpoint blockade in patients that are otherwise not responsive, by use of "immunogenic chemotherapy", as outlined above. Data from recent trials suggest that anthracyclines may be superior to taxanes for this purpose, though no conclusion can yet be drawn. The TONIC trial addressed the issue of which chemotherapy to combine with PD1-blockade. Here, doxorubicin recorded the highest response rate when combined with aPD1. There was also biological evidence of immune activation in the tumor samples obtained after doxorubicin treatment, in line with the concept of immunogenic cell death. In the neoadjuvant setting, Keynote-522 showed significantly increased pathological complete response (pCR) rates for the group receiving aPD1, when combined with chemotherapy. In contrast, the NEOTRIP trial did not show a statistically significant pCR (a secondary endpoint, for which the trial was not designed nor powered) benefit for the addition of atezolizumab to chemotherapy. The chemotherapy backbone in Keynote 522 contained anthracyclines and cyclophosphamide, while the in NEOTRIP the chemotherapy backbone included only taxanes and platinum salts.

## 2.0 OBJECTIVES AND HYPOTHESES

**Primary objectives:**

1) Assessment of toxicity of combined treatment with atezolizumab, pegylated liposomal doxorubicin and cyclophosphamide
2) Assessment of clinical response: Progression-free survival; descriptive comparison of the PFS rates in the total per protocol (PP) population, and the PD-L1+ PP population

**Secondary objectives:**

3) Assessment of clinical response: Objective tumor response rate (ORR), duration of response (DR), durable tumor response rate (DRR; >6 months), clinical benefit rate (CBR), overall survival (OS)
4) Assessment of changes in the immunological milieu in tumor and peripheral blood after study therapy, considering each study arm separately, and by comparing arm A to arm B
5) Assessment of PD-L1 expression, mutation load and immune gene expression as biomarkers for clinical response
6) Assessment of patient reported outcomes, as measured by the Chalder Fatigue Questionnaire (FQ), an 11 point Numerical Rating Scale (NRS) for pain intensity and EORTC QLQ-C15-PAL

**Exploratory objectives:**

7) Assessment of immunological response

8) Identification of novel and integrated biomarkers for clinical response, toxicity and immune response
9) Characterization of tumor evolution induced by the study therapy, considering each study arm separately, and by comparing arm A to arm B
10) Characterization of changes in microbiota induced by the study therapy, considering each study arm separately, and by comparing arm A to arm B

**Hypotheses:**

1) The chemo/atezolizumab (c/a) combination has acceptable safety and tolerability in mTNBC
2) The c/a combination group will experience prolonged PFS compared to the control group
3) The c/a combination group will experience favorable ORR, DR, DRR, CBR and OS compared to the control group
4) The study therapy may trigger an immune reaction in the tumor, counter immunosuppressive cells and induce other changes in the systemic immune milieu
5) A high PD-L1 expression, mutation load and immune gene expression are predictive of clinical response.
6) The c/a combination will give improved pain control and reduced fatigue compared to chemo alone
7) The c/a combination will induce T cell responses against neoantigens and other antigens expressed in each patient´s tumor
8) Analysis of pre-treatment and under-treatment samples may identify novel biomarkers for predicting which patients will benefit from the chemo/atezolizumab combination
9) Profiling of consecutive samples obtained before/during/after treatment may uncover tumor evolution induced by the study therapy
10) Profiling of consecutive fecal samples obtained before/during/after treatment may uncover changes in microbiota induced by the study therapy

## 3.0 STUDY DESIGN

### 3.1 Description of study

This is a randomized, double-blind, placebo-controlled exploratory phase II study evaluating the safety and efficacy of atezolizumab when combined with immunogenic chemotherapy in subjects with metastatic triple-negative breast cancer. Atezolizumab, pegylated liposomal doxorubicin and cyclophosphamide are the Investigational Medicinal Products (IMPs).

- Randomized phase II trial in 75 patients comparing two arms:
    - Arm A: Chemo (pegylated liposomal doxorubicin + cyclophosphamide) + placebo
    - Arm B: Chemo (pegylated liposomal doxorubicin + cyclophosphamide) + atezolizumab
- Randomization 2:3 in favor of arm B

Upon radiographic disease progression per iRECIST, the patients must stop study treatment.

Patients that cannot be evaluated for tumor response or receive ≤3 doses of atezolizumab/placebo or ≤ 2 doses with PLD will not be considered evaluable per protocol, but will be included in the

intention to treat (ITT) analysis. To include a sufficient number of patients in the per protocol (PP) analysis, the same number of patients will be added to study. The new subjects will be randomized according to the initial 3:2 ratio.

In 2020, atezolizumab was introduced in the study countries (Norway and Denmark) as standard therapy for PD-L1 positive mTNBC, in the first line metastatic setting. As a result, from May 2020 only patients with PD-L1 negative disease are likely to be available for enrolment into the ALICE trial. The protocol has therefore been revised, so that an analysis of the PD-L1 negative group will be performed. If this analysis does not show a signal of possible benefit for patients with PD-L1 negative TNBC, the enrolment of new patients will be stopped (see paragraph 12.1).

### 3.2 Safety Monitoring Committee

A safety monitoring committee (SMC) will monitor safety on a periodic basis. Members of the SMC will be experienced clinicians at the Sponsor, independent from the study. The SMC will meet the first time when three patients have received three injections of atezolizumab/placebo, but no later than 6 months after first patient in (FPI). Thereafter the SMC will meet approximately every 6 months to review safety and study conduct data. The safety data will include demographic data, adverse events and relevant laboratory data.

Following each data review, the SMC will provide recommendations to the PI as to whether the study should continue or be amended, or whether the study should be stopped on the basis of safety (i.e., evidence of harm). The Study Leadership will make a decision on the basis of the SMC recommendations.

Any outcomes of these safety reviews that affect study conduct will be communicated in a timely manner to the investigators for notification of the Institutional Review Boards/Ethics Committees (IRBs/ECs).

## 4.0 INCLUSION CRITERIA

Patients must meet all of the following criteria to be eligible for study entry:

1. Metastatic or incurable locally advanced, histologically documented TNBC (negative for HER2, ER and PR). HER2 negativity is defined as either of the following by local laboratory assessment: In situ hybridization (ISH) non-amplified (ratio of HER2 to CEP17 < 2.0 or single probe average HER2 gene copy number < 4 signals/cell), or IHC 0 or IHC 1+ (if more than one test result is available and not all results meet the inclusion criterion definition, all results should be discussed with the PI to establish eligibility of the patient). ER and PR negativity are defined as < 1% and <10%, respectively, of cells expressing hormonal receptors via IHC analysis
2. Adequate newly obtained core or excisional biopsy of a tumor lesion not previously irradiated. No anti-tumor treatment is allowed between the time point for biopsy and study entry. If a patient has undergone chemotherapy in the metastatic setting, a new biopsy must be obtained after this therapy

3. Measurable disease according to iRECIST
4. Signed Informed Consent Form
5. Women or men aged ≥ 18 years
6. Eastern Cooperative Oncology Group (ECOG) performance status of 0 or 1
7. In patients that have received (neo)adjuvant treatment with anthracyclines or cyclophosphamide, a minimum of 12 months from treatment with anthracyclines or cyclophosphamide until relapse of disease is required
8. A maximum of one previous line with chemotherapy in the metastatic setting
9. Female subject of childbearing potential should have a negative urine or serum pregnancy test within 7 days prior to receiving the first dose of study medication. If the urine test is positive or cannot be confirmed as negative, a serum pregnancy test will be required
10. Female subjects of childbearing potential should agree to remain abstinent (refrain from heterosexual intercourse) or use contraceptive methods that result in a failure rate of < 1% per year, during the treatment period and for at least 5 months after the last dose of study therapy. A woman is considered to be of childbearing potential if she is postmenarcheal, has not reached a postmenopausal state (≥ 12 continuous months of amenorrhea with no identified cause other than menopause), and has not undergone surgical sterilization (removal of ovaries and/or uterus). Examples of contraceptive methods with a failure rate of < 1% per year include bilateral tubal ligation, male sterilization, proper use of hormonal contraceptives that inhibit ovulation and hormone-releasing intrauterine devices (IUDs). Periodic abstinence (e.g., calendar, ovulation, symptothermal, or postovulation methods) and withdrawal are not acceptable methods of contraception
11. Male subjects should agree to use an adequate method of contraception starting with the first dose of study therapy through 3 months after the last dose of study therapy
12. Able to swallow orally administrated medication.
13. Adequate organ function as defined in Table 1

Table 1 Adequate Organ Function Laboratory Values

| **System** | **Laboratory Value** |
|---|---|
| **Hematological** | |
| Absolute neutrophil count (ANC) | ≥1.20 x$10^9$/L |
| Lymphocyte count | ≥0.50 x$10^9$/L |
| Platelets | ≥80 x$10^9$/L |
| Hemoglobin | ≥9 g/dL or ≥5.6 mmol/L without transfusion or EPO dependency (within 7 days of assessment) |
| **Renal** | |
| Serum creatinine **OR**<br>Measured or calculated[a] creatinine clearance<br>(GFR can also be used in place of creatinine or CrCl) | ≤1.5 X upper limit of normal (ULN) **OR**<br>≥40 mL/min for subject with creatinine levels > 1.5 X institutional ULN |
| **Hepatic** | |
| Serum total bilirubin | ≤ 1.5 X ULN **OR**<br>Direct bilirubin ≤ ULN for subjects with total bilirubin levels > 1.5 ULN |

| | |
|---|---|
| AST (SGOT) and ALT (SGPT) | ≤ 2.5 X ULN **OR**<br>≤ 5 X ULN for subjects with liver metastases |
| Albumin | ≥2.5 g/dL |
| **Coagulation** | |
| International Normalized Ratio (INR) or Prothrombin Time (PT) | ≤1.5 X ULN unless subject is receiving anticoagulant therapy as long as PT or PTT is within therapeutic range of intended use of anticoagulants |
| [a] Creatinine clearance should be calculated per institutional standard. | |

## 5.0 EXCLUSION CRITERIA

The subject must be excluded from participating in the trial if the subject has/is:

1. Malignancies other than breast cancer within 5 years prior to randomization, with the exception of those with a negligible risk of metastasis or death and treated with expected curative outcome (such as adequately treated carcinoma in situ of the cervix or basal or squamous cell skin cancer)
2. Patients with known PD-L1 positive TNBC, as assessed by the Ventana SP142 assay (IC ≥1%), and no previous chemotherapy in the metastatic setting, should be offered standard therapy with nab-paclitaxel/atezolizumab outside of the trial, if they had a disease free interval of >12 months after previous (neo) adjuvant chemotherapy, unless the patient for other reasons should not receive nab-paclitaxel, according to own preferences, drug availability or recommendations by the treating physician. A history of progression on taxanes in the neoadjuvant setting, or severe side effects from taxane therapy, may represent sufficient reason to offer the patient inclusion into the ALICE-trial, if the physician considers that the patient should receive anthracyclines rather than taxanes as 1st line therapy for metastatic disease. If more than one TNBC biopsy has been evaluated for PD-L1 by the SP142 assay, and the results differ, the patient's PD-L1 status determination will be based on best clinical judgment.
3. Spinal cord compression not definitively treated with surgery and/or radiation, or previously diagnosed and treated spinal cord compression without evidence that disease has been clinically stable for > 2 weeks prior to randomization
4. Known CNS disease, except for asymptomatic CNS metastases, provided all of the following criteria are met:
    a. Measurable disease outside the CNS
    b. No metastases to mesencephalon, pons, medulla oblongata, or spinal cord
    c. No ongoing requirement for dexamethasone as therapy for CNS disease
    d. No radiation of brain lesions within 7 days prior to randomization
    e. No leptomeningeal disease
    f. Patients with symptomatic CNS metastases must receive radiation therapy and/or surgery for CNS metastases. Following treatment, these patients may be eligible, if all other criteria are met
5. Uncontrolled pleural effusion, pericardial effusion, or ascites. Patients with indwelling catheters (e.g., PleurX®) are allowed
6. Uncontrolled tumor-related pain. Patients requiring narcotic pain medication must be on a stable regimen at study entry. Symptomatic lesions (e.g., bone metastases or metastases causing nerve impingement) amenable to palliative radiotherapy should be treated prior to

randomization. Asymptomatic metastatic lesions whose further growth would likely cause functional deficits or intractable pain (e.g., epidural metastasis that is not presently associated with spinal cord compression) should be considered for loco-regional therapy if appropriate prior to randomization

7. Ionized calcium > 1.2 x UNL. The use of bisphosphonates is allowed
8. Pregnant or breastfeeding
9. Evidence of significant uncontrolled concomitant disease that could affect compliance with the protocol or interpretation of results, including significant liver disease (such as cirrhosis, uncontrolled major seizure disorder, or superior vena cava syndrome)
10. Significant cardiovascular disease, such as New York Heart Association (NYHA) cardiac disease (Class II or greater), myocardial infarction within 3 months prior to randomization, unstable arrhythmias, or unstable angina. Patients with a known left ventricular ejection fraction (LVEF) < 40% will be excluded. Patients with known coronary artery disease, congestive heart failure not meeting the above criteria, or LVEF < 50% must be on a stable medical regimen that is optimized in the opinion of the treating physician, in consultation with a cardiologist if appropriate
11. Severe infection within 14 days prior to randomization, requiring hospitalization
12. Received oral or IV antibiotics within 1 week prior to Cycle 1, Day 1. Patients receiving routine antibiotic prophylaxis (e.g., to prevent chronic obstructive pulmonary disease exacerbation or for dental extraction) are eligible
13. Major surgical procedure within 14 days prior to randomization or anticipation of the need for a major surgical procedure during the course of the study other than for diagnosis. Placement of central venous access catheter(s) is not considered a major surgical procedure and is therefore permitted
14. A history of severe allergic, anaphylactic, or other hypersensitivity reactions to chimeric or humanized antibodies or fusion proteins
15. Known hypersensitivity or allergy to biopharmaceuticals produced in Chinese hamster ovary cells or any component of the atezolizumab formulation
16. Known hypersensitivity to doxorubicin or cyclophosphamide or any of their excipients
17. A history of autoimmune disease that has required systemic treatment in the past 2 years (i.e. with use of disease modifying agents, corticosteroids or immunosuppressive drugs). Replacement therapy (e.g., thyroxin, insulin, or physiologic corticosteroid replacement therapy for adrenal or pituitary insufficiency, etc.) is not considered a form of systemic treatment. Patients with eczema, psoriasis, lichen simplex chronicus or vitiligo with dermatologic manifestations only (e.g., no psoriatic arthritis) are permitted provided that they meet all of the following conditions:
    a. Rash must cover less than 10% of body surface area.
    b. Disease is well controlled at baseline and only requiring low potency topical steroids
    c. No acute exacerbations of underlying condition within the last 12 months (not requiring PUVA [psoralen plus ultraviolet A radiation], methotrexate, retinoids, biologic agents, oral calcineurin inhibitors, high potency or oral steroids)
18. Undergone allogeneic stem cell or solid organ transplantation
19. A history of idiopathic pulmonary fibrosis (including pneumonitis) drug-induced pneumonitis, organizing pneumonia (i.e., bronchiolitis obliterans, cryptogenic organizing pneumonia), or evidence of active pneumonitis on screening chest CT scan. History of radiation pneumonitis in the radiation field (fibrosis) is permitted

20. A positive test for HIV
21. Active hepatitis B (defined as having a positive hepatitis B surface antigen [HBsAg] test at screening) or hepatitis C. Patients with past hepatitis B virus (HBV) infection or resolved HBV infection (defined as having a negative HBsAg test and a positive antibody to hepatitis B core antigen [anti-HBc] antibody test) are eligible. Patients positive for hepatitis C virus (HCV) antibody are eligible only if polymerase chain reaction (PCR) is negative for HCV RNA
22. Active tuberculosis
23. Currently receiving study therapy or has participated in a study of an investigational agent and received study therapy or used an investigational device within 4 weeks of the first dose of treatment
24. Received treatment with immune checkpoint modulators, including anti−CTLA-4, anti−PD-1, or anti−PD-L1 therapeutic antibodies
25. Received treatment with systemic immunostimulatory agents (including but not limited to interferons or IL-2) within 4 weeks or five half-lives of the drug (whichever is shorter) prior to randomization
26. Received treatment with systemic corticosteroids or other systemic immunosuppressive medications (including but not limited to prednisone, dexamethasone, cyclophosphamide, azathioprine, methotrexate, thalidomide, and anti−tumor necrosis factor [TNF] agents) within 2 weeks prior to randomization, or anticipated requirement for systemic immunosuppressive medications during the trial
    a. Patients who have received acute, low-dose, systemic immunosuppressant medications (e.g., a one-time dose of dexamethasone for nausea) may be enrolled in the study
    b. Patients with a history of allergic reaction to IV contrast requiring steroid pre-treatment should have baseline and subsequent tumor assessments performed using MRI
    c. The use of inhaled corticosteroids for chronic obstructive pulmonary disease, mineralocorticoids (e.g., fludrocortisone) for patients with orthostatic hypotension, and low-dose supplemental corticosteroids for adrenocortical insufficiency are allowed
27. Received anti-cancer therapy (medical agents or radiation) within 1 week prior to study Cycle 1, Day 1.
28. A history or current evidence of any condition, therapy, or laboratory abnormality that might confound the results of the trial, interfere with the subject’s participation for the full duration of the trial, or is not in the best interest of the subject to participate, in the opinion of the treating Investigator
29. Known psychiatric or substance abuse disorders that would interfere with cooperation and the requirements of the trial
30. Any reason why, in the opinion of the investigator, the patient should not participate. This includes a careful evaluation of whether standard therapy is preferable to the study therapy, for the individual patient.

# 6.0 STUDY TREATMENT

## 6.1 Overview of Study Treatment

**Study arm A:** Placebo combined with pegylated liposomal doxorubicin and cyclophosphamide

**Study arm B:** Atezolizumab combined with pegylated liposomal doxorubicin and cyclophosphamide

**Dosage:**

- Atezolizumab (or placebo) will be administered intravenously 840mg every 2nd week until disease progression or for a maximum of 24 months. The study leadership may for individual patients and with the consent of Roche extend or re-start administration of atezolizumab, based on an evaluation of the patient´s best interest.
- Chemotherapy will be administered as follows:
    - Pegylated liposomal doxorubicin 20 mg/$m^2$ i.v. every 2nd week. An upper limit of 44 mg per dose will be applied to patients with a body surface area >2.2 $m^2$
    - Cyclophosphamide tablets 50 mg per day, daily as continuous treatment for the first 2 weeks in each 4 week period (i.e. every second 2-week cycle)
    - No upper limit for the number of cycles of pegylated liposomal doxorubicin/cyclophosphamide. Heart function will be monitored with regard to pegylated liposomal doxorubicin

The patients will continue study treatment until disease progression per iRECIST (iCPD), death, withdrawal of consent, investigator’s decision or up to 24 months. If the investigator considers that a patient with iUPD should not continue study treatment, due to clinical deterioration or for other reasons, the treatment should be discontinued. If chemotherapy is paused for other reasons than iCPD, and the patient does not start any non-study therapy, atezolizumab may be continued until 24 months after Cycle 1, Day 1. This period may allow for tumor-specific T cells to expand and provide information on the ability of atezolizumab inducing durable responses.
Patients that discontinue treatment due to disease progression will have safety follow-up visits after 1 and 3 months. Patients who do not develop diseased progression will have follow-up visits every 3rd month for 12 months after end of therapy. Survival data will be collected from the national registry and local hospitals/GPs until end of study. This includes information on the cause of death.

## 6.2 Rationale for chemotherapy regimen

The chemotherapy regimen is regarded as appropriate therapy for this patient group, without atezolizumab. The regimen is expected to be well tolerated and applicable to most metastatic TNBC patients with ECOG 0-1, while also being sufficiently potent to suit those with an excellent performance status.

The chemotherapy regimen and dosing schedule has been tailored to aid the effect of atezolizumab, which depends on immune effector cells for its activity. First, the chosen chemotherapeutic agents (anthracyclines and cyclophosphamide) are known to induce immunogenic cell death. Second, we

apply the outlined dosage regimen, rather than a high dose regimen administered every third/fourth week, in order to maintain the leukocyte counts and the ability of the effector immune cells to respond. Anthracyclines are routinely administered at intervals ranging from one to four weeks in metastatic TNBC patients. Tregs and MDSCs represent important mediators of tumor tolerance and may oppose the effect of atezolizumab. The metronomic cyclophosphamide dosage chosen in the present study has been widely used to counter Tregs and MDSCs and is also considered safe, as it has been combined with multiple other chemotherapeutic agents without causing important toxicity (10). We include 14-day intervals without cyclophosphamide to allow for unsuppressed T cell proliferation and activity, which may be important for the atezolizumab effect.

We will use pegylated liposomal doxorubicin to minimize the adverse effects of anthracyclines on the heart and allow for continued treatment beyond the otherwise mandatory anthracycline limits. The possibility of long term treatment is important in order to appropriately test checkpoint inhibitors like atezolizumab, as these drugs are known to induce durable responses in other patient groups. Pegylated liposomal doxorubicin is also administered without any need for corticosteroids, which is desirable for immunotherapy.

The standard pegylated liposomal doxorubicin dose for breast cancer is 40-50 mg/m2 every 4$^{th}$ week. In Norway, the most widely used dose is 40 mg/m2. The dose chosen in our study is expected to be well tolerated, as the 40 mg/m2 is divided into two doses of 20 mg/m2 given every 2$^{nd}$ week. Some studies in breast cancer have used pegylated liposomal doxorubicin at 15-30 mg/m2 every 2$^{nd}$ week (10-14), or in combination with cyclophosphamide (500 mg/m2), and 5-fluorouracil (500 mg/m2) every 3$^{rd}$ week (15). A dose of 20 mg/m2 has been well tolerated in combination with cyclophosphamide (50 mg/day) even in fragile, older patients (14) and is also tolerated by HIV positive patients with Kaposi sarcoma. In our study, a detailed dose reduction plan will be outlined in case of adverse effects.

## 6.3 Rationale for Atezolizumab Dosage

The fixed dose of 840 mg (equivalent to an average body weight−based dose of 15 mg/kg q3w) was selected on the basis of both nonclinical studies and clinical data from Study PCD4989g. A more detailed description regarding nonclinical and clinical pharmacology of atezolizumab is provided in the Investigator's Brochure. The target exposure for atezolizumab has been projected on the basis of nonclinical tissue distribution data in tumor-bearing mice, target-receptor occupancy in the tumor, the observed atezolizumab interim pharmacokinetics in humans, and other factors. The target trough concentration ($C_{trough}$) has been projected to be 6 μg/mL. Anti-tumor activity has been observed across doses from 1 mg/kg to 20 mg/kg. In study PCD4989g, the maximum tolerated dose (MTD) of atezolizumab was not reached, and no DLTs have been observed. The available data suggest that the 15 mg/kg atezolizumab q3w regimen (or fixed-dose equivalent) would be sufficient to maintain $C_{trough} \geq 6$ μg/mL. A fixed dose of 1200 mg has been selected for when atezolizumab is administered q3w (equivalent to an average body weight−based dose of 15 mg/kg). For the q2w dosing interval used in this study, the corresponding fixed dose is 800 mg. Because atezolizumab is formulated at a concentration of 60 mg/mL, 800 mg corresponds to a volume of 13.33 mL. In the interest of simplifying administration, the exact dose used in this study will be 840 mg, corresponding to a volume of 14 mL, which can be accurately administered with a single syringe. The 840-mg dose is not expected to result in meaningfully different exposures compared with an 800-mg dose. A dose of 840mg q2w is also used in study NCT02425891.

### 6.4 Risk benefit assessment

Immunotherapy with checkpoint inhibitors have produces durable clinical responses across several tumor forms(16, 17). The PD1/PD-L1 blockers have showed particularly strong efficacy and are generally well tolerated. In breast cancer, phase I/II studies with PD1/PD-L1 blockers as monotherapy have shown clinical responses in subsets of TNBC patients, and limited adverse effects(1). Atezolizumab has been approved by the FDA and EMA for BC, based on the IMpassion130 trial combining atezolizumab with nab-paclitaxel against metastatic TNBC(9). IMpassion130 only showed efficacy in patients with evidence of immune activation up front, in the form of PD-L1 expression (Ventana SP142 IC≥1%).

Further, as outlined above (1.1.), there is strong evidence indicating that the immune response is important for the clinical outcome in TNBC and predictive for the effect of anthracyclines and cyclophosphamide(4-6). Taken together, there is a strong rationale for combining PD-L1 blockade with anthracyclines and cyclophosphamide in TNBC patients(7).

In 2019, immunotherapy was for the first time approved by the FDA and EMA for BC, based on the IMpassion130 trial combining atezolizumab with chemotherapy against metastatic TNBC(9). This was the first randomized study demonstrating efficacy of any non-chemotherapy drug against TNBC. An effect was only found in patients with evidence of immune activation up front, in the form of PD-L1 expression (Ventana SP142 IC≥1%). In the ALICE trial, we aim at triggering sensitivity to checkpoint blockade in patients that are otherwise not responsive, by use of “immunogenic chemotherapy”, as outlined above.

The safety data for atezolizumab is described below (10.1.1) and in the Investigator´s Brochure. Atezolizumab has a mild toxicity profile. The proportion of patients who discontinued atezolizumab due to adverse events has been similar when the drug has been used as a single agent or in combination with chemotherapy. The present trial applies the same atezolizumab dosage and schedule as in the ongoing phase III trial NCT02425891 (WO29522), which is conducted in a similar patient population (first line treatment for metastatic TNBC). In trial NCT02425891, atezolizumab is also combined with chemotherapy, i.e. with nab-paclitaxel.

In summary, the study treatment combining atezolizumab with anthracyclines and cyclophosphamide offers potential clinical benefit for the selected patient group. The risk of important side effects is limited. We thus consider that the potential benefit outweighs the risks associated with the study treatment.

### 6.5 Administration of Atezolizumab/Placebo

Atezolizumab or placebo infusions will be administered per the instructions outlined in Table 2.

The atezolizumab drug product is provided in a single-use, 20cc USP/Ph. Eur. Type 1 glass vial as a colorless-to-slightly-yellow, sterile, preservative-free clear liquid solution intended for IV administration. The vial contains ~20 mL (1200 mg) of atezolizumab solution.

Atezolizumab vials must be refrigerated at 2°C-8°C (36°F-46°F) upon receipt until use. Vials should not be used beyond the expiration date provided by the manufacturer. Atezolizumab must be prepared/diluted under appropriate aseptic conditions for IV infusion as it does not contain antimicrobial preservatives. The solution for infusion should be used immediately to limit microbial growth in case of potential accidental contamination. Any unused portion of drug left in a vial should be discarded. Vial contents should not be frozen or shaken and should be protected from direct sunlight.

Further details on the formulation, storage and handling of atezolizumab are provided in the atezolizumab Investigator's Brochure and the Pharmacy Manual.

The dose of Atezolizumab in this study will be 840 mg administered by intravenous infusion every 2 weeks (14 [± 3] days). Administration of Atezolizumab will be performed in a setting with emergency medical facilities and staff who are trained to monitor for and respond to medical emergencies. Atezolizumab will be delivered in 250 mL 0.9% NaCl IV infusion bags with product contacting surfaces of polyvinyl chloride (PVC) or polyolefin (PO) and IV infusion lines with product contacting surfaces of PVC or polyethylene (PE) and 0.2 μm in-line filters (filter membrane of polyethersulfone [PES]). No incompatibilities have been observed between Atezolizumab and these infusion materials (bags and infusion lines). The initial dose of Atezolizumab will be delivered over 60 (± 15) minutes.

If the first infusion is tolerated without infusion-associated adverse events, the second infusion may be delivered over 30 (± 10) minutes. If the 30-minute infusion is well tolerated, all subsequent infusions may be delivered over 30 (± 10) minutes. For first infusion of study treatment, the patient's vital signs should be determined within 60 minutes before the infusion; during, and after the infusion if clinically indicated. For subsequent infusions, vital signs will be collected within 60 minutes before infusion and at the end of the infusion, if clinically indicated. Patients will be informed about the possibility of delayed post-infusion symptoms and instructed to contact their study physician if they develop such symptoms. No premedication will be allowed for the first dose of Atezolizumab. Premedication may be administered for Cycles ≥ 2 at the discretion of the treating physician.

Placebo treatment will consist of an identical-looking intravenous infusion of NaCl 0.9% administered in the same manner. The preparation of active drug dilution/placebo, according to the respective randomization code, will be facilitated by the hospital pharmacy, in order to maintain the double blind.

Table 2

| First Infusion | Subsequent Infusions |
|---|---|
| • No premedication is administered.<br>• Record patient's vital signs (heart rate, respiratory rate, blood pressure, and temperature) within 60 minutes before starting infusion.<br>• Infuse 14 mL atezolizumab (840 mg) in 250 mL NaCl) over 60 (± 15) minutes.<br>• Record patient's vital signs (heart rate, respiratory rate, blood pressure, and temperature) during and after the infusion if clinically indicated<br>• Patients will be informed about the possibility of delayed symptoms following infusion and instructed to contact their study physician if they develop such symptoms. | • If patient experienced infusion-related reaction during any previous infusion, premedication with antihistamines may be administered for Cycles ≥ 2 at the discretion of the treating physician.<br>• Record patient's vital signs (heart rate, respiratory rate, blood pressure, and temperature) within 60 minutes before starting infusion.<br>• If the patient tolerated the first infusion well without infusion-associated adverse events, the second infusion may be *administered* over 30 (± 10) minutes.<br>• If no reaction occurs, subsequent infusions may be *administered* over 30 (± 10) minutes<br>Continue to record vital signs within 60 minutes before starting infusion and during and after the infusion if clinically indicated.<br>• If the patient had an infusion-related reaction during the previous infusion, the subsequent infusion must be *administered* over 60 (± 15) minutes.<br>Record patient's vital signs (heart rate, respiratory rate, blood pressure, and temperature) during and after the infusion if clinically indicated. |

## 6.6 Administration of pegylated liposomal doxorubicin

Pegylated liposomal doxorubicin will be labelled by the study hospital pharmacies as IMP. The drug is to be administrated after the administration of atezolizumab/placebo and according to the standard procedure at the study hospitals and the European guidelines (ema.europa.eu). The use of prophylactic antiemetics including netupitant/aprepitant, ondansetron/palonosetron and metoclopramide is allowed and encouraged. Pyridoxine is allowed. Corticosteroids should be avoided if possible.

For information on the formulation, packaging, and handling of pegylated liposomal doxorubicin see the local prescribing information.

### 6.7 Administration and compliance registration for cyclophosphamide

Cyclophosphamide will be labelled as IMP by the study hospital pharmacies or trained personnel at the site according to local written procedure, and administrated by the patient. The compliance will be monitored by the study personnel every 8$^{th}$ week, by counting of the number of tablets in each labelled box and registering of this number in the eCRF.

For information on the formulation, packaging, and handling of Cyclophosphamide, see the local prescribing information.

## 7.0 OUTCOME MEASURES

### 7.1 Safety Outcome Measures

The safety outcome measures will be evaluated in the ITT population, as follows:

- Incidence, nature, and severity of adverse events graded according to NCI CTCAE v4.0 and AESIs for atezolizumab.
- Changes in vital signs, physical findings, and clinical laboratory results

### 7.2 Efficacy Outcome Measures

The primary efficacy outcome measure is to be assessed in all patients evaluable per protocol (PP), and in the PD-L1-positive PP subpopulation, as follows:

- PFS, defined as the time from randomization to the time of disease progression (as assessed by iRECIST) or death from any cause during the study

The secondary efficacy outcome measures will be assessed in the **PP population, ITT population, PD-L1-negative PP population and PD-L1-positive PP population** as follows:

- Overall survival (OS), defined as the time from the date of randomization to the date of death from any cause
- Objective tumor response rate (ORR), defined as the proportion of patients with an objective tumor response (either partial response [PR] or complete response [CR] per investigator using iRECIST)
- Durable response rate (DRR), defined as the proportion of patients with an objective tumor response lasting at least 24 weeks
- Clinical benefit rate (CBR), defined as the proportion of patients with an objective tumor response or with stable disease lasting at least 6 months, as assessed at the 6 month evaluation (week 24)
- Duration of objective response (DOR) among patients with an objective response
- PFS in the ITT population and PD-L1-negative subpopulation assessed by iRECIST
- PFS, ORR, DRR, CBR and DOR assessed by RECIST v1.1

Other secondary/exploratory outcome measures:

- Assessment of immunological response
- Identification of biomarkers for clinical response, toxicity and immune response, including assessment of PD-L1 expression, mutation load and immune gene expression

- Characterization of tumor evolution and changes in immunological milieu induced by the combination therapy (atezolizumab+chemo), as compared to chemo only
- Development in FQ score(18). The analyses will include time to deterioration (TTD) in the FQ score, defined by a minimally clinically important difference (MCID) of ≥ 3 points. The maximum total FQ score is 33 points. For mean score, a separate analysis will be performed for subjects with a baseline FQ score ≥ 21 points
- Development in NRS pain intensity score for average pain the last 24 hours (corresponding to one item in Brief Pain Inventory). The analyses will include TTD in the pain intensity score, defined by a minimally clinically important difference (MCID) of ≥ 2 points (scale 0-10). For mean score, a separate analysis will be performed for subjects with a baseline score ≥ 4 points
- Mean changes and TTD in the Global health status / quality of life score of the EORTC QLQ-C15-PAL, defined by a MCID ≥ 20 points at patient individual level. A change of ≥10 points is considered to be of clinical importance at group level. The development of other scales and items of QLQ-C15 will also be recorded

# 8.0 TRIAL FLOW CHART

## 8.1 Study Flow Chart

| **Trial Period:** | **Screening Phase** | | **Treatment Phase** | | | | | **Post-Treatment** | | |
|---|---|---|---|---|---|---|---|---|---|---|
| Treatment Cycle (C)/Title:<br>(Each cycle is 2 weeks) | Informed consent (Visit 1) | Main Study Screening (Visit 2) | To be repeated | | | | Treatm ent disconti nuation | Safety Follow -up | Patients not progressed during treatment<br>Follow Up Visits | Patients progressed during treatment<br>Follow Up Visit |
| | | | C1 | C2 | C3 | C4 | | | | |
| Scheduling Window (Days)[a]: | | -21 to -1 | ± 3 | ± 3 | ± 3 | ± 3 | At time of discon | 30 days post discon ± 7 days | Every 12 weeks ± 7 days post discon for 12 months, or until disease progression | 12 weeks ± 7 days post discon |
| **Administrative Procedures** | | | | | | | | | | |
| Informed Consent[b] | x | | | | | | | | | |
| Inclusion/Exclusion Criteria | | x | | | | | | | | |
| Demographics and Medical History | | x | | | | | | | | |
| Prior and Concomitant Medication Review[c] | | x | x | x | x | x | x | x | | |
| Trial Treatment Administration | | | x | x | x | x | | | | |
| Post-study anticancer therapy status | | | | | | | | | x | x |
| **Clinical Procedures/Assessments** | | | | | | | | | | |

| Trial Period: | Screening Phase | | Treatment Phase | | | | | Post-Treatment | | |
|---|---|---|---|---|---|---|---|---|---|---|
| Treatment Cycle (C)/Title:<br>(Each cycle is 2 weeks) | Informed consent (Visit 1) | Main Study Screening (Visit 2) | To be repeated | | | | Treatment discontinuation | Safety Follow-up | Patients not progressed during treatment Follow Up Visits | Patients progressed during treatment Follow Up Visit |
| | | | C1 | C2 | C3 | C4 | | | | |
| Scheduling Window (Days)[a]: | | -21 to -1 | ± 3 | ± 3 | ± 3 | ± 3 | At time of discon | 30 days post discon ± 7 days | Every 12 weeks ± 7 days post discon for 12 months, or until disease progression | 12 weeks ± 7 days post discon |
| Review Adverse Events[d,e] | | x | x | x | x | x | x | x[e] | x[e] | x[e] |
| Full Physical Examination | | x | x[f] | | | | | x | | |
| Directed Physical Examination | | | | x | x | x | x | | x | x |
| Vital Signs and Weight | | x | x | x | x | x | x | x | x | x |
| ECOG Performance Status | | x | x | x | x | x | x | x | x | x |
| Electrocardiogram (ECG) | | x | x[f] | | | | | | | |
| LVEF assessment | | x[f] | | | | | | | | |
| **Laboratory Procedures/Assessments: analysis performed by LOCAL laboratory** | | | | | | | | | | |
| Pregnancy Test – Urine or Serum -HCG[g] | | x | | | | | x | x | | |
| INR and aPTT[h] | | x | | | | | | | | |
| CBC with Differential[i] | | x | x | x | x | x | x | x | x | x |
| Comprehensive Serum Chemistry Panel[j] | | x | x | x | x | x | x | x | x | x |
| Urine analysis[j] | | x | x[f] | | | | x | x | | |
| FT4 and TSH, anti TPO[j] | | x | x[f] | | | | x | x | x | x |
| MUC-1, CA 125, CEA, amylase | | x | x[f] | | x | | x | | x | x |
| HIV/HCV/HBV-tests, lipase | | x | | | | | | | | |
| **Efficacy Measurements** | | | | | | | | | | |
| Tumor Imaging[i, l] | | x | x[f] | | | | x | | x | |
| **Tumor Biopsies/Archival Tissue Collection/Correlative Studies Blood** | | | | | | | | | | |
| Tumor biopsy[m] | | x | | | x | | x | | | |
| PBMC collection[n] | | (x)[n] | x[n] | x | | | x | | x[k] | x |
| Plasma/serum sample[o] | | | x | x | x | | x | | x[k] | x |
| Urine sample | | x | x[f] | | | | x | | | |
| Faeces sample | | x | x[f] | | | | | | | |
| CTC collection (only if sufficient resources) | | | x | | x | | x | | | |
| **Patient Reported Outcomes** | | | | | | | | | | |
| FQ, NRS, EORTC QLQ-C15-PAL [p] | | | x | | | | x | | x | x |

a. General, assessments/procedures are to be performed on Day 1 and prior to the first dose of treatment for each cycle unless otherwise specified. Treatment cycles are 2 weeks; however the treatment cycle interval may be increased due to toxicity according to the dose modification guidelines provided in Section 10.1.2

b. Written consent must be obtained prior to performing any protocol specified procedure. Results of a test performed prior to the subject signing consent as part of routine clinical management are acceptable in lieu of a screening test if performed within the specified time frame in the protocol. Subject number will be assigned when the study informed consent is signed.

c. Prior medications – Record all medications taken within 30 days of screening visit. Concomitant medications – Enter new medications started during the trial through the Safety Follow-up visit. Record all medications taken for AEs.

d. AEs and laboratory safety measurements will be graded per NCI CTCAE version 4.0. All AEs, whether gradable by CTCAE or not, will also be evaluated for seriousness.

e. After informed consent has been obtained but prior to initiation of study drug, only serious adverse events caused by a protocol-mandated intervention should be reported. After initiation of study drug, all adverse events will be reported until 30 days after the last dose of study treatment or until initiation of another anti-cancer therapy, whichever occurs first. After this period, investigators should report any serious adverse events and adverse events of special interest that are believed to be related to prior treatment with study drug. The investigator should follow each adverse event until the event has resolved to baseline grade or better, the event is assessed as stable by the investigator, the patient is lost to follow-up, or the patient withdraws consent. Every effort should be made to follow all serious adverse events considered to be related to study drug or trial-related procedures until a final outcome can be reported.

f. The full clinical examination is to be replaced by a directed clinical examination at C1 day 1. ECG is to be performed at screening (not C1), C5 day 1 and every 4$^{th}$ cycle thereafter. Left ventricular ejection fraction (LVEF) is to be measured at screening, cycle 9 and every 8$^{th}$ week thereafter, if administration of pegylated liposomal doxorubicin is continued. LVEF may be measured by Multi Gated Acquisition Scan (MUGA) or echocardiography. FT4, TSH, anti-TPO and urine analyses are not to be performed at C1 day 1, but at screening and at day 1 of C5 and every 4$^{th}$ cycle thereafter. MUC-1, CA 125, CEA and amylase analyses are not to be performed at C1 day 1, but at screening and at day 1 of C3 and every 2nd cycle thereafter. Tumor assessment is not to be performed at C1 day 1, but at screening, and as indicated in footnote I and l a. Urine sampling for the biobank is only to be performed at screening, day 1 of C5 and at time of progression. Faeces sampling is only to be performed at screening and at day 1 of C5.

g. For women of reproductive potential, a serum pregnancy test should be performed within 7 days prior to first dose of trial treatment. Pregnancy tests (serum and/or urine tests) should be repeated if required by local guidelines.

h. Coagulation factors (PT/INR and aPTT) should be tested as part of the screening procedures for all subjects. Any subject receiving anticoagulant therapy should have coagulation factors monitored closely throughout the trial.

i. Tumor assessments performed as standard of care prior to obtaining informed consent and within 21 days of Cycle 1, Day 1 may be used rather than repeating tests. All measurable and evaluable lesions should be assessed and documented at the screening visit. Radiologic imaging performed during the screening period should consist of 1) CT of the chest/abdomen/pelvis, alternatively MRI 2) bone scan (MRI, PET scan or scintigraphy), and 3) any other imaging studies (CT neck, plain films, etc.) as clinically indicated by the treating physician. No anti-tumor treatment is allowed between the time point for baseline radiological scans and start of study therapy. The same radiographic procedures and technique must be used throughout the study for each patient (e.g., if the patient had CT chest/abdomen/pelvis performed during screening, then she/he should subsequently undergo CT performed using the same radiologic protocol throughout the remainder of the study). Results must be reviewed by the investigator before dosing at the next cycle. Tumor assessments will be performed at baseline, every 8 weeks from C1 day1 (± 1 week) for the first 12 months following randomization, and every 12 weeks (± 10 days ) thereafter, with additional scans as clinically indicated. If iUPD is detected, a new radiological scan should be performed after 4-8 weeks, in accordance with iRECIST. Tumor response will be evaluated using both iRECIST and RECIST v1.1. In the absence of disease progression per iRECIST, tumor assessments should continue regardless of whether patients discontinue study treatment, unless they withdraw consent or the study is terminated by the Sponsor, whichever occurs first.

j. Unresolved abnormal labs that are drug related AEs should be followed until resolution. Labs do not need to be repeated after the end of treatment if labs are within normal range.

k. For patients not progressed during treatment, plasma/serum and PBMC collection to be performed at first FU visit only (12 weeks after discontinuation). The Sponsor may ask for additional blood /PBMCs in selected cases.

l. In subjects who discontinue study therapy without confirmed disease progression, a radiologic evaluation should be performed at the time of treatment discontinuation (i.e., date of discontinuation ± 4 weeks). If a previous scan was obtained within 4 weeks prior to the date of discontinuation, then a scan at treatment discontinuation is not mandatory. Radiological assessments performed as standard of care can replace the tumor scans at follow-up visits, if performed ± 4 weeks of the scheduled time point.

m. Fresh frozen and FFPE tumor biopsies before start of treatment (mandatory), 4 weeks from C1 day 1 (± 5 days), 6 months from C1 day 1 (± 10 days) and at time of treatment discontinuation (fine needle aspiration is not sufficient). The pre-study biopsy may be obtained any time after signed informed consent. Archival tumor tissue can be used instead of pretreatment biopsy, but must be obtained within three months of Cycle 1, Day 1. No anti-tumor treatment is allowed between the time point for biopsy and study entry. If the archival biopsy does not include fresh frozen tumor, a new pre-treatment biopsy for preservation as fresh-frozen material is mandatory.

n. PBMC; at day 1 of C1 (or screening) and day 1 of C2, C5, C9, C13, C25,time of progression and the follow-up visit after 12 weeks. 100 ml ACD blood to be drawn at C1day1/screening. 50 ml ACD blood to be drawn at all later time points. Samples for PBMC collection should always be taken before infusion.

o. Plasma/serum to be collected at day 1 and day 2 of C1 and C5, and at the day of atezolizumab injection (day 1) of C2, C3, C4, C6, C9, C13, C25,time of progression and the follow-up visit after 12-weeks.

p. PRO forms to be completed at day 1 (+/- 7 days) of C1, C5, C9, C13, C25, C39 and at time of progression. The forms should be completed prior to the evaluation visit with the study doctor.

# 9.0 TRIAL PROCEDURES

## 9.1 Trial Procedures

The Trial Flow Chart, Section 8.1, summarizes the trial procedures to be performed at each visit. Individual trial procedures are described in detail below. It may be necessary to perform these procedures at unscheduled time points if deemed clinically necessary by the Investigator. No anti-cancer treatment is allowed between the time point for screening assessments, including baseline radiological scans, and start of study therapy.

Furthermore, additional evaluations/testing may be deemed necessary by the Sponsor for reasons related to subject safety. In some cases, such evaluation/testing may be potentially sensitive in nature (e.g., HIV, Hepatitis C, etc.), and thus local regulations may require that additional informed consent be obtained from the subject. In these cases, such evaluations/testing will be performed in accordance with those regulations.

### 9.1.1 Administrative Procedures

#### 9.1.1.1 Informed Consent

The Investigator must obtain documented consent from each potential subject prior to participating in a clinical trial and prior to any trial-related procedures.

##### 9.1.1.1.1 General Informed Consent

Consent must be documented by the subject’s dated signature or by the subject’s legally acceptable representative’s dated signature on a consent form along with the dated signature of the person conducting the consent discussion.

A copy of the signed and dated consent form should be given to the subject before participation in the trial.

The initial informed consent form, any subsequent revised written informed consent form and any written information provided to the subject must receive the IRB/ERC’s approval/favorable opinion in advance of use. The subject or his/her legally acceptable representative should be informed in a timely manner if new information becomes available that may be relevant to the subject’s willingness to continue participation in the trial. The communication of this information will be provided and documented via a revised consent form or addendum to the original consent form that captures the subject’s dated signature or by the subject’s legally acceptable representative’s dated signature.

Specifics about a trial and the trial population will be added to the consent form template at the protocol level.

The informed consent will adhere to IRB/ERC requirements, applicable laws and regulations and Sponsor requirements.

#### 9.1.1.2 Inclusion/Exclusion Criteria

All inclusion and exclusion criteria will be reviewed by the Investigator or qualified designee to ensure that the subject qualifies for the trial.

#### 9.1.1.3 Medical History

A medical history will be obtained by the Investigator or qualified designee.  Medical history will include all active conditions, and any condition diagnosed within the prior 10 years that are considered to be clinically significant by the Investigator.  Details regarding the disease for which the subject has enrolled in this study will be recorded separately and not listed as medical history.

#### 9.1.1.4 Prior and Concomitant Medications Review

##### 9.1.1.4.1 Prior Medications

The Investigator or qualified designee will review prior medication use, including any protocol-specified washout requirement, and record prior medication taken by the subject the last 30 days before screening visit.  Treatment for the disease for which the subject has enrolled in this study will be recorded separately and not listed as a prior medication.

##### 9.1.1.4.2 Concomitant Medications

The Investigator or qualified designee will record medication, if any, taken by the subject during the trial.

##### 9.1.1.4.3 Disease Details

The Investigator or qualified designee will obtain prior and current details regarding disease status.

##### 9.1.1.4.4 Prior Treatment Details

The Investigator or qualified designee will review all prior cancer treatments including systemic treatments, radiation and surgeries.

##### 9.1.1.4.5 Subsequent Anti-Cancer Therapy Status

The Investigator or qualified designee will review all new anti-neoplastic therapy initiated after the last dose of trial treatment.  If a subject initiates a new anti-cancer therapy within 30 days after the last dose of trial treatment, the 30 day Safety Follow-up visit must occur before the first dose of the new therapy.

#### 9.1.1.5 Assignment of Subject Number

All consented subjects will be given a unique subject number that will be used to identify the subject for all procedures that occur during the screening period, and for all subjects eligible for treatment, during the treatment and follow-up period.

Each subject will be assigned only one subject number. Subject numbers must not be re-used for different subjects. Any subject who is screened multiple times will retain the original subject number assigned at the initial screening visit.

#### 9.1.1.6 Randomization and emergency unblinding

Randomization will be performed using the eCRF. The subject will be allocated to a randomization number and the randomization number will be sent to the hospital pharmacy. The pharmacy keeps the randomization listing and prepares the infusion according to the given randomization number. The pharmacy will keep a log of all infusions prepared. All other study personnel will be blinded to the treatment code.

Emergency unblinding is possible using the eCRF. An access will be created for emergency unblinding only, and the designated persons will have access to the treatment code for the relevant subject only. The eCRF audit trail will track all access to the subjects. The designated person will have 24h access to the eCRF. One of the designated persons will be available to the treating physician on a 24h/7-days a week basis.

#### 9.1.1.7 Electronic Case Report Forms (eCRFs)

The designated investigator staff will enter the data required by the protocol into the eCase report forms (eCRF). The Investigator is responsible for assuring that data entered into the eCRF is complete, accurate, and that entry is performed in a timely manner.

The signature of the investigator will attest the accuracy of the data on each eCRF. If any assessments are omitted, the reason for such omissions will be noted on the eCRFs. Corrections, with the reason for the corrections will also be recorded.

### 9.1.2 Clinical Procedures/Assessments

#### 9.1.2.1 Full Physical Exam

A complete physical examination should include an evaluation of the throat, heart, lungs, abdomen, skin, lymph node regions, musculoskeletal and neurological systems. Any abnormality identified at baseline should be recorded on the General Medical History. Height and weight should be measured and recorded in the CRF.

At subsequent visits (or as clinically indicated), limited, symptom-directed physical examinations should be performed. Changes from baseline abnormalities should be recorded in patient notes. New or worsened clinically significant abnormalities should be recorded as adverse events in the CRF.

The Investigator or qualified designee will perform a complete physical exam during the screening period. Clinically significant abnormal findings should be recorded as medical history. A full physical exam should be performed during screening, and every 8th week during study treatment as well as at the 30 days safety follow-up visit.

#### 9.1.2.2 Directed Physical Exam

For cycles that do not require a full physical exam per the Trial Flow Chart, the Investigator or qualified designee will perform a directed physical exam as clinically indicated prior to trial treatment administration.

#### 9.1.2.3 Vital Signs

The Investigator or qualified designee will take vital signs at screening, prior to the administration of each dose of trial treatment and at treatment discontinuation as specified in the Trial Flow Chart. Vital signs should include temperature, pulse oxygen saturation, weight and blood pressure. Height and respiratory rate will be measured at screening only.

#### 9.1.2.4 Eastern Cooperative Oncology Group (ECOG) Performance Scale

The Investigator or qualified designee will assess ECOG status at screening, prior to the administration of each dose of trial treatment, at discontinuation of trial treatment and during follow-up as specified in the Trial Flow Chart.

#### 9.1.2.5 Cardiac assessment

The left ventricular ejection fraction (LVEF) should be measured at screening, cyclus 9 and every 8th week thereafter, if therapy with pegylated liposomal doxorubicin is continued. LVEF may be measured by MUGA of echocardiography. ECG is to be done at screening and repeated every 4th cyclus. In addition, LVEF-determination, ECG and other cardiac assessments should be performed when clinically indicated. More frequent LVEF monitoring may be warranted if the cumulative dose of doxorubicin exceeds 450 mg/m2.

#### 9.1.2.6 Laboratory tests

Laboratory tests for hematology, chemistry, urine analysis and other relevant tests are specified in Table 3.

Results must be reviewed by the Investigator or qualified designee and found to be acceptable prior to each dose of trial treatment.

Table 3

| **Hematology** | **Chemistry** | **Urinalysis** | **Other** |
|---|---|---|---|
| Hematocrit | Albumin | Blood | Serum β-human chorionic gonadotropin† |
| Hemoglobin | Alkaline phosphatase | Glucose | |

| Hematology | Chemistry | Urinalysis | Other |
|---|---|---|---|
| Platelet count | Alanine aminotransferase (ALT) | Protein | INR |
| WBC (total and differential) | Aspartate aminotransferase (AST) | | aPTT |
| Red Blood Cell Count | Lactate dehydrogenase (LDH) | Microscopic exam if abnormal results are noted | |
| Absolute Neutrophil Count | Total protein | | Free thyroxine (T4) |
| Absolute Lymphocyte Count | CRP | Urine pregnancy test † | Thyroid stimulating hormone (TSH) |
| | Creatinine | | TPO |
| | Uric Acid | | |
| | Calcium | | |
| | Chloride | | Amylase |
| | Glucose | | Lipase |
| | Phosphorus | | HBV sAg and HCV Ab or HCV RNA |
| | Potassium | | HIV-test |
| | Sodium | | |
| | Magnesium | | |
| | Total Bilirubin | | MUC-1<br>CA125<br>CEA |
| | Direct Bilirubin *(If total bilirubin is elevated)* | | Blood for correlative studies |
| † Perform on women of childbearing potential only. | | | |

#### 9.1.2.7 Tumor Imaging and Assessment of Disease

The tumor response will be assessed according to iRECIST (19) as primary method, and by RECIST v1.1 as secondary method. After baseline tumor assessments, evaluation of tumor response will be performed every 8 weeks for the first 12 months following randomization (± 7 days) and every 12 weeks thereafter (± 10 days), with additional scans performed as clinically indicated. The same radiographic procedures used to assess measurable disease sites at screening should be used throughout the study (e.g., the same contrast protocol for CT scans and/or MRI). All known sites of disease must be documented at screening and re-assessed at each subsequent tumor evaluation. A classification of best overall response as stable disease, according to iRECIST or RECIST, will require stable disease at a scan taken >4 weeks after start of study treatment. If no such scan is obtained, and there is no documented disease progression or response, the patient will be considered not evaluable for tumor response. PFS will be recorded until the day on which a post therapy scan was obtained, even if no scan was taken >4 weeks

after start of study treatment. If no tumor assessment was performed after randomization, PFS data will be censored at the date of randomization +1 day.

If the radiological scan shows unconfirmed disease progression (iUPD) according to iRECIST, and the patient is clinically stable, a new scan should be performed between 4 and 8 weeks after the previous scan. If progression is confirmed (iCPD), the study treatment should be stopped. If disease progression is not confirmed, the study treatment should be continued and radiological evaluation should continue as originally planned, unless more frequent scanning is indicated.

If a new confirmation scan cannot be obtained for a patient with iUPD, the date for PD according to RECIST v1.1 should be used in the recording of PD according to iRECIST in this study.

#### 9.1.2.8 Tumor Tissue Collection and Correlative Studies Blood/Urine/Faeces Sampling

Tumor biopsies are to be collected pre (mandatory), during and post therapy (time of progression). Please see Section 11.1 and the study flow chart for information on collection of biopsies, peripheral blood, urine and faeces.

The samples will be processed and stored according to protocols provided by OUS.

#### 9.1.2.9 PD-L1 analysis

The pre-study biopsies will be analyzed by IHC for PD-L1 expression using the Ventana SP142 assay

### 9.1.3 Withdrawal/Discontinuation

When a subject discontinues/withdraws prior to trial completion, all applicable activities scheduled for the final trial visit should be performed at the time of discontinuation. Any adverse events which are present at the time of discontinuation/withdrawal should be followed in accordance with the safety requirements outlined in Section 10.4.

### 9.1.4 Post-Treatment Visits

#### 9.1.4.1 Safety Follow-Up Visit

The mandatory Safety Follow-Up Visit should be conducted approximately 30 days after the last dose of trial treatment or before the initiation of a new anti-cancer treatment, whichever comes first. All AEs that occur prior to the Safety Follow-Up Visit should be recorded. After this period, investigators should report any SAE or AESI up to 90 days after the last dose of trial treatment or until initiation of new systemic anti-cancer therapy, whichever occurs first. Thereafter, investigators should report any SAE or AESI that are believed to be related to prior treatment with study drug. The investigator should follow each adverse event until the event has resolved to baseline grade or better, the event is assessed as stable by the investigator, the patient

is lost to follow-up, or the patient withdraws consent. Every effort should be made to follow all serious adverse events considered to be related to study drug or trial-related procedures until a final outcome can be reported.

#### 9.1.4.2 Follow-up Visits

All subjects who discontinue trial treatment will move into the Follow-Up Phase. The patients that progressed during study treatment will be assessed at one Follow-up visit 12 weeks after discontinuation. Patients who discontinue without progression will be assessed every 12 weeks (± 7 days) for the next 12 months. Every effort should be made to collect information regarding disease status until the start of new anti-neoplastic therapy, disease progression, death, or end of the study. For details, see Study Flow Chart section 8.1.

#### 9.1.4.3 Survival Follow-up

Survival data will be collected for 5 years after inclusion of the last patient. Information related to survival status will be collected from the Norwegian Population Registry. Cause of death will be collected from the patient´s local hospital or GP.

### 9.1.5 Trial handling during the SARS-CoV-2 situation

The challenges arising in 2020 with the extraordinary situation caused by SARS-CoV-2 pandemic may affect the conduct of clinical trials. Every effort should be made to ensure that subjects included in the ALICE-trial receive treatment and follow-up according to protocol. Patient safety should have the highest priority. Any protocol deviation due to the SARS-CoV-2 situation will be documented.

The following adaptations to the protocol may be considered, if required due to the covid-19 situation:

- Omission of the otherwise mandatory pre-study biopsy. This is dependent on the availability of archival tumor tissue for the study biobank, obtained after the last systemic tumor-targeting therapy.

- Physical examination at protocol visits may be omitted, if not clinically indicated, if this is in the best interest of the patient, to limit the risk of transmission of SARS-CoV-2.

- Follow-up visits may be conducted by phone or video conference, if this is in the best interest of the patient.

# 10.0 SAFETY ASSESSMENT

## 10.1 Safety plan

Administration of study treatment will be performed in a setting with emergency medical facilities and staff who are trained to monitor for and respond to medical emergencies. All adverse events and serious adverse events will be recorded during the trial and for up to 30 days after the last dose of study drug or until the initiation of another anti-cancer therapy, whichever occurs first. After this period, investigators should report any SAE or AESI up to 90 days after the last dose of trial treatment or until initiation of new systemic anti-cancer therapy, whichever occurs first. Thereafter, investigators should report any SAE or AESI that are believed to be related to prior treatment with study drug. The potential safety issues anticipated in this trial, as well as measures intended to avoid or minimize such toxicities, are outlined in the following sections.

This is a double-blind study. Study treatment assignment may be unblinded for a serious, unexpected study drug-related toxicity. Emergency unblinding should be a last resort only performed in cases where knowledge of treatment assignment will affect ongoing treatment of the patient.

### 10.1.1 Risks Associated with Atezolizumab

The PD-L1/PD-1 pathway is involved in peripheral tolerance; therefore, such therapy may increase the risk of immune-mediated adverse events, specifically the induction or enhancement of autoimmune conditions.

Atezolizumab is a part of a large and growing clinical development plan. Safety information detailed below is based on the safety profile of atezolizumab across the entire development program, which consists of clinical trials investigating atezolizumab as a monotherapy as well as with other agents (approved and unapproved). The safety profile remains consistent based on the known mechanism of action of atezolizumab. As with all investigational products, unknown side effects may occur. Patients should be monitored closely throughout their participation in clinical studies with atezolizumab. As of 10 May 2016, an estimated total of 6053 patients with solid tumor and hematologic malignancies have received atezolizumab in clinical trial participation as a single agent or in combination with cytotoxic chemotherapy and/or targeted therapy. Safety findings of single-agent atezolizumab across multiple tumor types in the clinical development program are consistent with the known mechanism of action of atezolizumab and the underlying disease. Overall, treatment with atezolizumab is well tolerated, with a manageable adverse event profile. Currently, no maximum tolerated dose, no dose-limiting toxicities, and no clear dose-related trends in the incidence of adverse events have been determined. Across all studies and tumor types, the most commonly reported adverse events with single-agent atezolizumab include fatigue, nausea, decreased appetite, diarrhea, constipation, and cough.

The adverse events observed with atezolizumab in combination with chemotherapy and/or targeted therapies are consistent with the known risks of each study treatment. Systemic immune

activation, characterized by an excessive immune response, is a potential risk associated with atezolizumab when used in combination with another immunomodulating compound.

The percentage of patients who discontinued atezolizumab due to any adverse event is consistent when used as a single agent or in combination with chemotherapy (e.g., 5.4% in Study PCD4989g and 5.8% in Study GP28328, respectively). The percentage of patients with any Grade 5 adverse event was similar when used as a single agent or in combination with chemotherapy (e.g., 1.6% in Study PCD4989g and 1.0% in Study GP28328).

Immune related adverse events are consistent with the role of the PD-L1/PD-1 pathway in regulating peripheral tolerance. Given the mechanism of action of atezolizumab, events associated with inflammation and/or immune-related adverse events are closely monitored during the atezolizumab clinical program. To date immune related adverse events associated with atezolizumab include hepatitis, pneumonitis, colitis, pancreatitis, diabetes mellitus, hypothyroidism, hyperthyroidism, adrenal insufficiency, Guillain-Barré syndrome, myasthenic syndrome/myasthenia gravis, meningoencephalitis, myocarditis, hypophysitis, nephritis, myositis, and severe cutaneous adverse reactions.

Toxicities associated or possibly associated with atezolizumab treatment should be managed according to standard medical practice. Additional tests, such as autoimmune serology or biopsies, should be used to evaluate for a possible immunogenic etiology. Although most immune-related adverse events observed with immunomodulatory agents have been mild and self-limiting, such events should be recognized early and treated promptly to avoid potential major complications. Discontinuation of atezolizumab may not have an immediate therapeutic effect, and in severe cases, immune related toxicities may require acute management with topical corticosteroids, systemic corticosteroids, or other immunosuppressive agents. The investigator should consider the benefit-risk balance prior to further administration of atezolizumab. In patients who have met the criteria for permanent discontinuation, resumption of atezolizumab may be considered if the patient is deriving clinical benefit and has fully recovered from the immune related event. Patients can be rechallenged with atezolizumab only after careful consideration of benefit-risk balance and medical judgment by the investigator. For detailed information regarding management of adverse events associated with atezolizumab, please refer to the most current version of the Atezolizumab Investigator’s Brochure.

### 10.1.2 **Eligibility Criteria**

Eligibility criteria were selected to guard the safety of patients in this trial. Results from the nonclinical toxicology studies with atezolizumab, as well as the nonclinical/clinical data from other PD-L1/PD-1 inhibitors, were taken into account. Specifically, patients at risk for study-emergent autoimmune conditions or with a prior diagnosis of autoimmune disease, patients with evidence of acute infections, and patients who have received a live-attenuated viral vaccine within 4 weeks of randomization are excluded from the study.

### 10.1.3 **Monitoring**

Safety will be evaluated in this study through the monitoring of all serious and non-serious adverse events defined and graded according to NCI CTCAE v4.0. Patients will be assessed for

safety (including laboratory values). Laboratory values must be reviewed prior to each infusion.

General safety assessments will include serial interval histories, physical examinations, and specific laboratory studies, including serum chemistries and blood counts.

During the study, patients will be closely monitored for the development of any adverse events, including signs or symptoms of autoimmune conditions and infection.

All serious adverse events and protocol-defined events of special interest will be reported in an expedited fashion.

Patients will be followed for safety for 30 days following their last dose of study drug.

Patients who have an ongoing study drug related adverse event upon study completion or at discontinuation from the study will be followed until the event has resolved to baseline grade, the event is assessed by the investigator as stable, the patient is lost to follow-up, the patient withdraws consent, or until it has been determined that study treatment or participation is not the cause of the adverse event.

### 10.1.4 Dose Modification

#### 10.1.4.1 General Notes Regarding Dose Modification

Reasons for dose modifications or delays, the supportive measures taken, and the outcomes will be documented in the patient's chart and recorded on the CRF. The severity of adverse events will be graded according to the NCI CTCAE v4.0 grading system.

- Dose reduction of atezolizumab/placebo is not permitted.
- For any concomitant conditions already apparent at baseline, the dose modifications will apply according to the corresponding shift in toxicity grade, if the investigator feels it is appropriate. For example, if a patient has Grade 1 asthenia at baseline that increases to Grade 2 during treatment, this will be considered a shift of one grade and treated as Grade 1 toxicity for dose-modification purposes.
- When several toxicities with different grades of severity occur at the same time, the dose modifications should be according to the highest grade observed.
- If, in the opinion of the investigator, a toxicity is considered to be due to one/two component(s) of the treatment (i.e. atezolizumab/placebo, cyclophosphamide or Pegylated liposomal doxorubicin) and the dose of that component is delayed or modified in accordance with the guidelines below, the other component(s) may be administered if there is no contraindication.
- When treatment is temporarily interrupted because of toxicity caused by atezolizumab/placebo or chemotherapy, the treatment cycles will be restarted such that the atezolizumab/placebo and chemotherapy infusions remain synchronized.

- If it is anticipated that chemotherapy will be delayed by ≥ 10 days, then atezolizumab/placebo should be given without the chemotherapy if there is no contraindication.

The treating physician may use discretion in modifying or accelerating the dose modification guidelines described below depending on the severity of toxicity and an assessment of the risk versus benefit for the patient, with the goal of maximizing patient compliance and access to supportive care.

### 10.1.5 Atezolizumab/Placebo Dose Modification

There will be no dose reduction for atezolizumab or placebo in this study. Patients may temporarily suspend study treatment if they experience an adverse event that requires a dose to be held. If atezolizumab or placebo is held because of adverse events for > 42 days beyond the last dose, then the patient will be discontinued from atezolizumab or placebo treatment and will be followed for safety. If, in the judgment of the investigator, the patient is likely to derive clinical benefit from resuming atezolizumab after a hold > 42 days, study drug may be restarted with the approval of the Sponsor.

If a patient must be tapered off steroids used to treat adverse events, atezolizumab or placebo may be held for > 42 days until steroids are discontinued or reduced to prednisone dose (or dose equivalent) ≤ 10 mg/day. The acceptable length of interruption will depend on agreement between the investigator and the Sponsor.

Dose interruptions for reason(s) other than adverse events, such as surgical procedures, may be allowed with Sponsor approval. The acceptable length of interruption will depend on agreement between the investigator and the Sponsor.

### 10.1.6 Management of Atezolizumab/Placebo-Specific Adverse Events

Toxicities associated or possibly associated with atezolizumab/placebo treatment should be managed according to standard medical practice. Additional tests, such as autoimmune serology or biopsies, may be used to determine a possible immunogenic etiology.

Although most immune-mediated adverse events observed with immunomodulatory agents have been mild and self-limiting, such events should be recognized early and treated promptly to avoid potential major complications. Discontinuation of atezolizumab/placebo may not have an immediate therapeutic effect and, in severe cases, immune-mediated toxicities may require acute management with topical corticosteroids, systemic corticosteroids, or other immunosuppressive agents.

The primary approach to Grade 1−2 immune-mediated adverse events is supportive and symptomatic care with continued treatment with atezolizumab; for higher-grade immune-mediated adverse events, atezolizumab/placebo should be held and oral/parenteral steroids administered. Recurrent Grade 2 immune-mediated adverse events may also mandate holding atezolizumab/placebo or the use of steroids. Consideration for benefit-risk balance should be made by the investigator, with consideration of the totality of information as it pertains to the

nature of the toxicity and the degree of clinical benefit a given patient may be experiencing prior to further administration of atezolizumab/placebo. Atezolizumab/placebo should be permanently discontinued in patients with life-threatening immune-mediated adverse events.

See the atezolizumab Investigator's Brochure for details on management of gastrointestinal, dermatologic, endocrine, pulmonary toxicity, hepatotoxicity, potential pancreatic or eye toxicity, other immune-mediated adverse events and infusion-related reaction.

Systemic immune activation (SIA) is a rare condition characterized by an excessive immune response. Given the mechanism of action of atezolizumab, SIA is considered a potential risk when given in combination with other immunomodulating agents. SIA should be included in the differential diagnosis for patients who, in the absence of an alternative etiology, develop a sepsis-like syndrome after administration of atezolizumab, and the initial evaluation should include the following:

- CBC with peripheral smear
- PT, PTT, fibrinogen, and D-dimer
- Ferritin
- Triglycerides
- AST, ALT, and total bilirubin
- LDH
- Complete neurologic and abdominal examination (assess for hepatosplenomegaly)

If SIA is still suspected after the initial evaluation, contact the Sponsor for additional recommendations.

### 10.1.7 Dose-modification of chemotherapy

Pegylated liposomal doxorubicin will be administered in accordance with standard procedures and established practice at the study hospitals, including criteria for hematological counts. Cardiac function will be monitored if clinically indicated and according to routine practice. Dose reduction and delay of treatment is allowed when performed in accordance with standard practice and in line with the guidelines given in the Pegylated Liposomal Doxorubicin Product Information, as listed at ema.europa.eu. This includes guidelines on the management of stomatitis, palmar–plantar erythrodysesthesia and haematological toxicity.

The following adjustments to the standard guidelines apply:

- The administration of pegylated liposomal doxorubicin is withheld until a lymphopenia resolves to lymphocyte count $\geq 0.3 \times 10^9$/L and neutropenia resolves to grade $\leq 2$, i.e. neutrophil count $\geq 1.0 \times 10^9$/L.
- For grade 2 neutropenia or a lymphocyte count $< 0.8 \times 10^9$/L, the dose of pegylated liposomal doxorubicin should be reduced to 15mg/m$^2$
- Pegylated liposomal doxorubicin may be reduced in dose by up to 25%, i.e. to 15mg/m$^2$, or paused with up to two weeks when considered necessary by the investigator. If the investigator considers that a more extended pause is necessary, the investigator should

consult a member of the Study Leadership. Dose reductions below 15mg/$m^2$ are not allowed.

Metronomic cyclophosphamide, as used in the study, is expected to be well tolerated. If considered necessary by the investigator, the drug may be omitted for up to two weeks. If the investigator considers that a more extended pause is necessary, the investigator should consult a member of the Study Leadership.

## 10.2 Safety parameters and definitions

Safety assessments will consist of monitoring and recording of adverse events (including serious adverse events and non-serious adverse events of special interest), performing protocol-specified safety laboratory assessments, measuring protocol-specified vital signs, and conducting other protocol-specified tests that are deemed critical to the safety evaluation of the study.

Certain types of events require immediate reporting to the Sponsor, as outlined in Section 10.2.3.

### 10.2.1 Adverse Events

According to the ICH guideline for Good Clinical Practice, an adverse event is any untoward medical occurrence in a clinical investigation subject administered a pharmaceutical product, regardless of causal attribution. An adverse event can therefore be any of the following:

- Any unfavorable and unintended sign (including an abnormal laboratory finding), symptom, or disease temporally associated with the use of a medicinal product, whether or not considered related to the medicinal product
- Any new disease or exacerbation of an existing disease (a worsening in the character, frequency, or severity of a known condition), except events that are clearly consistent with the expected pattern of progression of the underlying disease
- Recurrence of an intermittent medical condition (e.g., headache) not present at baseline
- Any deterioration in a laboratory value or other clinical test (e.g., ECG, X-ray) that is associated with symptoms or leads to a change in study treatment or concomitant treatment or discontinuation from study drug
- Adverse events that are related to a protocol-mandated intervention, including those that occur prior to assignment of study treatment (e.g., screening invasive procedures such as biopsies)

### 10.2.2 Serious Adverse Events (Immediately Reportable to the Sponsor)

A serious adverse event is any adverse event that meets any of the following criteria:

- Fatal (i.e., the adverse event actually causes or leads to death)
- Life threatening (i.e., the adverse event, in the view of the investigator, places the patient at immediate risk of death) This does not include any adverse event that had it occurred in a more severe form or was allowed to continue might have caused death
- Requires or prolongs inpatient hospitalization
- Results in persistent or significant disability/incapacity (i.e., the adverse event results in substantial disruption of the patient’s ability to conduct normal life functions)

- Congenital anomaly/birth defect in a neonate/infant born to a mother exposed to study drug
- Significant medical event in the investigator's judgment (e.g., may jeopardize the patient or may require medical/surgical intervention to prevent one of the outcomes listed above)

The terms “severe” and “serious” are not synonymous. Severity refers to the intensity of an adverse event (rated as mild, moderate, or severe or according to NCI CTCAE criteria); the event itself may be of relatively minor medical significance (such as severe headache without any further findings).

Severity and seriousness need to be independently assessed for each adverse event recorded on the CRF.

Serious adverse events are required to be reported by the investigator to the Sponsor immediately (i.e., no more than 24 hours after learning of the event; see Section 10.3.1 for reporting instructions).

### 10.2.3 Suspected Unexpected Serious Adverse Reactions (Immediately Reportable to the Sponsor)

Suspected Unexpected Serious Adverse Reactions (SUSARs) are defined as Serious Adverse Drug Reactions that are not listed as expected in the Reference Safety Information for the IMP (last updated IB for atezolizumab; last updated SmPC for PLD and cyclophosphamide). In the atezolizumab IB version 11, this information is found in Table 41.

### 10.2.4 Adverse Events of Special Interest (Immediately Reportable to the Sponsor)

Adverse events of special interest are required to be reported by the investigator to the Sponsor immediately (i.e., no more than 24 hours after learning of the event). Adverse events of special interest for this study include the following conditions which may be suggestive of an autoimmune disorder:

- Immune related Pneumonitis
- Immune related Colitis
- Immune related adrenal insufficiency
- Immune related Hepatitis
- Immune related Hypothyroidism
- Immune related Hyperthyroidism
- Immune related Guillain-Barre Syndrome,
- Immune related Myasthenia Gravis,
- Immune related Meningoencephalitis
- Infusion related reactions (only events occurring on the same day as an Atezolizumab reaction should be identified)
- Immune related Pancreatitis
- Immune related Diabetes Mellitus
- Immune related Nephritis

- Immune related Myositis
- Ocular inflammatory toxicity – uveitis, ulcerative keratitis, episcleritis, optic neuritis
- Severe cutaneous reactions (e.g., Stevens-Johnson syndrome, dermatitis bullous, toxic epidermal necrolysis
- Rhabdomyolysis (only events occurring on the same day as an Atezolizumab reaction should be identified)
- Systemic Immune Activation (SIA) (only events occurring on the same day as an Atezolizumab reaction should be identified)
- Immune related Hypophysitis
- Immune related Myocardatis

In addition to the AESIs noted above the following events also requires immediate reporting:

- Events suggestive of hypersensitivity, cytokine release, influenza-like illness, systemic inflammatory response syndrome (SIRS), or infusion-reaction syndromes
- Cases of potential drug-induced liver injury that include an elevated ALT or AST in combination with either an elevated bilirubin or clinical jaundice, as defined by Hy's law (ALT > 3x ULN + bilirubin > 2x ULN)
- Suspected transmission of an infectious agent by the study drug, as defined below: Any organism, virus, or infectious particle (e.g., prion protein transmitting transmissible spongiform encephalopathy), pathogenic or non-pathogenic, is considered an infectious agent. A transmission of an infectious agent may be suspected from clinical symptoms or laboratory findings that indicate an infection in a patient exposed to a medicinal product. This term applies only when a contamination of the study drug is suspected

### 10.2.5 Procedures for recording of adverse events

#### 10.2.5.1 Diagnosis versus Signs and Symptoms

**Infusion-Related Reactions**
AEs that occur during or within 24 hours after study drug infusion should be captured as individual signs and symptoms rather than a diagnosis of allergic reaction or infusion reaction.

**Other Adverse Events**
For AEs other than infusion-related reactions, a diagnosis (if known) should be recorded on the Adverse Event eCRF rather than individual signs and symptoms (e.g., record only liver failure or hepatitis rather than jaundice and elevated transaminases). However, if a constellation of signs and/or symptoms cannot be medically characterized as a single diagnosis or syndrome at the time of reporting, each individual event should be recorded on the Adverse Event eCRF. If a diagnosis is subsequently established, all previously reported AEs based on signs and symptoms should be nullified and replaced by one AE report based on the single diagnosis, with a starting date that corresponds to the starting date of the first symptom of the eventual diagnosis.

#### 10.2.5.2 Adverse Events Occurring Secondary to Other Events

In general, AEs occurring secondary to other events (e.g., cascade events or clinical sequelae) should be identified by their primary cause. For example, if severe diarrhea is known to have resulted in dehydration, it is sufficient to record only diarrhea as an AE or SAE on the eCRF. However, medically significant AEs occurring secondary to an initiating event that are separated in time should be recorded as independent events on the eCRF. For example, if a severe gastrointestinal hemorrhage leads to renal failure, both events should be recorded separately on the eCRF.

#### 10.2.5.3 Persistent or Recurrent Adverse Events

A persistent AE is one that extends continuously, without resolution, between patient evaluation time points. Such events should only be recorded once on the Adverse Event eCRF. The initial severity of the event should be recorded, and the severity should be updated to reflect the most extreme severity any time the event worsens. If the event becomes serious, the Adverse Event eCRF should be updated to reflect this.

A recurrent AE is one that resolves between patient evaluation time points and subsequently recurs. Each recurrence of an AE should be recorded separately on the Adverse Event eCRF.

#### 10.2.5.4 Abnormal Laboratory Values

Only clinically significant laboratory abnormalities that require active management will be recorded as AEs or SAEs on the eCRF (e.g., abnormalities that require study drug dose modification, discontinuation of study treatment, more frequent follow-up assessments, further diagnostic investigation, etc.)

If the clinically significant laboratory abnormality is a sign of a disease or syndrome (e.g. bilirubin 5 × ULN of normal associated with cholecystitis), only the diagnosis (e.g., cholecystitis) needs to be recorded on the AE eCRF.

If the clinically significant laboratory abnormality is not a sign of a disease or syndrome, the abnormality itself should be recorded as an AE or SAE on the eCRF. If the laboratory abnormality can be characterized by a precise clinical term, the clinical term should be recorded as the AE or SAE.

Observations of the same clinically significant laboratory abnormality from visit to visit should not be repeatedly recorded as AEs or SAEs on the eCRF, unless their severity, seriousness, or etiology changes.

#### 10.2.5.5 Preexisting Medical Conditions

A preexisting medical condition is one that is present at the screening visit for this study. Such conditions should be recorded in the eCRF. A preexisting medical condition should be recorded as an AE only if the frequency, severity, or character of the condition worsens during the study.

#### 10.2.5.6 Lack of Efficacy or Worsening of Breast Cancer

Events that are clearly consistent with the expected pattern of progression of the underlying disease should not be recorded as AEs.

#### 10.2.5.7 Hospitalization or Prolonged Hospitalization

Any AE that results in hospitalization or prolonged hospitalization should be documented and reported as a SAE unless specifically instructed otherwise in this protocol. There are some hospitalization scenarios that do not require reporting as an SAE when there is no occurrence of an AE. These scenarios include a planned hospitalization or prolonged hospitalization to:

- Perform an efficacy measurement for the study
- Undergo a diagnostic or elective surgical procedure for a preexisting medical condition that has not changed

## 10.3 Immediate reporting requirements from investigator to sponsor

Certain events require immediate reporting to allow the Sponsor to take appropriate measures to address potential new risks in a clinical trial. The investigator must report such events to the Sponsor immediately; under no circumstances should reporting take place more than 24 hours after the investigator learns of the event. The following is a list of events that the investigator must report to the Sponsor within 24 hours after learning of the event, regardless of relationship to study drug:

- Serious adverse events (section 10.2.2)
- Non-serious adverse events of special interest (section 10.2.4)
- Pregnancies (section 10.3.2)

### 10.3.1 Reporting Requirements for Serious Adverse Events and Non-Serious Adverse Events of Special Interest

#### 10.3.1.1 Events that Occur Prior to Study Drug Initiation

After informed consent has been obtained but prior to initiation of study drug, only serious adverse events caused by a protocol-mandated intervention should be reported. The Serious Adverse Event should be reported to Sponsor immediately (i.e., no more than 24 hours after learning of the event.

#### 10.3.1.2 Events that Occur After Study Drug Initiation

After initiation of study drug, serious adverse events and non-serious adverse events of special interest will be reported until 30 days after the last dose of study drug or until initiation of another anti-cancer therapy, whichever occurs first. Investigators should record all case details that can be gathered immediately (i.e., within 24 hours after learning of the event) and forward these to the Sponsor.

Instructions for reporting post-study adverse events are provided in Section 10.5.

### 10.3.2 Reporting Requirements for Pregnancies

#### 10.3.2.1 Pregnancies in Female Patients

Female patients of childbearing potential will be instructed to immediately inform the investigator if they become pregnant during the study or within 90 days after the last dose of study drug. A Pregnancy Report Form should be completed by the investigator immediately (i.e., no more than 24 hours after learning of the pregnancy) and reported to the Sponsor. Pregnancies should not be recorded on the Adverse Event CRF. The investigator should discontinue study drug and counsel the patient, discussing the risks of continuing the pregnancy and the possible effects on the fetus. Monitoring of the patient should continue until the conclusion of the pregnancy. Any serious adverse events associated with the pregnancy (e.g., an event in the fetus, an event in the mother during or after the pregnancy, or a congenital anomaly/birth defect in the child) should be reported on the Adverse Event CRF. In addition, the investigator will update the Pregnancy Report Form when updated information on the course and outcome of the pregnancy becomes available.

#### 10.3.2.2 Pregnancies in Female Partners of Male Patients

Male patients will be instructed through the Informed Consent Form to immediately inform the investigator if their partner becomes pregnant during the study or within 90 days after the last dose of study medication. A Pregnancy Report Form should be completed by the investigator immediately (i.e., no more than 24 hours after learning of the pregnancy) and submitted to Sponsor. Attempts should be made to collect and report details of the course and outcome of any pregnancy in the partner of a male patient exposed to study drug. The pregnant partner will need to sign an Authorization for Use and Disclosure of Pregnancy Health Information to allow for follow-up on her pregnancy. Once the authorization has been signed, the investigator will update the Pregnancy Report Form with additional information on the course and outcome of the pregnancy. An investigator who is contacted by the male patient or his pregnant partner may provide information on the risks of the pregnancy and the possible effects on the fetus to support an informed decision in cooperation with the treating physician and/or obstetrician.

#### 10.3.2.3 Abortions

Any spontaneous abortion should be classified as a serious adverse event (as the Sponsor considers spontaneous abortions to be medically significant events), recorded on the Adverse Event CRF, and reported to the Sponsor immediately (i.e., no more than 24 hours after learning of the event).

#### 10.3.2.4 Congenital Anomalies/Birth Defects

Any congenital anomaly/birth defect in a child born to a female patient exposed to study drug or the female partner of a male patient exposed to study drug should be classified as a serious adverse event, recorded on the Adverse Event CRF, and reported to the Sponsor immediately (i.e., no more than 24 hours after learning of the event).

## 10.4 Follow-up of patients after adverse events

### 10.4.1 Investigator Follow-Up

During the study period, resolution of adverse events (with dates) should be documented on the Adverse Event CRF and in the patient's medical record to facilitate source data verification. If, after follow-up, return to baseline status or stabilization cannot be established, an explanation should be recorded on the Adverse Event CRF. Every effort should be made to follow all serious adverse events considered related to study drug or trial-related procedures until a final outcome can be reported. All pregnancies reported during the study should be followed until pregnancy outcome.

### 10.4.2 Sponsor Follow-Up

For serious adverse events, non-serious adverse events of special interest, and pregnancies, the Sponsor or a designee may follow up by telephone, fax, electronic mail, and/or a monitoring visit to obtain additional case details and outcome information (e.g., from hospital discharge summaries, consultant reports, autopsy reports) in order to perform an independent medical assessment of the reported case.

## 10.5 Post-study adverse events

At the treatment discontinuation visit, the investigator should instruct each patient to report to the investigator any subsequent adverse events that the patient's personal physician believes could be related to prior study drug treatment or study procedures.
Investigators should notify the Sponsor of any serious adverse events and adverse events of special interest that are believed to be related to prior drug treatment or study procedures that occur at any time after a patient has discontinued study participation. The Sponsor should also be notified if the investigator becomes aware of the development of cancer or a congenital anomaly/birth defect in a subsequently conceived offspring of a patient who participated in this study.

## 10.6 Sponsor Responsibility for Reporting Adverse Events

All Adverse Events will be reported to Competent Authorities and Investigators in accordance with applicable national laws and regulations.

Suspected Unexpected Serious Adverse Reactions (SUSARs) will be reported to the Competent Authority according to national regulations. The following timelines should be followed:

- The sponsor will ensure that all relevant information about SUSARs that are fatal or life-threatening is recorded and reported as soon as possible to the Competent Authority concerned in any case no later than seven (7) days after knowledge by the sponsor of such a case, and that relevant follow-up information is subsequently communicated within an additional eight (8) days.
- All other SUSARs will be reported to the Competent Authority concerned as soon as possible but within a maximum of fifteen (15) days of first knowledge by the sponsor.

SUSARs will be reported using the CIOMS form since Oslo University Hospital (sponsor) is not connected to EudraVigilance. In addition Roche will get a copy of all reports. Details will be provided in the Safety Data exchange agreement that will be signed prior study start.

## 11.0 COLLATERAL RESEARCH

An extensive research program will be conducted. The patient informed consent form will allow for performing biomarker analyses, immunological studies, gene profiling and studies of tumor evolution/heterogeneity during treatment, as well as comparison with data/material from other studies.

### 11.1 Biobanking

Please see the study flow chart for information on the time points for collection of biopsies, peripheral blood and urine and faeces sample.

- Tumor biopsies collected pre, during and post therapy (time of progression). If sufficient tissue is available, three tumor biopsies will be collected :
    - FFPE tissue
    - Fresh tumor cells/ tumor infiltrating lymphocytes frozen as cell suspension
    - Snap-frozen tumor biopsies
- Before therapy, FFPE tissue for diagnosis must also be prepared, if this is not already available as archive material from metastatic lesion
- Archival biopsies and blood samples taken before study entry will be collected, for subjects that have given consent to this in the Informed Consent Form (ICF). This consent is included in the Norwegian ICF version 2.4 and later, and the Danish ICF version 5.2 or later. For subjects at Norwegian study sites who have signed an earlier version of the ICF, and who died before they could be presented an updated version, archival material may be collected, as approved by the Norwegian National Committee for Medical and Health Research Ethics. For subjects at Danish study sites, archival material will only be collected for patients who have given explicit consent.
- Peripheral blood samples collected pre-, during and post-therapy:
    - Peripheral blood mononuclear cells, processed with gradient centrifugation and frozen on liquid nitrogen
    - Plasma/serum, separated and frozen.
    - Circulating tumor cells (only if sufficient resources)
- Urine samples collected pre-, during and post-therapy
- Feces sample

Biobank samples will be stored in an approved research biobank at Oslo university hospital that is currently approved until 31 December 2040. Biobank samples collected at study sites other than Oslo university hospital may be stored in a research biobank at the study site for up to 24 months. These local research biobanks in Denmark will be terminated at the end of the study period, currently approved until 30 June 2022.

### 11.2 Plans for translational research addressing exploratory/secondary endpoints

The list below does not represent a mandatory list of assays that are to be performed, but provides an overview of the current plans. The prioritization of assays will be subject to review during the trial. Additional assays may be performed, reflecting ongoing developments in the field. Some of these assays will only be performed on selected patients, due to cost and work load.

- Gene profiling with selected panels (e.g. PAM50, customized immune-gene and tumor gene panels)
- Pathology/immunohistochemistry (incl. PD-L1 and markers for leucocyte subpopulations)
- DNA sequencing of tumor and normal PBMCs (mutations, mutation load, HLA types/loss, SNPs)(20)
- RNA transcriptome sequencing of tumor from selected patients (gene expression profiles, neoantigens)
- Serum biomarkers/soluble biopsies, including
    - circulating DNA
    - circulating micro RNA(21)
    - Bioplex cytokine- and MMP panels
- Characterization of cell suspensions from tumor and peripheral blood:
    - CYTOF (investigating subpopulations/heterogeneity/evolution, both within tumor cells, immune cells and other stromal cells; relate CYTOF data to gene profiling and mRNA expression)
    - Flow cytometry, including the use of panels for regulatory T cells and myeloid suppressor cells(22)
- Functional T cell assays, incl. ELISPOT, proliferation, Bioplex cytokine profiling, multimers (23)
- Test of T cell reactivity against the individual spectrum of tumor antigens in each patient´s tumor, identified through tumor sequencing and epitope prediction
- Tumor gene profiling, for monitoring tumor evolution during treatment(20, 24)
- Circulating tumor cells in peripheral blood(25, 26)

## 12.0 STATISTICS

### 12.1 Statistical analyses

The ITT population is defined as a full analysis set (FAS). The FAS is defined as all randomized patients that have started therapy with at least one of the IMPs. The safety will be evaluated in the FAS.

The PP population is defined as all randomized patients that have received/completed at least 4 doses of atezolizumab/placebo and 3 doses with PLD. Patients that cannot be evaluated for tumor

response or receive ≤3 doses of atezolizumab/placebo or ≤ 2 doses with PLD will not be considered evaluable per protocol, but will be included in the FAS analysis.

The primary efficacy analyses will be performed on the PP population and the PD-L1 positive PP population. Secondary efficacy analyses will be performed also on the PD-L1 negative subpopulation and on the FAS.

The primary efficacy analysis will be a descriptive analysis of progression free survival (PFS) in the atezolizumab arm, compared to the control group. PFS is defined as the time from randomization to the occurrence of disease progression, as determined by investigators from tumor assessments per immune-modified RECIST (iRECIST), or death from any cause, whichever occurs first. Data for patients without disease progression or death will be censored at the last tumor assessment date. Data for patients with a PFS event who missed two or more assessments scheduled immediately prior to the date of the PFS event will be censored at the last tumor assessment prior to the missed visits. If no tumor assessment was performed after randomization, data will be censored at the date of randomization +1 day. Clinical deterioration without objective radiological evidence will not be considered as documented disease progression. The HR for disease progression or death (arm B versus arm A) will be estimated using a Cox proportional hazards model. The CI for the HR will be provided. Kaplan-Meier methodology will be used to estimate the median PFS for each treatment arm, and Kaplan-Meier curves with CI will be produced.

Overall survival (OS) will be calculated from the time of randomization until death. Patients alive at the time of data analysis will be treated as censored. The HR for OS (arm B versus arm A) will be estimated using a Cox proportional hazards model. The CI for the HR will be provided. Kaplan-Meier methodology will be used to estimate the median OS for each treatment arm, and Kaplan-Meier curves with CI will be produced.
Further, a descriptive analysis of demographics, medical history, and clinical data will be performed.

The unblinding and primary PFS analysis was planned to be performed when 90% PFS events were reached in the PP population. This plan has been changed due to the availability of atezolizumab as standard therapy for PD-L1+ mTNBC, which means that nearly no PD-L1+ TNBC patients can be enrolled. Due to the reduced rate of enrolment, we have decided to stop the enrolment of new patients on 31 December 2021. The timing of the unblinding and analysis is detailed in the Statistical Analysis Plan (SAP).

The efficacy analyses are descriptive and no formal hypothesis tests will be performed. The α-levels will therefore not be corrected for multiple testing, including testing at repeated time points.

The following pre-defined factors will be studied in the statistical analyses:

- Tumor PD-L1 status

- Disease-free interval: Less than 24 months versus >24 months between end of adjuvant chemotherapy or surgery, whichever was last, and relapse (most TNBC patients receive adjuvant chemo for 6 months before/after radical surgery; a short disease-free interval suggests aggressive disease)
- Prior chemotherapy against metastatic disease (no previous chemo vs. previous chemo).
- Prior chemotherapy given in the neoadjuvant/adjuvant/metastatic setting, versus no prior chemotherapy against BC
- Site(s) of metastases
- Tumor mutational burden
- Molecular breast cancer profile and immune gene profile

Exploratory analyses will be carried out to evaluate the data of the immunological and molecular analyses (e.g. biomarker studies) carried out. The statistical analyses will be dependent on the biological factors investigated and the analysis methodology used, and will be defined separately for each molecular study.

## 12.2 Statistical considerations regarding sample size and randomization ratio

The phase II study cannot be powered to demonstrate a statistically significant ($p<0.05$) clinical effect. If the study suggests acceptable toxicity and potential clinical benefit, a larger randomized study will be warranted. We planned to conduct a phase II study with 75 patients in the PP population (45 patients in the atezo-chemo arm, 30 patients in the chemo-only arm). This number has since been reduced, due to changes in standard treatment of TNBC (see above). The number of 75 patients and the randomization ratio of 3:2 were based on the following considerations:

- **Expected PFS for the control group, receiving only chemotherapy:**

| Months | Progression-free proportion |
|---|---|
| 4 | 50% |
| 6 | 35% |
| 12 | 10% |
| 15-18 | 5% |

The PFS estimate was based on literature and was decided upon in a meeting of the OUS Steering Group for breast cancer studies

- **Statistical power calculations for the primary endpoint PFS**
  A two-sided hypothesis test was performed with a 10% significance level and a desired power of 80%. The test was NOT performed to define a number of patients where a significant clinical effect ($p<0.05$) could be determined, as this is not the aim of a phase II trial. Rather, the calculation was done to illustrate what we can expect to observe within a realistic number of patients for a phase II trial and to inform the choice of randomization ratio. For this purpose, we chose a significance level of 10% and performed the test for a sample size of 60, 75 and 80 patients. The calculation for 75 patients is given below (Table 4).

| Randomization ratio | PFS 1 | PFS 2 | HR |
|---|---|---|---|
| 1:1 | 0.05 | 0.208 | 0.52 |
| 2:3 | 0.05 | 0.198 | 0.54 |
| 1:2 | 0.05 | 0.198 | 0.54 |

**Table 4: Statistical power with 75 patients**
A two-sided hypothesis test was performed with a 10% significance level and a desired power of 80%. PFS 1 is the PFS probability in the control group at the end of the study. PFS 2 is the PFS probability in the experimental group. HR = hazard ratio, effect size of the experimental to the control group

The power calculation indicates that a **randomization ratio of 2:3** or 1:2 is preferable to 1:1. A ratio of 2:3 is chosen rather than 1:2 in order to increase the statistical power for collateral research analyses.

The biomarker research program aims at identifying which patients benefit from treatment and may inform the design of a subsequent randomized trial. The suggested number of patients will allow for meaningful statistical comparisons of biological/immunological data, and comparison with data from our previous studies and the OUS breast cancer biobank.

## 13.0 EHICAL CONSIDERATIONS

### 13.1 Compliance with Laws and Regulations

This study will be conducted in full conformance with the ICH E6 guideline for Good Clinical Practice and the principles of the Declaration of Helsinki, or the laws and regulations of the country in which the research is conducted, whichever affords the greater protection to the individual. The study will comply with the requirements of the ICH E2A guideline (Clinical Safety Data Management: Definitions and Standards for Expedited Reporting), in addition with the E.U. Clinical Trial Directive (2001/20/EC).

### 13.2 Informed Consent

The Sponsor’s sample Informed Consent will be provided to each site.

The Informed Consent Form will contain a separate section that addresses the use of remaining mandatory samples for optional exploratory research. The investigator or authorized designee will explain to each patient the objectives of the exploratory research. Patients will be told that they are free to refuse to participate and may withdraw their specimens at any time and for any reason during the storage period. A separate, specific signature will be required to document a patient's agreement to allow any remaining specimens to be used for exploratory research. Patients who decline to participate will not provide a separate signature.

The Consent Forms must be signed and dated by the patient or the patient's legally clinical records for each patient shall document the informed consent process and that written informed consent was obtained prior to participation in the study.

The Consent Forms should be revised whenever there are changes to study procedures or when new information becomes available that may affect the willingness of the patient to participate. The final revised IRB/EC-approved Consent Forms must be provided to the Sponsor for health authority submission purposes.

Patients must be re-consented to the most current version of the Consent Forms (or to a significant new information/findings addendum in accordance with applicable laws and IRB/EC policy) during their participation in the study. For any updated or revised Consent Forms, the case history or clinical records for each patient shall document the informed consent process and that written informed consent was obtained using the updated/revised Consent Forms for continued participation in the study.

A copy of each signed Consent Form must be provided to the patient or the patient's legally authorized representative. All signed and dated Consent Forms must remain in each patient's study file or in the site file and must be available for verification by study monitors at any time.

### 13.3 Ethics Committee

This protocol, the Informed Consent Forms, any information to be given to the patient, and relevant supporting information will be submitted to the Ethics Committee, reviewed and approved according to EU and national regulations before the study is initiated. This is also valid for any amendments and/or a new version of the study protocol (Amended Protocol).

### 13.4 Confidentiality

The Sponsor maintains confidentiality standards by coding each patient enrolled in the study through assignment of a unique patient identification number. This means that patient names are not included in data sets.

Patient medical information obtained by this study is confidential and may be disclosed to third parties only as permitted by the Informed Consent Form (or separate authorization for use and disclosure of personal health information) signed by the patient, unless permitted or required by law.

Medical information may be given to a patient's personal physician or other appropriate medical personnel responsible for the patient's welfare, for treatment purposes.

Data generated by this study must be available for inspection upon request by representatives of the Competent Authority, Sponsor, monitors, representatives, and collaborators, and the EC for each study site, as appropriate.

# 14.0 ADMINISTRATIVE AND REGULATORY DETAILS

## 14.1 Study Documentation

The investigator must maintain adequate and accurate records to enable the conduct of the study to be fully documented, including but not limited to the protocol, protocol amendments, Informed Consent Forms, and documentation of EC and governmental approval.

## 14.2 Protocol Adherence

Investigators ascertain they will apply due diligence to avoid protocol deviations.
All significant protocol deviations will be recorded and reported in the Clinical Study Report (CSR).

## 14.3 Monitoring

The investigator/site will be visited on a regular basis by the Clinical Study Monitor, who will assess compliance with the trial protocol and general principles of Good Clinical Practice. The monitor will review the relevant CRFs for accuracy and completeness and will ask the site staff to adjust any discrepancies as required.

Sponsor’s representatives (e.g. monitors, auditors) and/or competent authorities will be allowed access to source data for source data verification in which case a review of those parts of the hospital records relevant to the study may be required.

## 14.4 Audit and Inspections

Authorized representatives of a Competent Authority and Ethics Committee may visit the centers to perform inspections, including source data verification. Likewise the representatives from sponsor may visit the centers to perform an audit. The purpose of an audit or inspection is to systematically and independently examine all study-related activities and documents to determine whether these activities were conducted, and data were recorded, analyzed, and accurately reported according to the protocol, Good Clinical Practice (ICH GCP), and any applicable regulatory requirements. The principal investigator at each study site will ensure that the inspectors and auditors will be provided with access to source data/documents.

## 14.5 Data Management and Transfer

The Investigator or qualified designee is responsible for recording and verifying the accuracy of subject data. Detailed information regarding Data Management procedures for this protocol will be provided separately.
Data and biological material may be transferred to collaborators in other countries. The transfer of data and biological material to collaborators within and outside of the European Union / European Economic Area will be conducted in accordance with the Data Protection Act and the General Data Protection Regulation (EU) 2016/679, Chapter V.

### 14.6 Publication Policy

Upon study completion and finalization of the study report the results of this study will either be submitted for publication and/or posted in a publicly assessable database of clinical study results. The results of this study will also be submitted to the Competent Authority and the Ethics Committee according to EU and national regulations.

All personnel who have contributed significantly with the planning and performance of the study (Vancouver convention 1988) may be included in the list of authors.

## 15.0 LIST OF REFERENCES


1. Bianchini G, Balko JM, Mayer IA, Sanders ME, Gianni L. Triple-negative breast cancer: challenges and opportunities of a heterogeneous disease. Nat Rev Clin Oncol. 2016;13(11):674-90.
2. Schumacher TN, Schreiber RD. Neoantigens in cancer immunotherapy. Science. 2015;348(6230):69-74.
3. Bezu L, Gomes-de-Silva LC, Dewitte H, Breckpot K, Fucikova J, Spisek R, et al. Combinatorial strategies for the induction of immunogenic cell death. Frontiers in immunology. 2015;6:187.
4. Apetoh L, Ghiringhelli F, Tesniere A, Obeid M, Ortiz C, Criollo A, et al. Toll-like receptor 4-dependent contribution of the immune system to anticancer chemotherapy and radiotherapy. Nat Med. 2007;13(9):1050-9.
5. Sistigu A, Yamazaki T, Vacchelli E, Chaba K, Enot DP, Adam J, et al. Cancer cell-autonomous contribution of type I interferon signaling to the efficacy of chemotherapy. Nat Med. 2014;20(11):1301-9.
6. Kroemer G, Senovilla L, Galluzzi L, Andre F, Zitvogel L. Natural and therapy-induced immunosurveillance in breast cancer. Nat Med. 2015;21(10):1128-38.
7. Pfirschke C, Engblom C, Rickelt S, Cortez-Retamozo V, Garris C, Pucci F, et al. Immunogenic Chemotherapy Sensitizes Tumors to Checkpoint Blockade Therapy. Immunity. 2016;44(2):343-54.
8. Ghiringhelli F, Menard C, Puig PE, Ladoire S, Roux S, Martin F, et al. Metronomic cyclophosphamide regimen selectively depletes CD4+CD25+ regulatory T cells and restores T and NK effector functions in end stage cancer patients. Cancer Immunol Immunother. 2007;56(5):641-8.
9. Schmid P, Adams S, Rugo HS, Schneeweiss A, Barrios CH, Iwata H, et al. Atezolizumab and Nab-Paclitaxel in Advanced Triple-Negative Breast Cancer. N Engl J Med. 2018;379(22):2108-21.
10. Munzone E, Colleoni M. Clinical overview of metronomic chemotherapy in breast cancer. Nat Rev Clin Oncol. 2015;12(11):631-44.
11. Zhao M, Ding XF, Shen JY, Zhang XP, Ding XW, Xu B. Use of liposomal doxorubicin for adjuvant chemotherapy of breast cancer in clinical practice. Journal of Zhejiang University Science B. 2017;18(1):15-26.
12. Jehn CF, Hemmati P, Lehenbauer-Dehm S, Kummel S, Flath B, Schmid P. Biweekly Pegylated Liposomal Doxorubicin (Caelyx) in Heavily Pretreated Metastatic Breast Cancer: A Phase 2 Study. Clin Breast Cancer. 2016;16(6):514-9.
13. Rossi D, Baldelli AM, Casadei V, Fedeli SL, Alessandroni P, Catalano V, et al. Neoadjuvant chemotherapy with low dose of pegylated liposomal doxorubicin plus weekly paclitaxel in operable and locally advanced breast cancer. Anti-cancer drugs. 2008;19(7):733-7.
14. Dellapasqua S, Mazza M, Rosa D, Ghisini R, Scarano E, Torrisi R, et al. Pegylated liposomal doxorubicin in combination with low-dose metronomic cyclophosphamide as preoperative treatment for patients with locally advanced breast cancer. Breast. 2011;20(4):319-23.
15. Rau KM, Lin YC, Chen YY, Chen JS, Lee KD, Wang CH, et al. Pegylated liposomal doxorubicin (Lipo-Dox(R)) combined with cyclophosphamide and 5-fluorouracil is effective and

safe as salvage chemotherapy in taxane-treated metastatic breast cancer: an open-label, multi-center, non-comparative phase II study. BMC cancer. 2015;15:423.
16. Brahmer J, Reckamp KL, Baas P, Crino L, Eberhardt WE, Poddubskaya E, et al. Nivolumab versus Docetaxel in Advanced Squamous-Cell Non-Small-Cell Lung Cancer. N Engl J Med. 2015;373(2):123-35.
17. Robert C, Ribas A, Wolchok JD, Hodi FS, Hamid O, Kefford R, et al. Anti-programmed-death-receptor-1 treatment with pembrolizumab in ipilimumab-refractory advanced melanoma: a randomised dose-comparison cohort of a phase 1 trial. Lancet. 2014;384(9948):1109-17.
18. Chalder T, Berelowitz G, Pawlikowska T, Watts L, Wessely S, Wright D, et al. Development of a fatigue scale. J Psychosom Res. 1993;37(2):147-53.
19. Seymour L, Bogaerts J, Perrone A, Ford R, Schwartz LH, Mandrekar S, et al. iRECIST: guidelines for response criteria for use in trials testing immunotherapeutics. Lancet Oncol. 2017;18(3):e143-e52.
20. Kristensen VN, Lingjaerde OC, Russnes HG, Vollan HK, Frigessi A, Borresen-Dale AL. Principles and methods of integrative genomic analyses in cancer. Nat Rev Cancer. 2014;14(5):299-313.
21. Kleivi Sahlberg K, Bottai G, Naume B, Burwinkel B, Calin GA, Borresen-Dale AL, et al. A serum microRNA signature predicts tumor relapse and survival in triple-negative breast cancer patients. Clin Cancer Res. 2015;21(5):1207-14.
22. Hansen GL, Gaudernack G, Brunsvig PF, Cvancarova M, Kyte JA. Immunological factors influencing clinical outcome in lung cancer patients after telomerase peptide vaccination. Cancer Immunol Immunother. 2015;64(12):1609-21.
23. Kyte JA, Gaudernack G, Dueland S, Trachsel S, Julsrud L, Aamdal S. Telomerase Peptide Vaccination Combined with Temozolomide: A Clinical Trial in Stage IV Melanoma Patients. Clin Cancer Res. 2011;17(13):4568-80.
24. McGranahan N, Furness AJ, Rosenthal R, Ramskov S, Lyngaa R, Saini SK, et al. Clonal neoantigens elicit T cell immunoreactivity and sensitivity to immune checkpoint blockade. Science. 2016.
25. Naume B, Synnestvedt M, Falk RS, Wiedswang G, Weyde K, Risberg T, et al. Clinical outcome with correlation to disseminated tumor cell (DTC) status after DTC-guided secondary adjuvant treatment with docetaxel in early breast cancer. J Clin Oncol. 2014;32(34):3848-57.
26. Seeberg LT, Waage A, Brunborg C, Hugenschmidt H, Renolen A, Stav I, et al. Circulating tumor cells in patients with colorectal liver metastasis predict impaired survival. Ann Surg. 2015;261(1):164-71.

## 16.0 ABBREVIATIONS

| Abbreviation/Term | Definition |
|---|---|
| ACD | Acid Citrate Dextrose |
| AE | Adverse Event |
| AESI | Adverse Events of Special Interest |
| ALT | Alanine Aminotransferase |
| aPTT | activated Partial Thromboplastin Time |
| AST | Aspartate Aminotransferase |
| BML | Below Measurable Limit |
| BPI | Brief Pain Inventory |
| CA125 | Cancer Antigen 125 |
| CBC | Complete Blood Count |
| CEA | Carcinoembryonic Antigen |
| CNS | Central Nervous System |
| CR | Complete Response |
| CRF | Case Report Form (electronic/paper) |
| CRP | C-reactive Protein |
| CT | Computer Tomography |
| CTC | Circulating tumor cells |
| CTCAE | Common Terminology Criteria for Adverse Event |
| CTLA-4 | Cytotoxic T Lymphocyte Antigen 4 |
| CYTOF | CYtometry Time Of Flight |
| DNA | Deoxyribonucleic Acid |
| DOR | Duration of Objective Response |
| DR | Duration Of response |
| DRR | Durable tumor Response Rate |
| DRR | Durable response Rate |
| EC | Ethics Committee, synonymous to Institutional Review Board (IRB) and Independent Ethics Committee (IEC) |
| ECG | Electrocardiogram |
| ECOG | Eastern Cooperative Oncology Group |
| EMA | European Medicines Agency |
| EORTC | European Organization for Research and Treatment of Cancer |
| ER | Estrogen Receptor |
| ERC | European Research Council |
| EU | European Union |
| FPI | First Patient In |
| FQ | Fatigue Questionnaire |
| GP | General Practician |
| HER2 | Human Epidermal growth factor Receptor 2 |
| HLA | Human Leukocyte Antigen |

| | |
|---|---|
| HR | Hazard Ratio |
| IB | Investigator’s Brochure |
| ICH | International Conference on Harmonization |
| IHC | Immunohistochemistry |
| IMP | Investigational Medicinal Product |
| INR | International Normalized Ratio |
| IRB | Institutional Review Board |
| iRECIST | immune-modified Response Evaluation Criteria in Solid Tumors |
| ISH | In situ hybridization |
| iCPD | Confirmed progressive disease according to iRECIST |
| ITT | Intention To Treat |
| IUD | Intrauterine device |
| iUPD | Unconfirmed progressive disease according to iRECIST |
| IV | Intravenous |
| LDH | Lactate Dehydrogenase |
| LVEF | Left Ventricular Ejection Fraction |
| MCID | Minimally Clinically Important Difference |
| MDSC | Myeloid-derived Suppressor Cells |
| MMP | Matrix Metalloproteinase |
| MRI | Magnetic Resonance Imaging |
| NCI | National Cancer Institute |
| NRS | Numerical Rating Scale |
| ORR | Objective Response Rate |
| OS | Overall Survival |
| OUS | Oslo University Hospital |
| PBMC | Peripheral Blood Mononuclear Cell |
| PD | Progressive disease |
| PD-1 | Programmed Death 1 |
| PD-L1 | Programmed Death Ligand-1 |
| PFS | Progression-free Survival |
| PI | Principle Investigator |
| PP | Per Protocol |
| PR | Progesterone Receptor |
| PRO | Patient Reported Outcome |
| QLQ | Quality of Life Questionnaire |
| RNA | Ribonucleic Acid |
| SAE | Serious Adverse Event |
| SD | Stable Disease |
| SIA | Systemic Immune Activation |
| SLD | Sum of Longest Diameters |
| SMC | Safety Monitoring Committee |
| SmPC | Summary of Product Characteristics |
| SNP | Single Nucleotide Polymorphism |
| SUSAR | Suspected Unexpected Serious Adverse Reaction |
| TCR | T-Cell Receptor |

| | |
|---|---|
| TNBC | Triple Negative Breast Cancer |
| TNF | Tumor Necrosis Factor |
| TPA | Tissue Plasminogen Activator |
| TSH | Thyroid Stimulating Hormone |
| TTD | Time to Deterioration |
| ULN | Upper Limit of Normal |

**17.0 APPENDICES**

## 17.1 ECOG Performance Status

| Grade | Description |
|---|---|
| 0 | Normal activity. Fully active, able to carry on all pre-disease performance without restriction. |
| 1 | Symptoms, but ambulatory. Restricted in physically strenuous activity, but ambulatory and able to carry out work of a light or sedentary nature (e.g., light housework, office work). |
| 2 | In bed <50% of the time. Ambulatory and capable of all self-care, but unable to carry out any work activities. Up and about more than 50% of waking hours. |
| 3 | In bed >50% of the time. Capable of only limited self-care, confined to bed or chair more than 50% of waking hours. |
| 4 | 100% bedridden. Completely disabled. Cannot carry on any self-care. Totally confined to bed or chair. |
| 5 | Dead. |
| * As published in Am. J. Clin. Oncol.: *Oken, M.M., Creech, R.H., Tormey, D.C., Horton, J., Davis, T.E., McFadden, E.T., Carbone, P.P.: Toxicity And Response Criteria Of The Eastern Cooperative Oncology Group. Am J Clin Oncol 5:649-655, 1982.* The Eastern Cooperative Oncology Group, Robert Comis M.D., Group Chair. | |

## 17.2 Common Terminology Criteria for Adverse Events v 4.0

The descriptions and grading scales found in the revised NCI Common Terminology Criteria for Adverse Events (CTCAE) version 4.0 will be utilized for adverse event reporting. (http://ctep.cancer.gov/reporting/ctc.html).

## 17.3 Immune-modified Response Evaluation Criteria in Solid Tumors (iRECIST)

Conventional response criteria may not be adequate to characterize the anti-tumor activity of immunotherapeutic agents like atezolizumab, which can produce delayed responses that may be preceded by initial apparent radiological progression, including the appearance of new lesions. Therefore, immune-modified response criteria (iRECIST) have been developed that account for the possibility of pseudoprogression. .

An updated consensus guideline for immune-modified Response Evaluation Criteria in Solid Tumors, iRECIST, was published on behalf of the RECIST working group March 2017. This guideline (19) is to be used in the present protocol.

Tables 1 and 2 below show key points in iRECIST. Please see the published iRECIST guideline, with supplementary materials, for the complete criteria (19).

| | RECIST 1.1 | iRECIST |
|---|---|---|
| Definitions of measurable and non-measurable disease; numbers and site of target disease | Measurable lesions are ≥10 mm in diameter (≥15 mm for nodal lesions); maximum of five lesions (two per organ); all other disease is considered non-target (must be ≥10 mm in short axis for nodal disease) | No change from RECIST 1.1; however, new lesions are assessed as per RECIST 1.1 but are recorded separately on the case report form (but not included in the sum of lesions for target lesions identified at baseline) |
| Complete response, partial response, or stable disease | Cannot have met criteria for progression before complete response, partial response, or stable disease | Can have had iUPD (one or more instances), but not iCPD, before iCR, iPR, or iSD |
| Confirmation of complete response or partial response | Only required for non-randomised trials | As per RECIST 1.1 |
| Confirmation of stable disease | Not required | As per RECIST 1.1 |
| New lesions | Result in progression; recorded but not measured | Results in iUPD but iCPD is only assigned on the basis of this category if at next assessment additional new lesions appear or an increase in size of new lesions is seen (≥5 mm for sum of new lesion target or any increase in new lesion non-target); the appearance of new lesions when none have previously been recorded, can also confirm iCPD |
| Independent blinded review and central collection of scans | Recommended in some circumstances—eg, in some trials with progression-based endpoints planned for marketing approval | Collection of scans (but not independent review) recommended for all trials |
| Confirmation of progression | Not required (unless equivocal) | Required |
| Consideration of clinical status | Not included in assessment | Clinical stability is considered when deciding whether treatment is continued after iUPD |

Table 1: Comparison of RECIST v1.1. and iRECIST, adapted from (19)

| | Timepoint response with no previous iUPD in any category | Timepoint response with previous iUPD in any category* |
|---|---|---|
| Target lesions: iCR; non-target lesions: iCR; new lesions: no | iCR | iCR |
| Target lesions: iCR; non-target lesions: non-iCR/non-iUPD; new lesions: no | iPR | iPR |
| Target lesions: iPR; non-target lesions: non-iCR/non-iUPD; new lesions: no | iPR | iPR |
| Target lesions: iSD; non-target lesions: non-iCR/non-iUPD; new lesions: no | iSD | iSD |
| Target lesions: iUPD with no change, or with a decrease from last timepoint; non-target lesions: iUPD with no change, or decrease from last timepoint; new lesions: yes | Not applicable | New lesions confirm iCPD if new lesions were previously identified and they have increased in size (≥5 mm in sum of measures for new lesion target or any increase for new lesion non-target) or number; if no change is seen in new lesions (size or number) from last timepoint, assignment remains iUPD |
| Target lesions: iSD, iPR, iCR; non-target lesions: iUPD; new lesions: no | iUPD | Remains iUPD unless iCPD is confirmed on the basis of a further increase in the size of non-target disease (does not need to meet RECIST 1.1 criteria for unequivocal progression) |
| Target lesions: iUPD; non-target lesions: non-iCR/non-iUPD, or iCR; new lesions: no | iUPD | Remains iUPD unless iCPD is confirmed on the basis of a further increase in sum of measures ≥5 mm; otherwise, assignment remains iUPD |
| Target lesions: iUPD; non-target lesions: iUPD; new lesions: no | iUPD | Remains iUPD unless iCPD is confirmed based on a further increase in previously identified target lesion iUPD in sum of measures ≥5 mm or non-target lesion iUPD (previous assessment need not have shown unequivocal progression) |
| Target lesions: iUPD; non-target lesions: iUPD; new lesions: yes | iUPD | Remains iUPD unless iCPD is confirmed on the basis of a further increase in previously identified target lesion iUPD sum of measures ≥5 mm, previously identified non-target lesion iUPD (does not need to be unequivocal), or an increase in the size or number of new lesions previously identified |
| Target lesions: non-iUPD or progression; non-target lesions: non-iUPD or progression; new lesions: yes | iUPD | Remains iUPD unless iCPD is confirmed on the basis of an increase in the size or number of new lesions previously identified |

Table 2: Assignment of timepoint response with iRECIST, adapted from (19)

## 17.4 Response Evaluation Criteria in Solid Tumors (RECIST)

Selected sections from the Response Evaluation Criteria in Solid Tumors (RECIST), Version 1.1 are presented below, with slight modifications and the addition of explanatory text as needed for clarity.

### 17.4.1 Measurability of tumor at baseline

**DEFINITIONS**
At baseline, tumor lesions/lymph nodes will be categorized measurable or non-measurable as follows:

**Measurable Tumor Lesions**
**Tumor Lesions.** Tumor lesions must be accurately measured in at least one dimension (longest diameter in the plane of measurement is to be recorded) with a minimum size of:

- 10 mm by computed tomography (CT) or magnetic resonance imaging (MRI) scan (CT/MRI scan slice thickness/interval no greater than 5 mm)
- 10-mm caliper measurement by clinical examination (lesions that cannot be accurately measured with calipers should be recorded as non-measurable)
- 20 mm by chest X-ray

**Malignant Lymph Nodes.** To be considered pathologically enlarged and measurable, a lymph node must be ≥ 15 mm in the short axis when assessed by CT scan (CT scan slice thickness recommended to be no greater than 5 mm). At baseline and in follow-up, only the short axis will be measured and followed. See also notes below on “Baseline Documentation of Target and Nontarget Lesions” for information on lymph node measurement.

**Non-Measurable Tumor Lesions**
Non-measurable tumor lesions encompass small lesions (longest diameter < 10 mm or pathological lymph nodes with ≥ 10 to < 15 mm short axis), as well as truly non-measurable lesions. Lesions considered truly non-measurable include leptomeningeal disease, ascites, pleural or pericardial effusion, inflammatory breast disease, lymphangitic involvement of skin or lung, peritoneal spread, and abdominal masses/abdominal organomegaly identified by physical examination that is not measurable by reproducible imaging techniques.

**Special Considerations Regarding Lesion Measurability**

Bone lesions, cystic lesions, and lesions previously treated with local therapy require particular comment, as outlined below.

Bone lesions:

- Bone scan, positron emission tomography (PET) scan, or plain films are not considered adequate imaging techniques to measure bone lesions. However, these techniques can be used to confirm the presence or disappearance of bone lesions.
- Lytic bone lesions or mixed lytic blastic lesions, with identifiable soft tissue components, that can be evaluated by cross-sectional imaging techniques such as CT or MRI can be considered measurable lesions if the soft tissue component meets the definition of measurability described above.
- Blastic bone lesions are non-measurable.

Cystic lesions:

- Lesions that meet the criteria for radiographically defined simple cysts should not be considered malignant lesions (neither measurable nor non-measurable) since they are, by definition, simple cysts.
- Cystic lesions thought to represent cystic metastases can be considered measurable lesions if they meet the definition of measurability described above. However, if noncystic lesions are present in the same patient, these are preferred for selection as target lesions.

Lesions with prior local treatment:

- Tumor lesions situated in a previously irradiated area, or in an area subjected to other loco-regional therapy, are usually not considered measurable unless there has been demonstrated progression in the lesion. Study protocols should detail the conditions under which such lesions would be considered measurable.

### 17.4.2 Target lesions: specifications by methods of measurements

**<u>Measurement of Lesions</u>**

All measurements should be recorded in metric notation, using calipers if clinically assessed. All baseline evaluations should be performed as close as possible to the treatment start and never more than 4 weeks before the beginning of the treatment.

**<u>Method of Assessment</u>**

The same method of assessment and the same technique should be used to characterize each identified and reported lesion at baseline and during study. Imaging-based evaluation should always be the preferred option.

**Clinical Lesions.** Clinical lesions will only be considered measurable when they are superficial and ≥ 10 mm in diameter as assessed using calipers (e.g., skin nodules).
For the case of skin lesions, documentation by color photography, including a ruler to estimate the size of the lesion, is suggested.

**Chest X-Ray.** Chest CT is preferred over chest X-ray, particularly when progression is an important endpoint, since CT is more sensitive than X-ray, particularly in identifying new lesions. However, lesions on chest X-ray may be considered measurable if they are clearly defined and surrounded by aerated lung.

**CT, MRI.** CT is the best currently available and reproducible method to measure lesions selected for response assessment. This guideline has defined measurability of lesions on CT scan based on the assumption that CT slice thickness is 5 mm or less. When CT scans have slice thickness greater than 5 mm, the minimum size for a measurable lesion should be twice the slice thickness. MRI is also acceptable.

If prior to enrollment it is known that a patient is unable to undergo CT scans with intravenous (IV) contrast due to allergy or renal insufficiency, the decision as to whether a noncontrast CT or MRI (without IV contrast) will be used to evaluate the patient at baseline and during the study should be guided by the tumor type under investigation and the anatomic location of the disease. For patients who develop contraindications to contrast after baseline contrast CT is done, the decision as to whether noncontrast CT or MRI (enhanced or nonenhanced) will be performed should also be based on the tumor type and the anatomic location of the disease and should be optimized to allow for comparison with the prior studies if possible. Each case should be discussed with the radiologist to determine if substitution of these other approaches is possible and, if not, the patient should be considered not evaluable from that point forward. Care must be taken in measurement of target lesions on a different modality and interpretation of nontarget disease or new lesions since the same lesion may appear to have a different size using a new modality.

**Ultrasound.** Ultrasound is not useful in the assessment of lesion size and should not be used as a method of measurement.

**Endoscopy, Laparoscopy, Tumor Markers, Cytology, Histology.** The utilization of these techniques for objective tumor evaluation cannot generally be advised.

### 17.4.3 Tumor response evaluation

#### ASSESSMENT OF OVERALL TUMOR BURDEN AND MEASURABLE DISEASE

To assess objective response or future progression, it is necessary to estimate the overall tumor burden at baseline and to use this as a comparator for subsequent measurements. Measurable disease is defined by the presence of at least one measurable lesion, as detailed above.

#### BASELINE DOCUMENTATION OF TARGET AND NONTARGET LESIONS

When more than one measurable lesion is present at baseline, all lesions up to a maximum of five lesions total (and a maximum of two lesions per organ) representative of all involved organs

should be identified as target lesions and will be recorded and measured at baseline. This means in instances where patients have only one or two organ sites involved, a maximum of two lesions (one site) and four lesions (two sites), respectively, will be recorded. Other lesions (albeit measurable) in those organs will be recorded as non-measurable lesions (even if the size is > 10 mm by CT scan).

Target lesions should be selected on the basis of their size (lesions with the longest diameter) and be representative of all involved organs, but additionally, should lend themselves to reproducible repeated measurements. It may be the case that, on occasion, the largest lesion does not lend itself to reproducible measurement, in which circumstance the next largest lesion that can be measured reproducibly should be selected.

Lymph nodes merit special mention since they are normal anatomical structures that may be visible by imaging even if not involved by tumor. As noted above, pathological nodes that are defined as measurable and may be identified as target lesions must meet the criterion of a short axis of ≥ 15 mm by CT scan. Only the short axis of these nodes will contribute to the baseline sum. The short axis of the node is the diameter normally used by radiologists to judge if a node is involved by solid tumor. Nodal size is normally reported as two dimensions in the plane in which the image is obtained (for CT scan, this is almost always the axial plane; for MRI the plane of acquisition may be axial, sagittal, or coronal). The smaller of these measures is the short axis. For example, an abdominal node that is reported as being 20 mm × 30 mm has a short axis of 20 mm and qualifies as a malignant, measurable node. In this example, 20 mm should be recorded as the node measurement. All other pathological nodes (those with short axis ≥ 10 mm but < 15 mm) should be considered nontarget lesions. Nodes that have a short axis < 10 mm are considered nonpathological and should not be recorded or followed.

A sum of the diameters (longest for non-nodal lesions, short axis for nodal lesions) for all target lesions will be calculated and reported as the baseline sum of diameters. If lymph nodes are to be included in the sum, then, as noted above, only the short axis is added into the sum. The baseline sum of diameters will be used as a reference to further characterize any objective tumor regression in the measurable dimension of the disease.

<u>All other lesions (or sites of disease), including pathological lymph nodes, should be identified as nontarget lesions and should also be recorded at baseline. Measurements are not required and these lesions should be followed as “present,” “absent,” or in rare cases “unequivocal progression.”</u>

In addition, it is possible to record multiple nontarget lesions involving the same organ as a single item on the Case Report Form (CRF) (e.g., “multiple enlarged pelvic lymph nodes” or “multiple liver metastases”).

**RESPONSE CRITERIA**

**<u>Evaluation of Target Lesions</u>**

This section provides the definitions of the criteria used to determine objective tumor response for target lesions.

- **Complete response (CR)**: disappearance of all target lesions
  Any pathological lymph nodes (whether target or nontarget) must have reduction in short axis to < 10 mm.
- **Partial response (PR)**: at least a 30% decrease in the sum of diameters of target lesions, taking as reference the baseline sum of diameters
- **Progressive disease (PD)**: at least a 20% increase in the sum of diameters of target lesions, taking as reference the smallest sum on study (nadir), including baseline.
  In addition to the relative increase of 20%, the sum must also demonstrate an absolute increase of at least 5 mm.
  The appearance of one or more new lesions is also considered progression.
- **Stable disease (SD)**: neither sufficient shrinkage to qualify for PR nor sufficient increase to qualify for PD, taking as reference the smallest sum on study

**<u>Special Notes on the Assessment of Target Lesions</u>**

**Lymph Nodes.** Lymph nodes identified as target lesions should always have the actual short axis measurement recorded (measured in the same anatomical plane as the baseline examination), even if the nodes regress to < 10 mm on study. This means that when lymph nodes are included as target lesions, the sum of lesions may not be zero even if CR criteria are met since a normal lymph node is defined as having a short axis < 10 mm.

**Target Lesions That Become Too Small to Measure.** While on study, all lesions (nodal and non-nodal) recorded at baseline should have their actual measurements recorded at each subsequent evaluation, even when very small (e.g., 2 mm). However, sometimes lesions or lymph nodes that are recorded as target lesions at baseline become so faint on the CT scan that the radiologist may not feel comfortable assigning an exact measure and may report them as being too small to measure. When this occurs, it is important that a value be recorded on the CRF as follows:

- If it is the opinion of the radiologist that the lesion has likely disappeared, the measurement should be recorded as 0 mm.
- If the lesion is believed to be present and is faintly seen but too small to measure, a default value of 5 mm should be assigned and below measurable limit (BML) should be ticked. (Note: It is less likely that this rule will be used for lymph nodes since they usually have a definable size when normal and are frequently surrounded by fat such as in the retroperitoneum; however, if a lymph node is believed to be present and is faintly seen but too small to measure, a default value of 5 mm should be assigned in this circumstance as well and BML should also be ticked.)

<u>To reiterate, however, if the radiologist is able to provide an actual measure, that should be recorded, even if it is below 5 mm, and, in that case, BML should not be ticked.</u>

**Lesions That Split or Coalesce on Treatment.** When non-nodal lesions fragment, the longest diameters of the fragmented portions should be added together to calculate the target lesion sum. Similarly, as lesions coalesce, a plane between them may be maintained that would aid in obtaining maximal diameter measurements of each individual lesion. If the lesions have truly coalesced such that they are no longer separable, the vector of the longest diameter in this instance should be the maximal longest diameter for the coalesced lesion.

**Evaluation of Nontarget Lesions**

This section provides the definitions of the criteria used to determine the tumor response for the group of nontarget lesions. While some nontarget lesions may actually be measurable, they need not be measured and, instead, should be assessed only qualitatively at the timepoints specified in the protocol.

- **CR**: disappearance of all nontarget lesions and (if applicable) normalization of tumor marker level). All lymph nodes must be non-pathological in size (< 10 mm short axis).
- **Non-CR/Non-PD**: persistence of one or more nontarget lesion(s) and/or (if applicable) maintenance of tumor marker level above the normal limits
- **PD**: unequivocal progression of existing nontarget lesions
  The appearance of one or more new lesions is also considered progression.

**Special Notes on Assessment of Progression of Nontarget Disease**

**When the Patient Also Has Measurable Disease.** In this setting, to achieve unequivocal progression on the basis of the nontarget disease, there must be an overall level of substantial worsening in nontarget disease in a magnitude that, even in the presence of SD or PR in target disease, the overall tumor burden has increased sufficiently to merit discontinuation of therapy. A modest increase in the size of one or more nontarget lesions is usually not sufficient to qualify for unequivocal progression status. The designation of overall progression solely on the basis of change in nontarget disease in the face of SD or PR of target disease will therefore be extremely rare.

**When the Patient Has Only Non-Measurable Disease.** This circumstance arises in some Phase III trials when it is not a criterion of study entry to have measurable disease.
The same general concepts apply here as noted above; however, in this instance, there is no measurable disease assessment to factor into the interpretation of an increase in non-measurable disease burden. Because worsening in nontarget disease cannot be easily quantified (by definition: if all lesions are truly non-measurable), a useful test that can be applied when assessing patients for unequivocal progression is to consider if the increase in overall disease burden based on the change in non-measurable disease is comparable in magnitude to the increase that would be required to declare PD for measurable disease, that is, an increase in tumor burden representing an additional 73% increase in volume (which is equivalent to a 20% increase in diameter in a measurable lesion). Examples include an increase in a pleural effusion from “trace” to “large” or an increase in lymphangitic disease from localized to widespread or may be described in protocols as “sufficient to require a change in therapy.” If unequivocal progression is seen, the patient should be considered to have had overall PD at that point. While it would be ideal to have objective criteria to apply to non-measurable disease, the very nature of that disease makes it impossible to do so; therefore, the increase must be substantial.

**New Lesions**

The appearance of new malignant lesions denotes disease progression; therefore, some comments on detection of new lesions are important. There are no specific criteria for the identification of new radiographic lesions; however, the finding of a new lesion should be unequivocal, that is, not attributable to differences in scanning technique, change in imaging modality, or findings thought

to represent something other than tumor (for example, some “new” bone lesions may be simply healing or flare of preexisting lesions). This is particularly important when the patient’s baseline lesions show partial or complete response. For example, necrosis of a liver lesion may be reported on a CT scan report as a “new” cystic lesion, which it is not.

A lesion identified during the study in an anatomical location that was not scanned at baseline is considered a new lesion and will indicate disease progression.

If a new lesion is equivocal, for example because of its small size, continued therapy and follow-up evaluation will clarify if it represents truly new disease. If repeat scans confirm there is definitely a new lesion, then progression should be declared using the date of the initial scan.

## EVALUATION OF RESPONSE

### Timepoint Response (Overall Response)

It is assumed that at each protocol-specified timepoint, a response assessment occurs. Table 1 provides a summary of the overall response status calculation at each timepoint for patients who have measurable disease at baseline.

When patients have non-measurable (therefore nontarget) disease only, Table 2 is to be used.

**Table 1 Timepoint Response: Patients with Target Lesions (with or without Nontarget Lesions)**

| Target Lesions | Nontarget Lesions | New Lesions | Overall Response |
|---|---|---|---|
| CR | CR | No | CR |
| CR | Non-CR/non-PD | No | PR |
| CR | Not evaluated | No | PR |
| PR | Non-PD or not all evaluated | No | PR |
| SD | Non-PD or not all evaluated | No | SD |
| Not all evaluated | Non-PD | No | NE |
| PD | Any | Yes or no | PD |
| Any | PD | Yes or no | PD |
| Any | Any | Yes | PD |

CR= complete response; NE= not evaluable; PD = progressive disease;
PR= partial response; SD = stable disease.

**Table 2 Timepoint Response: Patients with Nontarget Lesions Only**

| Nontarget Lesions | New Lesions | Overall Response |
|---|---|---|
| CR | No | CR |
| Non-CR/non-PD | No | Non-CR/non-PD [a] |
| Not all evaluated | No | NE |
| Unequivocal PD | Yes or no | PD |
| Any | Yes | PD |

CR= complete response; NE= not evaluable; PD = progressive disease.
[a] "Non-CR/non-PD" is preferred over "stable disease" for nontarget disease since stable disease is increasingly used as an endpoint for assessment of efficacy in some trials; thus, assigning "stable disease" when no lesions can be measured is not advised.

## Missing Assessments and Not-Evaluable Designation

When no imaging/measurement is done at all at a particular timepoint, the patient is not evaluable at that timepoint. If only a subset of lesion measurements are made at an assessment, usually the case is also considered not evaluable at that timepoint, unless a convincing argument can be made that the contribution of the individual missing lesion(s) would not change the assigned timepoint response. This would be most likely to happen in the case of PD. For example, if a patient had a baseline sum of 50 mm with three measured lesions and, during the study, only two lesions were assessed, but those gave a sum of 80 mm, the patient will have achieved PD status, regardless of the contribution of the missing lesion.

If one or more target lesions were not assessed either because the scan was not done or the scan could not be assessed because of poor image quality or obstructed view, the response for target lesions should be "unable to assess" since the patient is not evaluable. Similarly, if one or more nontarget lesions are not assessed, the response for nontarget lesions should be "unable to assess" except where there is clear progression. Overall response would be "unable to assess" if either the target response or the nontarget response is "unable to assess," except where this is clear evidence of progression as this equates with the case being not evaluable at that timepoint.

**Table 3 Best Overall Response When Confirmation Is Required**

| Overall Response at First Timepoint | Overall Response at Subsequent Timepoint | Best Overall Response |
|---|---|---|
| CR | CR | CR |
| CR | PR | SD, PD, or PR [a] |
| CR | SD | SD, provided minimum duration for SD was met; otherwise, PD |
| CR | PD | SD, provided minimum duration for SD was met; otherwise, PD |
| CR | NE | SD, provided minimum duration for SD was met; otherwise, NE |
| PR | CR | PR |
| PR | PR | PR |
| PR | SD | SD |
| PR | PD | SD, provided minimum duration for SD was met; otherwise, PD |
| PR | NE | SD, provided minimum duration for SD was met; otherwise, NE |
| NE | NE | NE |

CR= complete response; NE= not evaluable; PD = progressive disease; PR = partial response;
SD= stable disease.

[a] If a CR is truly met at the first timepoint, any disease seen at a subsequent timepoint, even disease meeting PR criteria relative to baseline, qualifies as PD at that point (since disease must have reappeared after CR). Best response would depend on whether the minimum duration for SD was met. However, sometimes CR may be claimed when subsequent scans suggest small lesions were likely still present and in fact the patient had PR, not CR, at the first timepoint. Under these circumstances, the original CR should be changed to PR and the best response is PR.

**Special Notes on Response Assessment**

When nodal disease is included in the sum of target lesions and the nodes decrease to “normal” size (< 10 mm), they may still have a measurement reported on scans. This measurement should be recorded even though the nodes are normal in order not to overstate progression should it be based on increase in size of the nodes. As noted earlier, this means that patients with CR may not have a total sum of “zero” on the CRF.

Patients with a global deterioration of health status requiring discontinuation of treatment without objective evidence of disease progression at that time should be reported as “symptomatic deterioration.” Every effort should be made to document objective progression even after discontinuation of treatment. Symptomatic deterioration is not a descriptor of an objective response; it is a reason for stopping study therapy. The objective response status of such patients

is to be determined by evaluation of target and nontarget disease as shown in Table 1, Table 2, and Table 3.

For equivocal findings of progression (e.g., very small and uncertain new lesions; cystic changes or necrosis in existing lesions), treatment may continue until the next scheduled assessment. If at the next scheduled assessment progression is confirmed, the date of progression should be the earlier date when progression was suspected.

In studies for which patients with advanced disease are eligible (i.e., primary disease still or partially present), the primary tumor should also be captured as a target or nontarget lesion, as appropriate. This is to avoid an incorrect assessment of complete response if the primary tumor is still present but not evaluated as a target or nontarget lesion.

## 17.5 EORTC QLQ-C15-PAL (version 1)

We are interested in some things about you and your health. Please answer all of the questions yourself by circling the number that best applies to you. There are no "right" or "wrong" answers. The information that you provide will remain strictly confidential.

Please fill in your initials: ______________________

Your birthdate (Day, Month, Year): ______________________

Today's date (Day, Month, Year): ______________________

---

| | | Not at All | A Little | Quite a Bit | Very Much |
|---|---|---|---|---|---|
| 1. | Do you have any trouble taking a short walk outside of the house? | 1 | 2 | 3 | 4 |
| 2. | Do you need to stay in bed or a chair during the day? | 1 | 2 | 3 | 4 |
| 3. | Do you need help with eating, dressing, washing yourself or using the toilet? | 1 | 2 | 3 | 4 |

| **During the past week:** | | Not at All | A Little | Quite a Bit | Very Much |
|---|---|---|---|---|---|
| 4. | Were you short of breath? | 1 | 2 | 3 | 4 |
| 5. | Have you had pain? | 1 | 2 | 3 | 4 |
| 6. | Have you had trouble sleeping? | 1 | 2 | 3 | 4 |
| 7. | Have you felt weak? | 1 | 2 | 3 | 4 |
| 8. | Have you lacked appetite? | 1 | 2 | 3 | 4 |
| 9. | Have you felt nauseated? | 1 | 2 | 3 | 4 |
| 10. | Have you been constipated? | 1 | 2 | 3 | 4 |
| 11. | Were you tired? | 1 | 2 | 3 | 4 |
| 12. | Did pain interfere with your daily activities? | 1 | 2 | 3 | 4 |
| 13. | Did you feel tense? | 1 | 2 | 3 | 4 |
| 14. | Did you feel depressed? | 1 | 2 | 3 | 4 |

**For the following questions please circle the number between 1 and 7 that best applies to you**

15. How would you rate your overall quality of life during the past week?

| 1 | 2 | 3 | 4 | 5 | 6 | 7 |
|---|---|---|---|---|---|---|
| Very poor | | | | | | Excellent |

## 17.6 Chalder Fatigue Questionnaire (FQ) and pain score

### SPØRRESKJEMA FORALICE-STUDIEN

**Fatigue**

Vi vil gjerne vite om du har følt deg sliten, svak eller i mangel av overskudd den siste måneden. Vennligst besvar ALLE spørsmålene ved å krysse av (X) for det svaret du synes passer best for deg. Vi ønsker at du besvarer alle spørsmålene selv om du ikke har hatt slike problemer. Vi spør om hvordan du har følt deg i det siste og ikke om hvordan du følte deg for lenge siden. Hvis du har følt deg sliten lenge, ber vi om at du sammenlikner deg med hvordan du følte deg sist du var bra. (Ett kryss på hver linje)

| | | | | |
|---|---|---|---|---|
| 1. Har du problemer med at du føler deg sliten? | ☐ Mindre enn vanlig | ☐ Ikke mer enn vanlig | ☐ Mer enn vanlig | ☐ Mye mer enn vanlig |
| 2. Trenger du mer hvile? | ☐ Nei, mindre enn vanlig | ☐ Ikke mer enn vanlig | ☐ Mer enn vanlig | ☐ Mye mer enn vanlig |
| 3 Føler du deg søvnig eller døsig? | ☐ Mindre enn vanlig | ☐ Ikke mer enn vanlig | ☐ Mer enn vanlig | ☐ Mye mer enn vanlig |
| 4. Har du problemer med å komme igang med ting? | ☐ Mindre enn vanlig | ☐ Ikke mer enn vanlig | ☐ Mer enn vanlig | ☐ Mye mer enn vanlig |
| 5. Mangler du overskudd? | ☐ Ikke i det hele tatt | ☐ Ikke mer enn vanlig | ☐ Mer enn vanlig | ☐ Mye mer enn vanlig |
| 6. Har du redusert styrke i musklene dine? | ☐ Ikke i det hele tatt | ☐ Ikke mer enn vanlig | ☐ Mer enn vanlig | ☐ Mye mer enn vanlig |
| 7. Føler du deg svak? | ☐ Mindre enn vanlig | ☐ Som vanlig | ☐ Mer enn vanlig | ☐ Mye mer enn vanlig |
| 8. Har du vansker med å konsentrere deg? | ☐ Mindre enn vanlig | ☐ Som vanlig | ☐ Mer enn vanlig | ☐ Mye mer enn vanlig |
| 9. Forsnakker du deg i samtaler? | ☐ Mindre enn vanlig | ☐ Ikke mer enn vanlig | ☐ Mer enn vanlig | ☐ Mye mer enn vanlig |
| 10. Er det vanskeligere å finne det rette ordet? | ☐ Mindre enn vanlig | ☐ Ikke mer enn vanlig | ☐ Mer enn vanlig | ☐ Mye mer enn vanlig |
| 11. Hvordan er hukommelsen din? | ☐ Bedre enn vanlig | ☐ Ikke verre enn vanlig | ☐ Verre enn vanlig | ☐ Mye verre enn vanlig |

12. Hvis du føler deg sliten for tiden, omtrent hvor lenge har det vart?

☐ Mindre enn en uke ☐ Mindre enn tre måneder ☐ Mellom tre og seks måneder ☐ Seks måneder eller mer

**Smerter**

Vennligst sett ring rundt det tallet som best angir hvor sterke smerter du har hatt i gjennomsnitt de siste 24 timene.

| Ingen smerter | 0 | 1 | 2 | 3 | 4 | 5 | 6 | 7 | 8 | 9 | 10 | Verst tenkelige smerter |
|---|---|---|---|---|---|---|---|---|---|---|---|---|

I hvor stor grad har behandling eller medisiner lindret smertene dine de siste 24 timene?
Vennligst sett en ring rundt det prosenttallet som viser hvor stor smertelindring du har fått.

| 0% | 10% | 20% | 30% | 40% | 50% | 60% | 70% | 80% | 90% | 100% |
|---|---|---|---|---|---|---|---|---|---|---|
| Ingen lindring | | | | | | | | | | Fullstendig lindring |
