# Supplementary material for: Atezolizumab plus anthracycline-based chemotherapy in metastatic triple-negative breast cancer: the randomized, double-blind phase 2b ALICE trial
Source: Nat Med. 2022 Dec 8;28(12):2573–83. doi: 10.1038/s41591-022-02126-1 (PMC9800277; doi:10.1038/s41591-022-02126-1)
Supplement: Supplementary file 2 — Reporting Summary [file 41591_2022_2126_MOESM2_ESM.pdf]

nature portfolio

Corresponding author(s): Jon Amund Kyte

Last updated by author(s): Nov 4, 2022

Reporting Summary

Nature Portfolio wishes to improve the reproducibility of the work that we publish. This form provides structure for consistency and transparency in reporting. For further information on Nature Portfolio policies, see our Editorial Policies and the Editorial Policy Checklist.

Statistics

For all statistical analyses, confirm that the following items are present in the figure legend, table legend, main text, or Methods section.

n/a	Confirmed	
☐	☒	The exact sample size (*n*) for each experimental group/condition, given as a discrete number and unit of measurement
☒	☐	A statement on whether measurements were taken from distinct samples or whether the same sample was measured repeatedly
☐	☒	The statistical test(s) used AND whether they are one- or two-sided<br>*Only common tests should be described solely by name; describe more complex techniques in the Methods section.*
☒	☐	A description of all covariates tested
☒	☐	A description of any assumptions or corrections, such as tests of normality and adjustment for multiple comparisons
☐	☒	A full description of the statistical parameters including central tendency (e.g. means) or other basic estimates (e.g. regression coefficient) AND variation (e.g. standard deviation) or associated estimates of uncertainty (e.g. confidence intervals)
☐	☒	For null hypothesis testing, the test statistic (e.g. *F*, *t*, *r*) with confidence intervals, effect sizes, degrees of freedom and *P* value noted<br>*Give P values as exact values whenever suitable.*
☒	☐	For Bayesian analysis, information on the choice of priors and Markov chain Monte Carlo settings
☒	☐	For hierarchical and complex designs, identification of the appropriate level for tests and full reporting of outcomes
☒	☐	Estimates of effect sizes (e.g. Cohen's *d*, Pearson's *r*), indicating how they were calculated

Our web collection on statistics for biologists contains articles on many of the points above.

Software and code

Policy information about availability of computer code

Data collection	Viedoc v.4 (Viedoc Technologies AB, Uppsala, Sweden)
Data analysis	R Version 4.1.3<br>survival (v 3.4)<br>survminer (v 0.4.9)<br>Stata SE (version 14)<br>FlowJo v 10.8 (Tree Star Inc, Ashland, OR, USA)<br>GraphPad Prism v 9.4.1 (GraphPad Software)<br>RCCCollector v1.6.0 (NanoString Technologies®)<br>nCounter Digital Analyzer (NanoString Technologies®)<br>GATK MarkDuplicatesSpark, GATK BaseRecalibrator and GATK ApplyBQSR as implemented in the nf-core/Sarek 2.7 pipeline<br>GATK DepthOfCoverage 4.2.5.0<br>Mutect 2<br>Strelka 2<br>BCFTools 1.9

For manuscripts utilizing custom algorithms or software that are central to the research but not yet described in published literature, software must be made available to editors and reviewers. We strongly encourage code deposition in a community repository (e.g. GitHub). See the Nature Portfolio guidelines for submitting code & software for further information.

Data

Policy information about availability of data

All manuscripts must include a data availability statement. This statement should provide the following information, where applicable:

- Accession codes, unique identifiers, or web links for publicly available datasets
- A description of any restrictions on data availability
- For clinical datasets or third party data, please ensure that the statement adheres to our policy

Any request for raw or analyzed data will be reviewed by the study team, and a response can be expected within 14 days. The data generated in this study is subject to patient confidentiality, and the transfer of data or materials will require approval from the Data Privacy Officer and Institutional Review Board at OUH, and from the Regional Committee for Medical and Health Research Ethics South-East Norway and the Research Ethics Committee in Denmark. Any shared data will be de-identified. Requests should be made to the corresponding author (jonky@ous-hf.no).

Human research participants

Policy information about studies involving human research participants and Sex and Gender in Research.

Reporting on sex and gender	Self-reported gender is recoded and supplied in table 1. All but one patient identified as female, this imbalance is due to the nature of the disease studied.
Population characteristics	The ALICE study recruited patients with mTNBC who were 18 years or older, with an Eastern Cooperative Oncology Group (ECOG) performance status of 0 or 1 who had received no more than one prior line of chemotherapy in the metastatic setting and had measurable disease according to iRECIST. For patients who had received (neo)adjuvant treatment with anthracyclines or cyclophosphamide, a disease-free interval of at least 12 months was required.<br>A total of 68 patients with mTNBC were randomized and started the allocated treatment (67 female, 1 male). The median age at randomization was 55.5 years. 69% of the patients had received prior neoadjuvant or adjuvant therapy and 26% of the population had primary metastatic disease. At enrollment 71% of the patients had an ECOG score of 0 and 59% patients had not received any prior metastatic chemotherapy. 46% of the patients had PD-L1 positive tumors assessed by the SP142 assay.
Recruitment	Between 24 AUG 2017 and 21 DEC 2021 patients were recruited at 5 different study sites (3 in Norway and 2 in Denmark). All patients were referred through the National Health Service in Norway and Denmark, to which all patients have equal access. Every referred patient was equally assessed using the eligibility criteria specified in the study protocol. The randomized double-blind design minimizes the risk of bias and the effect of any self-selection bias.
Ethics oversight	The trial was approved by the Regional Comittees for Medical Research Ethics South East Norway (EC ID: 14195) and in Denmark by The National Research Ethics Committee (EC ID: H-18018750).<br>The trial was also approved by The Norwegian Medicines Agency (ID: 16/11993-6), The Danish Medicines Agency (ID: 2018051636) and the Institutional review boards.<br>All patients provided written informed consent. Patients did not receive financial compensation.

Note that full information on the approval of the study protocol must also be provided in the manuscript.

Field-specific reporting

Please select the one below that is the best fit for your research. If you are not sure, read the appropriate sections before making your selection.

☒ Life sciences ☐ Behavioural & social sciences ☐ Ecological, evolutionary & environmental sciences

For a reference copy of the document with all sections, see nature.com/documents/nr-reporting-summary-flat.pdf

Life sciences study design

All studies must disclose on these points even when the disclosure is negative.

Sample size	The initial sample size was calculated based on a two-sided test with an alpha level of 10% and a power of 80% to detect an absolute reduction of 15 % in the proportion without progression or death at 15 months in the atezo-chemo arm compared to the placebo-chemo arm. The estimated sample size was 75 patients in an intention-to-treat population (45 in atezo-chemo, 30 in placebo-chemo) for all efficacy analyses, as well as for the safety assessment. Due to concerns that some patients with rapid disease progression might leave the study without response evaluation, or before an effect of the intervention could be expected, the protocol was amended to allow the enrollment of 75 patients in a per-protocol population, comprising patients who had received ≥ 4 doses of atezolizumab/placebo and ≥ 3 doses of PLD and could be evaluated for tumor response. PFS in the PD-L1+ PP population was added as a co-primary outcome in a later amendment. Enrollment was stopped on Dec 31, 2021, due to slow patient accrual after the introduction of atezolizumab/nab-paclitaxel as standard therapy for PD-L1+ mTNBC.
Data exclusions	No data were excluded from the analyses

Replication	Not applicable for the clinical data. Translational experiments on human samples were not replicated; this is stated in the manuscript.
Randomization	Participants were randomly assigned (2:3) to receive either placebo + chemotherapy (placebo-chemo) or atezolizumab + chemotherapy (atezo-chemo). Randomization was done by the investigators using an unstratified permuted block design through an interactive web response system implemented in the electronic case report form Viedoc (Viedoc Technologies AB, Uppsala, Sweden). Randomization listings were generated by an independent statistician at Clinical Trial Unit Research Support Services, Oslo University Hospital, using STATA 14 TM (StatCorpLP, College Station, TX, USA
Blinding	Participants, investigators, and study site personnel (other than pharmacy personnel involved in placebo/drug preparation) were blinded to the treatment assignment until database lock.

Reporting for specific materials, systems and methods

We require information from authors about some types of materials, experimental systems and methods used in many studies. Here, indicate whether each material, system or method listed is relevant to your study. If you are not sure if a list item applies to your research, read the appropriate section before selecting a response.

Materials & experimental systems

n/a	Involved in the study
☐	☒ Antibodies
☒	☐ Eukaryotic cell lines
☒	☐ Palaeontology and archaeology
☒	☐ Animals and other organisms
☐	☒ Clinical data
☒	☐ Dual use research of concern

Methods

n/a	Involved in the study
☒	☐ ChIP-seq
☐	☒ Flow cytometry
☒	☐ MRI-based neuroimaging

Antibodies

Antibodies used	The following antibodies were supplied by BD Bioscience (Norway): CD3-BUV395 (clone SK7, Cat. No. 564001), CD4-BV510 (clone SK3, Cat. No. 562970), CD19-BUV563 (clone SJ25C1, Cat. No 612916) CD56-AlexaFluor 647 (clone R19-760, Cat. No. 563443), and γδTCR-BUV737 (clone 11F2, Cat. No. 748533).<br>Antibodies for CD8-FITC (clone RPA-T8, Cat. No. 301050) and CD25.BV605 (clone BC96, Cat. No. 302632) were supplied by BioLegend (Nordic Biosite, Norway).<br>The Foxp3-PE (clone 236A/EH, Cat. No 12-4777-42) were supplied by Thermo Fisher Scientific.
Validation	All antibodies used were commercially available.

Clinical data

Policy information about clinical studies

All manuscripts should comply with the ICMJE guidelines for publication of clinical research and a completed CONSORT checklist must be included with all submissions.

Clinical trial registration	NCT03164993
Study protocol	Protocol paper publication: DOI: 10.1186/s12967-020-02424-7<br>A copy of the last version of the protocol is provided with this manuscript.
Data collection	Data was collected between August 24, 2017, and July 5, 2022, at the following sites:<br><br>Oslo University Hospital, Norway<br>Stavanger University Hospital, Norway<br>St. Olavs Hospital, Trondheim University Hospital, Norway<br>Rigshospitalet, Denmark<br>Vejle Hospital, Denmark
Outcomes	The co-primary endpoints were safety and progression-free survival (PFS). Safety was evaluated as the incidence, nature, and severity of adverse events. The secondary efficacy outcomes included overall survival (OS), objective response rate (ORR), duration of response (DoR), durable response rate (DRR), and clinical benefit rate (CBR).<br><br>PFS was defined as the time from randomization to disease progression according to iRECIST, as assessed by the investigator, or death from any cause. OS was defined as the time from randomization to death from any cause. ORR was defined as the proportion of patients with a partial or complete response by iRECIST. DoR was defined as the time from the first documentation of an objective response to the time of progression or death, and DRR as the proportion of patients with a DoR of ≥ 6 months. CBR was defined as the proportion of patients that had either an objective response or stable disease lasting at least until the radiological evaluation at 24 weeks +/- 7 days.

Flow Cytometry

Plots

Confirm that:

☒ The axis labels state the marker and fluorochrome used (e.g. CD4-FITC).

☒ The axis scales are clearly visible. Include numbers along axes only for bottom left plot of group (a 'group' is an analysis of identical markers).

☒ All plots are contour plots with outliers or pseudocolor plots.

☒ A numerical value for number of cells or percentage (with statistics) is provided.

Methodology

Sample preparation	PBMC from screening and Day 1 Cycle 5 timepoints were isolated from whole blood using LymphoPrep Cell Separation Media (Abbott Rapid Diagnostics AS, Oslo, Norway), frozen and stored in liquid nitrogen until assessed for immune cell populations by flow cytometry. PBMC were initially incubated with antibodies for the surface markers CD3-BUV395 (1:100), CD8-FITC (1:25), CD4-BV510 (1:100), γδ-TCR-BUV737 (1:100), CD19-BUV563 (1:25), CD56-AlexaFluor 647 (1:67 dilution; BD Bioscience, Oslo, Norway), CD25-BV605 (1:40 dilution; BioLegend, Nordic Biosite AS, Oslo, Norway) and Fixable Viability Dye eFluor780 (1:1000 dilution; Thermo Fisher Scientific, Oslo, Norway). After fixation and permeabilization using eBioscience™ Foxp3 / Transcription Factor Staining Buffer Set using manufacturers recommended protocol (Cat. No. 00-5523-00; Thermo Fisher Scientific), PBMC were incubated with an antibody for the intracellular antigen Foxp3-PE (1:25 dilution; Thermo Fisher Scientific). Samples were acquired using BD FACSymphony A5 flow cytometer (BD Biosciences, Franklin Lakes, NJ, USA) and the data analysed with FlowJo v10.8.1 (Tree Star Inc, Ashland, OR, USA) and GraphPad Prism v9.4.1 (GraphPad Software, San Diego, CA, USA) software.
Instrument	BD FACSymphony A5 flow cytometer (BD Biosciences, Franklin Lakes, NJ, USA),
Software	FlowJo v 10.8 (Tree Star Inc, Ashland, OR, USA)<br>GraphPad Prism v9.4.1 (GraphPad Software, San Diego, CA, USA)
Cell population abundance	NA (No cell sorting performed)
Gating strategy	Total PBMC were first gated on the basis of live/dead (Fixable Viability Dye eFluor780). Leukocytes were then determined by SSC-A vs FSC-A and doublet exclusion performed using SSC-A vs SSC-H. Immune cell subsets were defined as follows: CD4+ T cells (CD3+CD4+CD8-), CD8+ T cells (CD3+CD4-CD8+), Regulatory T cells (CD3+CD4+Foxp3+CD25Hi), B cells (CD3-CD19+), NK cells (CD3-CD56+), NKT cells (CD3+CD56+) and gd-T cells (CD3+gd-TCR+). CD25 and Foxp3 positive populations were defined using FMO to identify the negative populations.<br>Gating strategy is described in Supplementary Information. Further information on gating strategies are available on request.

☒ Tick this box to confirm that a figure exemplifying the gating strategy is provided in the Supplementary Information.
